# Microwave-Assisted Palladium Acetate-Catalyzed C–P Cross-Coupling of Arylboronic Acids and >P(O)H Reagents in the Absence of the Usual Mono- and Bidentate P-Ligands: Mechanistic Insights

**DOI:** 10.1021/acs.joc.3c01269

**Published:** 2023-08-09

**Authors:** Bianka Huszár, Zoltán Mucsi, György Keglevich

**Affiliations:** † Department of Organic Chemistry and Technology, Faculty of Chemical Technology and Biotechnology, Budapest University of Technology and Economics, 1521 Budapest, Hungary; ‡ Faculty of Materials and Chemical Sciences, University of Miskolc, 3515 Miskolc, Hungary

## Abstract

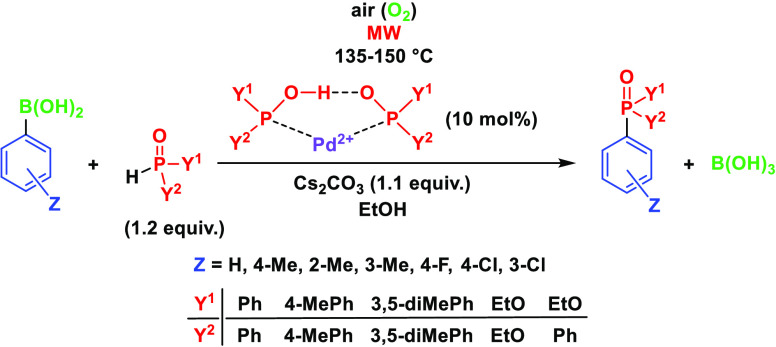

A less-studied halogen-free variation of the Hirao reaction involving the coupling of arylboronic acids with >P(O)H reagents, such as diarylphosphine oxides, diethyl phosphite, and ethyl phenyl-*H*-phosphinate, was investigated in detail using Pd(OAc)_2_ as the catalyst precursor and applying some excess of the P-reagent to supply the ligand via its trivalent tautomeric (>P–OH) form. The optimum conditions (1.2 equiv of the P-reagent, 135–150 °C, and air) were explored for the synthesis of diaryl-phenylphosphine oxides, aryl-diphenylphosphine oxides, diethyl arylphosphonates, ethyl diphenylphosphinate, and two bisphosphinoyl derivatives. In the reaction of 4-chlorophenyl- or 3-chlorophenylboronic acid with Ph_2_P(O)H, triphenylphosphine oxide was also formed as a byproduct. Theoretical calculations suggested that the catalytic cycle of the P–C coupling of PhB(OH)_2_ with Ph_2_P(O)H is different from that of the usual cross-coupling reactions. It comprises the addition of a phenyl anion and then the tautomeric form >P–OH of the >P(O)H reagent to the Pd^2+^ catalyst complex. This is then followed by reductive elimination affording Ph_3_PO that is accompanied with the conversion of Pd^2+^ to Pd^0^. There is a need for a subsequent stoichiometric oxidation of Pd(0) by molecular oxygen. The spontaneous formation of the self-assembling ligands around the Pd^2+^ center from the >P(O)H reactant plays a crucial role in the mechanism and promotes the efficiency of the catalyst.

## Introduction

A heteroatomic variation of the cross-coupling reactions is the C–P coupling discovered by Hirao in 1980.^1,2^ Since then, this new reaction between bromoarenes and dialkyl phosphites or secondary phosphine oxides providing dialkyl arylphosphonates or tertiary phosphine oxides has become an important method.^3−5^ The original Pd(PPh_3_)_4_ catalyst was replaced by Pd(II) salts used together with mono- and bidentate P-ligands, and the scope of the reaction components was extended.^6,7^ The mechanism was evaluated.^6,8^ Efforts were made to perform the P–C coupling under green chemical conditions.^9,10^ Later on, Ni- and Cu-catalyzed versions were also elaborated.^3,4,11,12^

The Hirao reaction of arylboronic acids with different >P(O)H reagents is a less-studied field. The coupling of arylboronic acids with diphenylphosphine oxide, ethyl phenyl-*H*-phosphinate, and diethyl phosphite was best performed at 90 °C for 24 h applying Pd(OAc)_2_/bis(diphenylphosphino)butane (dppb) as the catalyst system, K_2_CO_3_ as the base in 1,4-dioxane as the solvent with the use of air as an oxidant. The corresponding tertiary phosphine oxides, phosphinates, and phosphonates were obtained in 48–94% yields after a reaction time of 24 h.^13^ The second example for the Pd(II) salt-catalyzed case is the microwave (MW)-assisted reaction of arylboronic acids with diethyl phosphite in the presence of Pd(OAc)_2_/2,9-dimethyl-1,10-phenantroline (dmphen) in dimethylformamide. After an irradiation at 100 °C for 20–30 min, the dialkyl arylphosphonates were obtained in yields of 51–90%. The couplings were promoted by the addition of *p*-benzoquinone as an oxidating agent.^14^ A series of pyridylboronic acids also underwent the coupling reaction with dialkyl phosphites at 100 °C, in this case, using PdCl_2_/PPh_3_ as the catalyst precursor in *N*-,*N*-dimethylacetamide, in the presence of Ag_2_O as an additive. After a 2 h reaction time, the yields fell in the range of 51–95%.^15^ A NiBr_2_/pyridine-catalyzed coupling of arylboronic acids and mainly diarylphosphine oxides carried out at 100 °C for 24 h in dichloroethane in the presence of K_2_CO_3_ furnished the corresponding triarylphosphine oxides in variable 5–99% yields.^16^ Last but not least, Cu_2_O/1,10-phenantrolin (phen) and a special Cu(II) complex were also applied as catalysts at 26 °C for 24–72 h in the reaction under discussion to provide dialkyl arylphosphonates in variable yields (47–96%) using diisopropylethylamine in acetonitrile and KOAc in tetrahydrofuran, respectively.^17,18^ The disadvantages of the coupling reactions with arylboronic acids include the need, in most cases, for bidentate P-ligands or N-ligands, variable yields, and often long reaction times. In the Pd(II)/(bidentate) phosphine-catalyzed cases, the involvement of an oxidizing agent (even air) may seem somewhat unusual, as in the P–C coupling of bromoarenes, there was no need for this actor.

In the past few years, we developed a P–C coupling, where it was not necessary to add the usual mono- or bidentate P-ligands. Under MW conditions, the reaction between bromoarenes and >P(O)H reagents (dialkyl phosphites and secondary phosphine oxides) took place if the catalyst precursor, Pd(OAc)_2_ was combined with some excess of the P-reagent.^19^ The point was that the trivalent tautomeric form (>P–OH) of the >P(O)H species may act as a ligand to Pd(0). Upon applying 10% of the Pd(II) salt, there was a need for 30% extra quantity of the P-reagent. One equivalent of the >P(O)H species to the precursor ensured the Pd(II) → Pd(0) reduction, while the remaining two equivalent quantities served as the P-ligands to Pd(0).^20^ The detailed mechanism for the “P-ligand-free” Hirao reaction was explored by high-level quantum chemical calculations.^20,21^ It was found that the rate-determining step is the reagent insertion into the Pd complex or the Pd–C bond formation that may be promoted by MW assistance. Our procedure meant a green approach, as there was no need to add the usual expensive P-ligands; hence, cost and environmental burden can be saved. Regarding atomic efficiency, starting from arylboronic acids means an advantage over the variation applying bromoarenes, not speaking about the fact that the former protocol is halogen-free. It was a challenge for us to develop a new protocol combining the halogen-free option with our MW-assisted method lacking the use of the usual P-ligands. It was another challenge to justify the need for an oxidizing agent and to evaluate the mechanism.

## Results and Discussion

### Preparative Results

The coupling of phenylboronic acid with diphenylphosphine oxide was chosen as the basic model of the MW-assisted Pd-catalyzed process. 10 mol % Pd(OAc)_2_ was applied as the catalyst precursor at 135 °C using different bases in a quantity of 1.1 equiv and acetonitrile or ethanol as the solvent. The P-reagent was measured in a 1.2 equiv quantity in order to provide the two Ph_2_POH ligands. We worked at aerobic conditions (Table 1). The crude reaction mixtures were analyzed by ^31^P NMR and liquid chromatography–mass spectrometry (LC–MS). Performing the reaction in the presence of Cs_2_CO_3_ in acetonitrile, after a 2.5 h irradiation the conversion was 80% (Table 1, entry 1). The next run carried out in ethanol was complete after 1.5 h (Table 1, entry 2). In this case, 5% of ethyl diphenylphosphinate could be detected as a byproduct. 1,4-Dioxane was also a suitable solvent under similar conditions (Table 1, entry 3). Changing for K_2_CO_3_, a treatment at 135 °C for 2.5 h in acetonitrile also led to almost quantitative (96%) conversion (Table 1, entry 4). However, the accomplishment in ethanol was more efficient, as required a shorter irradiation time of 1.5 h (Table 1, entry 5). Only 3% of (EtO)Ph_2_PO contaminated the product (**1a**). The use of triethylamine as the base led, after a treatment of 1.5 h, in both solvents to low (11/22%) conversions (Table 1, entries 6 and 7). From the best experiments (marked by entries 2–4), triphenylphosphine oxide (**1a**) was obtained in 78–81% yields after flash column chromatography. Under an inert atmosphere, the P–C coupling did not take place.

**Table 1:**
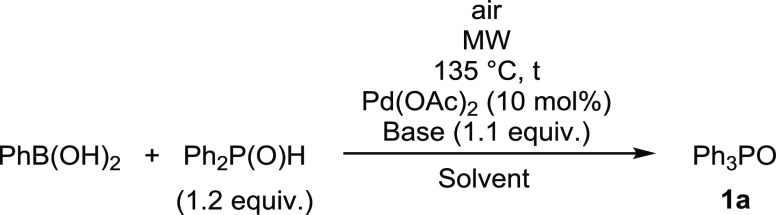
Optimization of the Coupling Reaction between Phenylboronic Acid and Diphenylphosphine Oxide

					product composition (%)^a^^,^^b^		
entry	base	solvent	*t* (h)	conversion (%)^a^	**1a**	(EtO)Ph_2_PO	yield (%) of **1a**
1	Cs_2_CO_3_	MeCN	2.5^c^	80	80		
2^c^	Cs_2_CO_3_	EtOH	1.5	100	95	5	83
3	Cs_2_CO_3_	dioxane	1.5	100	100		79
4	K_2_CO_3_	MeCN	2.5^d^	96	96		78
5	K_2_CO_3_	EtOH	1.5	100	97	3	80
6	Et_3_N	MeCN	1.5	11	11		
7	Et_3_N	EtOH	1.5	22	22		

a On the basis of relative ^31^P NMR intensities.

b The average of two or three parallel experiments.

c The coupling was also performed on a larger scale applying 1.0 mmol of PhB(OH)_2_ and 1.2 mmol of Ph_2_P(O)H. In this case, the yield of **1a** was 87%.

d The extrapolated reaction time is 3 h.

In general, the couplings were performed using 0.41 mmol of the phenylboronic acid. Carrying out the reaction on a 1.0 mmol scale, the yield of Ph_3_PO was 87% (footnote “c” of Table 1/entry 2). As a comparison, 10% Pd(PPh_3_)_4_ was also tested in the coupling reaction of phenylboronic acid with Ph_2_P(O)H at 135 °C for 1.5 h using 1.1 equiv of Cs_2_CO_3_ in ethanol. In this case, the reaction was not entirely clear-cut, and the yield of Ph_3_PO was 65%.

In the next series of experiments, the reaction of phenylboronic acid with different >P(O)H reagents was investigated at 135 °C applying 10 mol % Pd(OAc)_2_ as the catalyst precursor. The base/solvent combinations included Cs_2_CO_3_/EtOH and K_2_CO_3_/MeCN (Table 2). The coupling with bis(4-methylphenyl)phosphine oxide was somewhat slower than that with Ph_2_P(O)H, no matter if it was carried out using Cs_2_CO_3_ in EtOH or K_2_CO_3_ in MeCN (Table 2, entries 3 and 4 vs entries 1 and 2). After an irradiation of 2 and 3 h, respectively, the conversion was complete. Beyond the 85/81% proportion of the expected bis(4-methylphenyl)-phenylphosphine oxide (**1b**), there was 15/19% of tris(4-methylphenyl)phosphine oxide as the byproduct (Table 2, entries 3 and 4). A similar situation was observed, when bis(3,5-dimetylphenyl)phosphine oxide was the reagent; however, in this instance, the coupling became even slower, as the complete conversion required a reaction time of 2.5 and 4 h, respectively (Table 2, entries 5 and 6). Diaryl-phenylphosphine oxide **1c** was formed in 88 and 79%, respectively. The corresponding tris(3,5-dimethylphenyl)phosphine oxide was present in the mixture as a minor (12/21%) byproduct. From the best experiments, the diaryl-phenylphosphine oxides **1a–c** were isolated in yields of 74–83% after chromatography. Changing for diethyl phosphite, the coupling with phenylboronic acid was slower at 135 °C, as the completion took 4 h (Table 2, entry 7). However, at 150 °C, an irradiation of 0.5 h was enough (Table 2, entry 8). In both cases, the reaction was clear-cut, and diethyl phenylphosphonate (**1d**) was obtained in 84/82% yield after purification. The combination of K_2_CO_3_/MeCN was, in this case, less efficient: after an irradiation at 135 °C for 4 h, the conversion was only 63% (Table 2, entry 9). However, at 150 °C/2 h, phosphonate **1d** was the only product prepared in 73% yield (Table 2, entry 10). The next >P(O)H reagent, ethyl phenyl-*H*-phosphinate behaved similarly as (EtO)_2_P(O)H, and complete conversion could be attained after 4 h at 135 °C or 0.5 h at 150 °C (Table 2, entries 11 and 12). In the latter instance, 9% of (EtO)_2_PhPO also appeared in the crude mixture. Ethyl diphenylphosphinate (**1e**) was isolated in 82/86% yields.

**Table 2:**
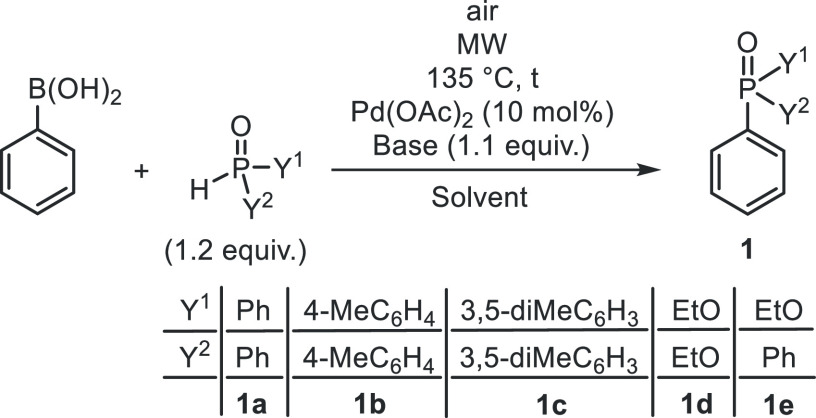
Coupling Reaction between Phenylboronic Acid and Different >P(O)H Reagents

						product composition (%)^a^^,^^b^		
entry	Y^1^, Y^2^	base	solvent	*t* (h)	conversion (%)^a^	**1**	Ar_3_PO	yield (%)
1	Ph	Cs_2_CO_3_	EtOH	1.5	100^b^	95^c^		83 (**1a**)
2	Ph	K_2_CO_3_	MeCN	2.5	96	96		78 (**1a**)
3	4-MeC_6_H_4_	Cs_2_CO_3_	EtOH	2	100	85	15	78 (**1b**)
4	4-MeC_6_H_4_	K_2_CO_3_	MeCN	3	100	81	19	72 (**1b**)
5	3,5-diMeC_6_H_3_	Cs_2_CO_3_	EtOH	2.5	100	88	12	74 (**1c**)
6	3,5-diMeC_6_H_3_	K_2_CO_3_	MeCN	4	100	79	21	56 (**1c**)
7	EtO	Cs_2_CO_3_	EtOH	4	100	100		84 (**1d**)
8	EtO	Cs_2_CO_3_	EtOH	0.5^d^	100	100		82 (**1d**)
9	EtO	K_2_CO_3_	MeCN	4	63	63		
10	EtO	K_2_CO_3_	MeCN	2^d^	100	100		73 (**1d**)
11	EtO, Ph	Cs_2_CO_3_	EtOH	4	100	100		82 (**1e**)
12	EtO, Ph	Cs_2_CO_3_	EtOH	0.5^d^	100	91^e^		86 (**1e**)

a On the basis of relative ^31^P NMR intensities.

b The average of two or three parallel experiments.

c 5% of (EtO)Ph_2_PO as the byproduct.

d The temperature was 150 °C.

e 9% of (EtO)_2_PhPO as the byproduct.

In the next round, Ph_2_P(O)H was reacted with a series of arylboronic acids, in the first approach, under the conditions applied above using Cs_2_CO_3_ in EtOH (Table 3). The coupling of 4-Me, 2-Me, 3-Me, and even the 4-F-substituted phenylboronic acids took place similarly as that with the unsubstituted PhB(OH)_2_. Complete conversions were attained after an irradiation of 1.5 h at 135 °C. The proportion of the expected products (**2f**–**i**) was 71–87%, while that of the (EtO)Ph_2_PO was 5–13%. Compounds **2f**–**i** were prepared in 67–80% yield (Table 3, entries 2–5). It was surprising that the similar reaction of 4-chlorophenylboronic acid with 1.2 equiv of Ph_2_P(O)H at 135 °C/1.5 h afforded 1,4-bis(diphenylphosphinoyl)benzene **3A**. The crude mixture contained 49% of bisphosphinoyl compound **3A** and 51% of Ph_3_PO (Table 3, entry 6). There was no expected 4-chlorophenyl-diphenylphosphine oxide **2j** in the mixture. Product **3A** was isolated by chromatography in a yield of 23%. To promote the formation of the phosphinoyl-chlorobenzene **2j**, the coupling was performed at a lower temperature of 90 °C for 4 h. Sure enough, the mixture comprised 46% of the monophosphinoyl product **2j**, 5% of bisphosphinoyl **3A**, 11% of (EtO)Ph_2_PO, and 38% of Ph_3_PO formed probably by dechlorination of 4-chlorophenyl-diphenylphosphine oxide (**2j**) (Table 3, entry 7). The small quantity of bisphosphinoyl compound **3A** among the components referred to the crucial role of temperature on the selectivity toward the mono- and bisphosphinoylated products (**2j** and **3A**). Repeating the previous P–C coupling on conventional heating at 90 °C/4 h, the conversion remained incomplete (77%), and the proportion of species **2j**, **3A**, and Ph_3_PO was 12, 41, and 18%, respectively, suggesting that the competitive dechlorination of 4-chlorophenyl-diphenylphosphine oxide (**2j**) was suppressed, and hence, more bis(Ph_2_P(O)) product (**3A**) could be formed (Table 3, entry 8). Returning to the result of the experiment marked by Table 3, entry 6, the low yield of 23% of the selective phosphinoylation may be explained by the fact that the 1.2 equiv quantity of Ph_2_P(O)H was not enough. Considering that a quantitative bisphosphinoylation would require 2.4 equiv of Ph_2_P(O)H, an experiment was carried out using this higher amount of the P-reactant. However, under such conditions, the proportion of bis-product **3A** was not increased, and the excess of Ph_2_P(O)H was involved in side reactions. In respect of the 3-chlorophenylboronic acid, the outcome of the P–C coupling with Ph_2_P(O)H was similar to the reaction of the 4-chlorophenyl reagent. After an irradiation at 135 °C for 1.5 h, only 53% of bisphosphinoyl derivative **3B**, 6% of (EtO)Ph_2_PO, and 41% of Ph_3_PO were present (Table 3, entry 10). The major component (**3B**) was isolated in a yield of 25%. Repeating the Hirao reaction at 90 °C for 4 h, the monophosphinoyl compound **2k** was also present in 35%, beyond the 22% proportion of bis derivative **3B** (Table 3, entry 11), and hence, the significant effect of the temperature was confirmed. In the thermal variation carried out, this occasion at 90 °C for 22 h, the proportion of 1,3-bis(diphenylphosphinoyl)benzene (**3B**) was increased to 44% (Table 3, entry 12). It can be noted that on MW irradiation, the statistically occurring local overheatings^22^ may cause the more intensive dechlorination of the primarily formed chlorophenyl-diphenylphosphine oxides (**2j** and **2k**).

**Table 3:**
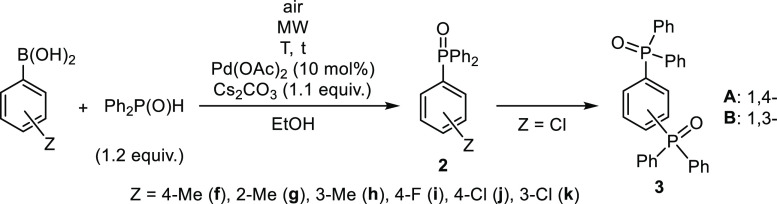
P–C Coupling of Substituted Arylboronic Acids with Diphenylphosphine Oxide

					product composition (%)^a^^,^^b^				
entry	Z	*T*	*t* (h)	conversion (%)^a^	**2**	**3**	(EtO)Ph_2_PO	Ph_3_PO (**1a**)	yield (%)
1	H	135	1.5	100^b^	95 (**1a**)		5		83 (**1a**)
2	4-Me (**f**)	135	1.5	100	81		5	14	73 (**2f**)
3	2-Me (**g**)	135	1.5	100	71		13	16	67 (**2g**)
4	3-Me (**h**)	135	1.5	100	87		8	5	80 (**2h**)
5	4-F (**i**)	135	1.5	100	75		10	15	70 (**2i**)
6	4-Cl (**j**)	135	1.5	100		49 (**3A**)		51	23 (**3A**)
7	4-Cl (**j**)	90	4	100	46	5	11	38	(**2j**)^c^
8	4-Cl (**j**)	90^d^	4	77	12	41 (**3A**)	6	18	
9	4-Cl (**j**)	135^e^	1	100		23 (**3A**)	5	72	
10	3-Cl (**k**)	135	1.5	100		53 (**3B**)	6	41	25 (**3B**)
11	3-Cl (**k**)	90	4	96	35	22 (**3B**)	11	28	(**2k**)^f^
12	3-Cl (**k**)	90^c^	22	100	33	44 (**3B**)	4	19	

a On the basis of relative ^31^P NMR intensities of the P-components.

b The average of two or three parallel experiments.

c ^31^P NMR (CDCl_3_) δ 28.5, δ_P_ lit.^23^ (CDCl_3_) 28.2; HRMS (m/z): calcd for C_18_H_15_OPCl [M + H]^+^, 313.0549; found, 313.0542.

d On conventional heating.

e 2.4 equiv of Ph_2_P(O)H was used.

f ^31^P NMR (CDCl_3_) δ 28.1, δ_P_ lit.^24^ (CDCl_3_) 28.1; HRMS (m/z): calcd for C_18_H_15_OPCl [M + H]^+^, 313.0549; found, 313.0538.

Finally, the substituted arylboronic acids were reacted with diethyl phosphite measured in a 1.2 equiv quantity using Cs_2_CO_3_ as the base and EtOH as the solvent (Table 4). The couplings with 4-MePh-, 2-MePh-, 3-MePh-, and 4-FPh-boronic acids were efficient on irradiation at 150 °C for 30 min. The diethyl arylphosphonates (**1d** and **4f**–**i**) were isolated in yields of 77–83% (Table 4, entries 1–7). The similar reactions of the 4-ClPh- and 3-ClPh-boronic acids carried out under similar conditions were accompanied by the formation of 19% and 16% of (EtO)_2_PhPO (**1d**), respectively, and the expected products **4j** and **4k** could be obtained in 66/71% yields. The reductive dechlorination was a side reaction also in this case.

**Table 4:**
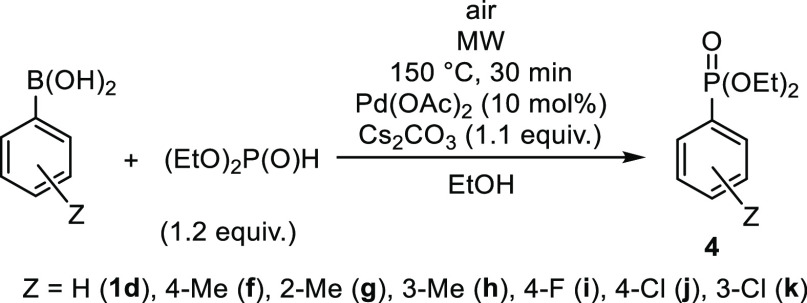
Hirao Reaction between Substituted Arylboronic Acids and Diethyl Phosphite

entry	Z	conversion (%)^a^	proportion of **4** (%)^a^^,^^b^	yield (%)
1	H	100	95	83 (**1d**)
2	4-Me	100	100	77 (**4f**)
3	2-Me	100	100	78 (**4g**)
4	3-Me	100	100	80 (**4h**)
5	4-F	100	100	77 (**4i**)
6	4-Cl	100^c^	81	66 (**4j**)
7	3-Cl	100^d^	84	71 (**4k**)

a On the basis of relative ^31^P NMR intensities.

b The average of two or three parallel experiments.

c 19% of (EtO)_2_PhPO as the byproduct.

d 16% of (EtO)_2_PhPO as the byproduct.

The diaryl-phenylphosphine oxides (**1a**–**c**), aryl-diphenylphosphine oxides (**2f**–**i**), diethyl arylphosphonates (**1d**, **4f**–**k**), ethyl diphenylphosphinate (**1e**), and bisphosphinoyl derivatives **3A** and **3B**, all together 17 compounds, were prepared by the method developed, and they were fully characterized by ^31^P, ^13^C and ^1^H NMR, as well as HRMS. The resulting phosphine oxides and related compounds may be utilized as P-ligands after deoxygenation.

### Theoretical Calculations

**Figure 1:**
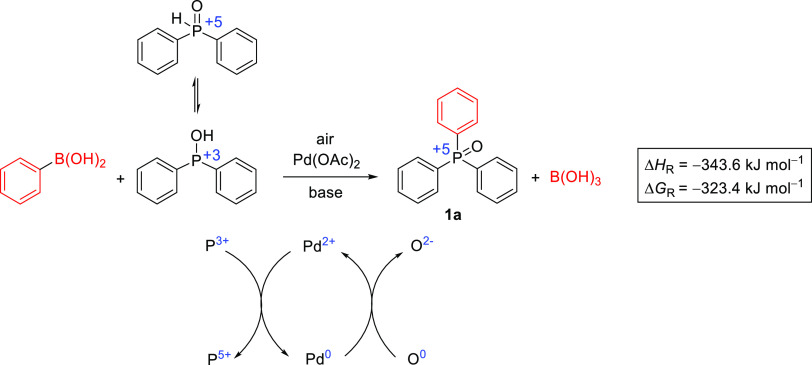
Overall transformation and reaction enthalpy (Δ*H*_R_), as well as Gibbs free energy (Δ*G*_R_) for the Hirao reaction of Ph_2_P(O)H with phenylboronic acid.

There are only a few examples in the literature for Hirao reactions starting from arylboronic acids. There is no detailed mechanistic study for this version of the P**–**C coupling, and only a putative cycle was described.^13^ However, it occurred that an oxidant is needed to promote the reaction.^13−15^ Looking at the overall reaction, the +3 oxidation number of the P atom in the tautomeric form of diphenylphosphine oxide is increased to +5 in the product triphenylphosphine oxide (**1a**). At the same time, the oxidation numbers of the boron and carbon atoms of the boronic acid remains unchanged. The main process is exothermic (Figure 1). We confirm that during the course of the reaction, Pd^2+^ is reduced to Pd^0^ that is then oxidized back by the oxygen present. The O_2_ is reduced from the state 0 to −2 during the transformation.

**Figure 2:**
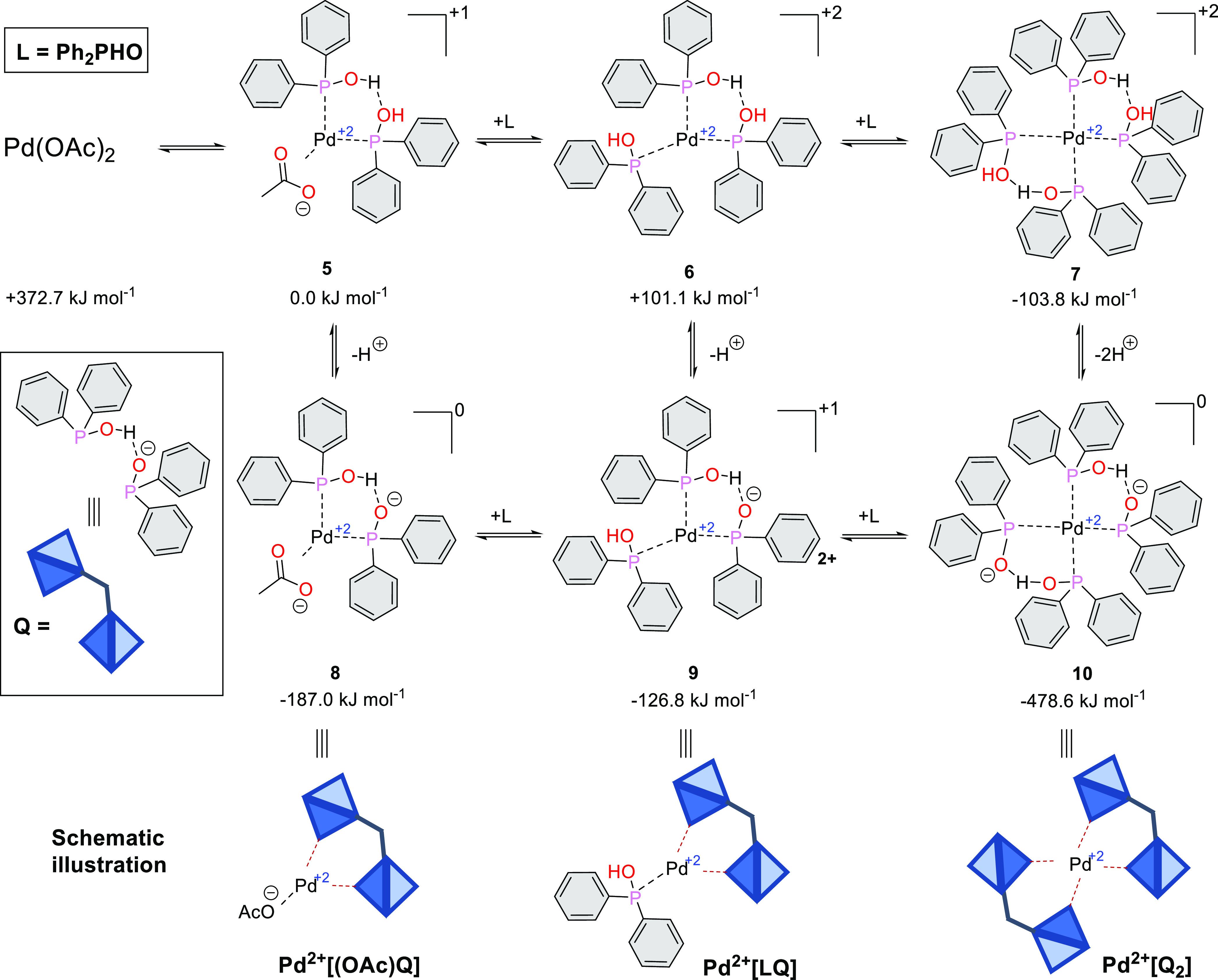
Equilibrium system of complexes formed from Pd^2+^ and Ph_2_P(O)H.

First, we explored a complex equilibrium of Pd(OAc)_2_ in the presence of excess of Ph_2_P(O)H as the ligand (L), including two-, three-, and tetraligations to afford species **5**, **6**, and **7**, respectively. The tautomeric form Ph_2_POH of Ph_2_P(O)H is able to organize a strong hydrogen bond net around the Pd^2+^. An inorganic base (e.g., K_2_CO_3_) may deprotonate one or two Ph_2_POH units to provide complexes **8**, **9**, and **10**, and as a consequence, the bonded Ph_2_POH pairs are stabilized, forming one (as in **8** or **9**) or two (as in **10**) quasi bidentate ligands (Figure 2). This spontaneous arrangement around the Pd^2+^ ion may be considered, as if a self-assembling bidentate P-ligand or two bidentate P-ligands were (indicated as Q; blue diamonds) around the central Pd^2+^ion. Formation of the tetraligated Pd^2+^ complex (**10**) is the most favorable in the series. This quasi bidentate anionic ligands activate the Pd^2+^ center for the coupling reaction.

**Figure 3:**
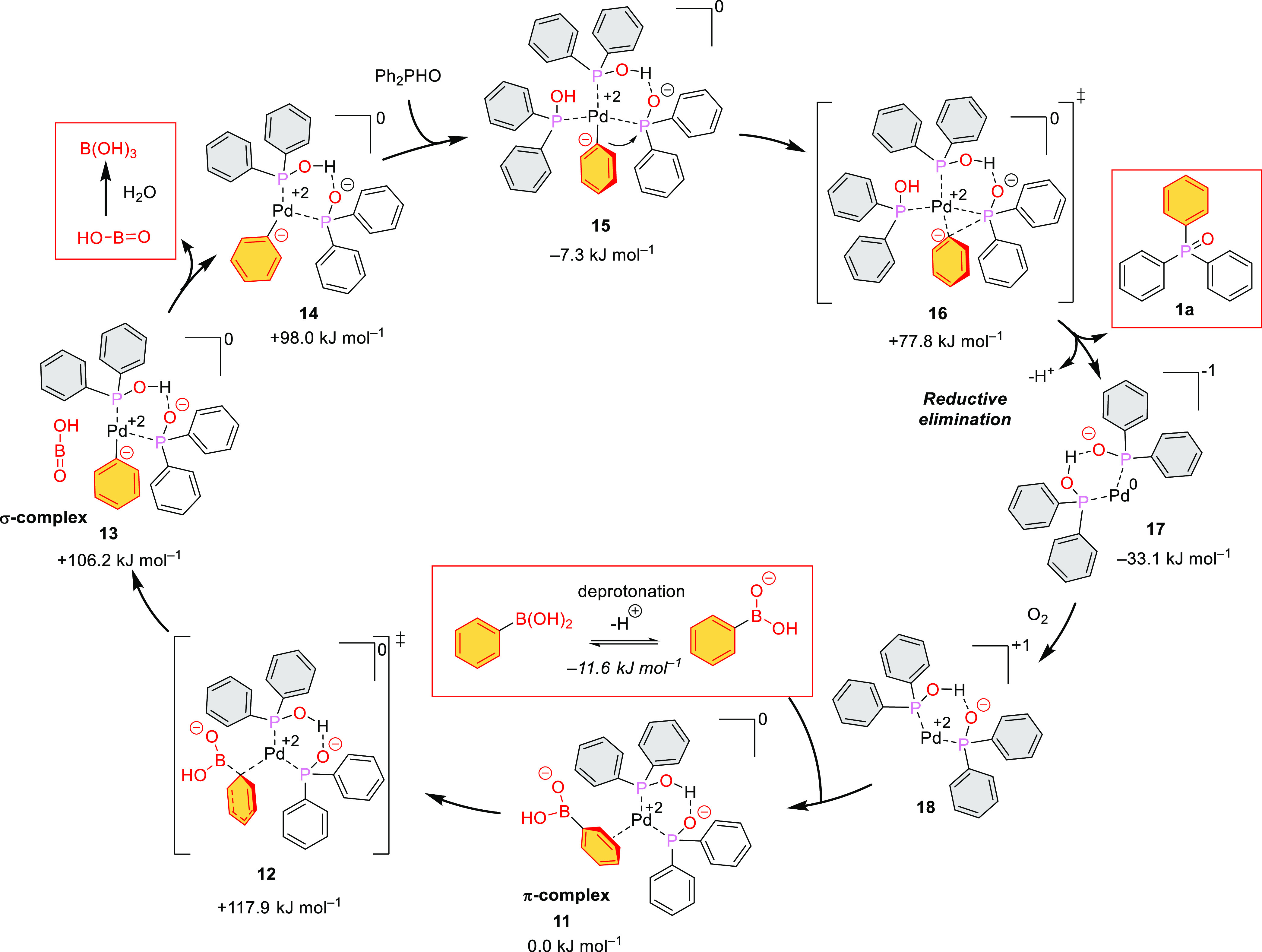
Formation of the desired triphenylphosphine oxide (**1a**) via a Pd-catalyzed reaction cycle.

**Figure 4:**
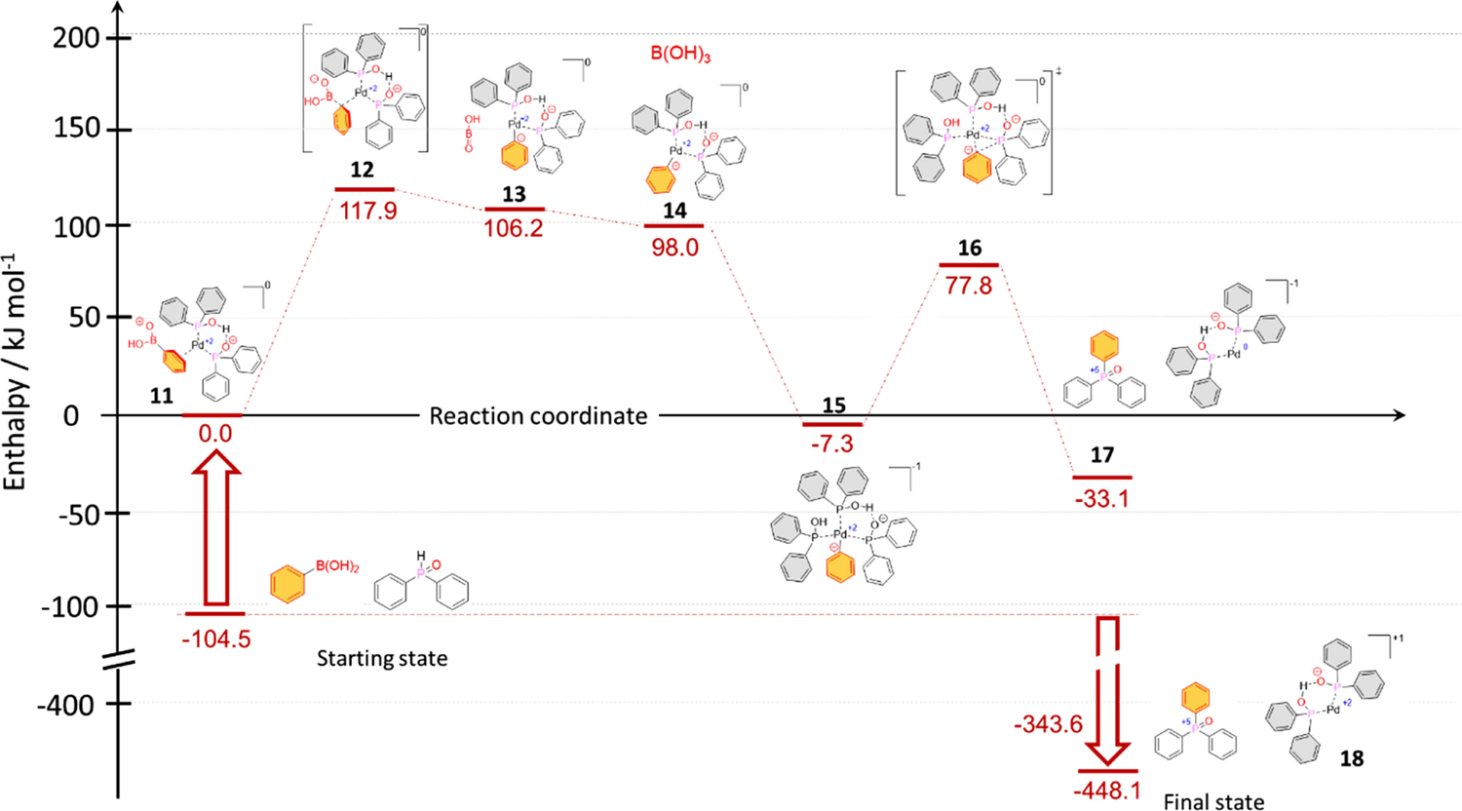
Enthalpy diagram for the Pd(OAc)_2_-catalyzed P–C coupling reaction of phenylboronic acid and diphenylphosphine oxide.

Then, we studied the catalytic cycle (Figure 3). In the first step, the boronic acid is deprotonated by the base present. Then, a weak π-complex (**6**) is formed between the Pd^2+^ center of the catalyst complex and the aryl ring of the boronic acid anion (Δ*H* = −11.6 kJ mol^–1^). The π-complex (**11**) is transformed to the σ-complex (**13**) via a well-determined TS (**12**) with a +117.9 kJ mol^–1^ enthalpy gap, which can be considered as a moderate barrier at elevated reaction temperature. The resulting σ-complex with an aryl anion (**13**) is of a high enthalpy content (106.2 kJ mol^–1^). The elimination of metaboric acid (HOBO) is favorable, but the entry of an additional Ph_2_POH ligand leading to complex **15** with an enthalpy of −7.3 kJ mol^–1^ is the real driving force. In the next step, the aryl anion is transferred from the central Pd^2+^ to the P atom of the adjacent ligand via TS **16** with a lower enthalpy content of 77.8 kJ mol^–1^ yielding the final tiphenylphosphine oxide (**1a**). During the reductive elimination step, the Pd^2+^ is converted to Pd^0^. Finally, the Pd^0^ is regenerated to Pd^2+^ by reaction with the O_2_ of air. Then, the catalytic cycle may start again. The enthalpy diagram for the P–C coupling under discussion can be seen in Figure 4. The suggested mechanism comprises two steps (involving transition states **12** and **16**), which have impact on the overall reaction rate. As the first step leading to transition state **12** exhibits the higher enthalpy barrier, the route via species **12** is the rate-determining step. The overall reaction enthalpy follows a slightly exothermic run until the formation of the triphenylphosphine oxide (**1a**) that is connected with the elimination of Pd^0^ complex **17**. However, the subsequent reoxidation of Pd^0^ complex **17** to Pd^2+^ catalyst **18** with the oxygen of air is rather exothermic, making the “quasi” catalytic process irreversible.

**Figure 5:**
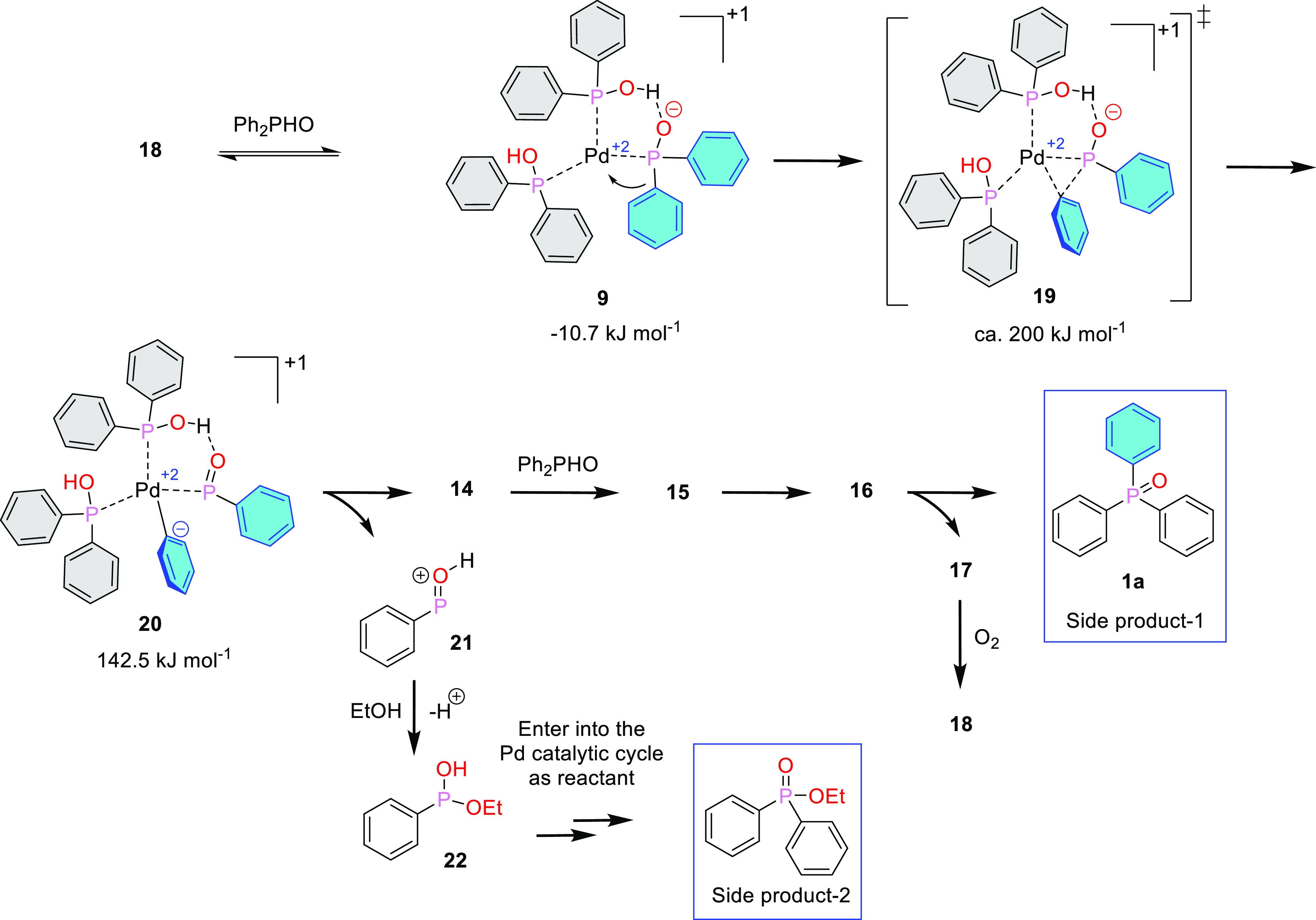
Proposed route for the formation of the triphenylphosphine oxide (**1a**) and ethyl diphenylphosphine oxide side products.

During the P–C coupling of Ph_2_P(O)H and arylboronic acids, a significant amount of triphenylphosphine oxide (**1a**) was also formed beside the expected aryl-diphenylphosphine oxide (**2f**–**k**) as discussed above (Table 3). In the case of 4-chlorophenyl- and 3-chlorophenylboronic acid, the dehalogenation by the Pd catalyst may also be a reasonable side reaction; however, for the 4-, 2- and 3-methylphenyl- and 4-fluorophenylboronic acid derivatives, the question arises how the triphenylphosphine oxide (**1a**) byproduct may be formed. Well, catalyst complex **18** may take up a Ph_2_POH ligand to give complex **9** (Figure 5). Migration of the phenyl ring from the Pd^2+^ to the adjacent P atom via transition state **19** leads to key intermediate **20**, which then enters into the previously discussed catalytic process, yielding triphenylphosphine oxide (**1a**) without the involvement of the arylboronic acid. The activation enthalpy for the **9** → **20** transformation is *ca* 200 kJ mol^–1^ that is significantly higher than the activation barrier of the rate-determining step of the main reaction (117.9 kJ mol^–1^). For this, the side reaction remains a minor component. The other byproduct, Ph_2_P(O)OEt may be formed by the P–C coupling of Ph(OEt)(OH) (**22**) with Ph_2_P(O)H. Species **22** is the result of the interaction of intermediate **21** and ethanol.

## Conclusions

In summary, the less applied, halogene-free Hirao reaction of arylboronic acids and a series of >P(O)H reagents, such as diarylphosphine oxides, diethyl phosphite, and ethyl phenyl-*H*-phosphinate, was studied in detail. Another green aspect was that not traditional (cost-meaning and environment-burdening) mono- or bidante P-ligands were used to serve the P-ligand to the Pd(OAc)_2_ precursor (10%) but the excess (20%) of the >P(O)H reagent via its >P–OH trivalent tautomer form. MW assistance enhanced the P–C couplings that were optimized for the different substituted cases. Theoretical calculations supported that there is no oxidative addition step, as the primary Pd^2+^/>P–OH···^–^O–P< complex remains unchanged during the addition of the aryl anion and the addition of the tautomer form of the P-reagent. In the last reductive elimination step, the central Pd^2+^ ion of the catalyst is reduced to Pd^0^. For this, it is necessary to apply a stoichiometric oxidant, that is, in our case, air. The ligation of Pd^2+^ with two >P(O)H units that form a dimer-like associate enhances the reactivity of the central Pd^2+^ in complexation with the aryl anion. Formation of the typical byproducts, e.g., (EtO)Ph_2_PO and Ph_3_PO, the latter formed also by dechlorination, was also explained.

## Experimental Section

### General Information

The reactions were carried out in a CEM Discover Model SP (300 W) focused microwave reactor (CEM Microwave Technology Ltd., Buckingham, U.K.) equipped with a stirrer and a pressure controller using 80–100 W irradiation under isothermal conditions. The reaction mixtures were irradiated in sealed glass vessels (with a volume of 10 mL) available from the supplier of CEM. The reaction temperature was monitored by an external IR sensor.

The ^31^P, ^13^C, and ^1^H NMR spectra were taken on a Bruker Avance 300/Avance 500 spectrometer (Rheinstetten, Germany) operating at 121.5/202.4, 75.5/125.7, and 300/500 MHz, respectively, in CDCl_3_ solution. The ^31^P chemical shifts are downfield relative to H_3_PO_4_, while the ^13^C and ^1^H chemical shifts are downfield relative to TMS. The couplings are given in Hz. The exact mass measurements were performed using an Agilent 6545 Q-TOF mass spectrometer (Santa Clara, CA) in high-resolution, positive electrospray mode. The melting points of products **1a**, **1b**, **1c**, **2f**, **2g**, **2h**, **2i**, and **3A** were determined using a Setaram differential scanning calorimetry 92 device.

### General Procedure for the P–C Coupling of Phenylboronic Acid and >P(O)H Reagents (Table 2, Entries **1**, **3**, **5**, **8**, and **12**)

To a MW glass vessel were added 1 mL of ethanol, 0.038 mmol (0.0085 g) of Pd(OAc)_2_, 0.41 mmol (0.050 g) of phenylboronic acid, 0.49 mmol of diarylphosphine oxide [diphenylphosphine oxide: 0.10 g, bis(4-methylphenyl)phosphine oxide: 0.11 g, bis(3,5-dimethylphenyl)phosphine oxide: 0.13 g], 0.49 mmol (0.063 mL) of diethyl phosphite, or 0.49 mmol (0.074 mL) of ethyl phenyl phosphinate, and 0.45 mmol (0.15 g) of cesium carbonate. Then, the vial was closed and irradiated in the MW reactor isothermally at 135–150 °C for 0.5–2.5 h (Table 2, entries 1, 3, 5, 8, and 12). The reaction mixture was diluted with 3 mL of EtOH, filtrated, and the residue obtained after evaporation of the filtrate was filtered through a thin (2–3 cm) layer of silica gel using ethyl acetate as the eluant. The crude mixture was analyzed by ^31^P NMR spectroscopy. Then, if the sample was relevant, it was purified further by column chromatography (silica gel, ethyl acetate–hexane 1:1 as the eluant) to afford products **1a**–**e**.

This procedure was repeated on a larger scale starting from 1.0 mmol (0.12 g) of phenylboronic acid, 1.2 mmol (0.24 g) of diphenylphosphine oxide, 1.1 mmol (0.36 g) of Cs_2_CO_3_, 0.10 mmol (0.022 g) of Pd(OAc)_2_ and 3.5 ml of ethanol. After an irradiation at 135 °C for 1.5 h, the work-up was similar as above to furnish 0.24 g (87%) of Ph_3_PO.

### Following Compounds Were Thus Prepared

#### Triphenylphosphine Oxide (**1a**) (Table 2, Entry 1)

Eluant: ethyl acetate–hexane 1:1; Yield: 0.087 g (83%) obtained as white crystals; mp. 156–157 °C, mp lit.^25^ 156.6–157.4 °C; ^31^P{^1^H} NMR (121.5 MHz, CDCl_3_) δ 29.1, δ_P_ lit.^25^ (162 MHz, CDCl_3_) 29.5; ^13^C{^1^H} NMR (75.5 MHz, CDCl_3_) δ 132.7 (d, *J* = 103.8 Hz), 132.2 (d, *J* = 9.9 Hz,), 132.0 (d, *J* = 2.8 Hz), 128.6 (d, *J* = 12.1 Hz), δ_C_ lit.^25^ (100 MHz, CDCl_3_) 132.8 (d, *J* = 104.6 Hz), 132.5 (d, *J* = 9.9 Hz), 131.9 (d, *J* = 2.2 Hz), 128.4 (d, *J* = 12.1 Hz); ^1^H NMR (300 MHz, CDCl_3_) δ 7.72–7.59 (m, 6H), 7.56–7.48 (m, 3H), 7.48–7.38 (m, 6H), δ_H_ lit.^25^ (400 MHz, CDCl_3_) 7.70–7.64 (m, 6H), 7.56–7.52 (m, 3H), 7.48–7.43 (m, 6H); HRMS (m/z): calcd for C_18_H_16_OP [M + H]^+^, 279.0939; found, 279.0941.

#### Bis(4-methylphenyl)phenylphosphine Oxide (**1b**) (Table 2, Entry 3)

Eluant: ethyl acetate–hexane 1:1; Yield: 0.090 g (78%) obtained as white crystals; mp. 76 °C, mp lit.^11^ 78–79 °C; ^31^P{^1^H} NMR (121.5 MHz, CDCl_3_) δ 27.8, δ_P_ lit.^26^ (162 MHz, CDCl_3_) 30.5; ^13^C{^1^H} NMR (75.5 MHz, CDCl_3_) δ 142.4 (d, *J* = 2.8 Hz), 133.1 (d, *J* = 104.1 Hz), 132.1 (d, *J* = 10.3 Hz), 132.1 (d, *J* = 9.8 Hz), 131.8 (d, *J* = 2.7 Hz), 129.4 (d, *J* = 106.6 Hz), 129.3 (d, *J* = 12.5 Hz), 128.5 (d, *J* = 12.1 Hz), 21.6 (s), δ_C_ lit.^26^ (100 MHz, CDCl_3_) 142.6 (d, *J* = 2.9 Hz), 133.0 (d, *J* = 102.5 Hz), 132.2 (d, *J* = 10.2 Hz), 132.0 (d, *J* = 8.7 Hz), 131.9 (d, *J* = 3.2 Hz), 129.4 (d, *J* = 106.9 Hz), 129.4 (d, *J* = 12.6 Hz), 128.6 (d, *J* = 11.8 Hz), 21.7; ^1^H NMR (300 MHz, CDCl_3_) δ 7.73–7.61 (m, 2H), 7.61–7.47 (m, 5H), 7.47–7.37 (m, 2H), 7.32–7.18 (m, 4H), 2.39 (s, 6H); δ_H_ lit^26^ (400 MHz, CDCl_3_) 7.68–7.62 (m, 2H), 7.53 (dd, *J*_1_ = 11.8 Hz, *J*_2_ = 8.0 Hz, 4H), 7.48 (m, 1H), 7.24 (dd, *J*_1_ = 8.4 Hz, *J*_2_ = 2.4 Hz, 4H), 2.38 (s, 6H); HRMS (*m*/*z*): calcd for C_20_H_20_OP [M + H]^+^, 307.1252; found, 307.1252.

#### Bis(3,5-dimethylphenyl)phenylphosphine Oxide (**1c**) (Table 2, Entry 5)

Eluant: ethyl acetate–hexane 1:1; Yield: 0.094 g (74%) obtained as white crystals; mp. 159 °C, mp. lit.^26^ 158.6–159.2 °C; ^31^P{^1^H} NMR (121.5 MHz, CDCl_3_): δ 29.6, δ_P_ lit.^26^ (162 MHz, CDCl_3_) 30.9; ^13^C{^1^H} NMR (75.5 MHz, CDCl_3_) δ 138.1 (d, *J* = 12.7 Hz), 133.7 (d, *J* = 2.8 Hz), 133.1 (d, *J* = 103.1 Hz), 132.4 (d, *J* = 105.3 Hz), 132.1 (d, *J* = 9.9 Hz), 131.7, 129.7 (d, *J* = 9.8 Hz), 128.4 (d, *J* = 12.0 Hz), 21.4 (s), δ_C_ lit.^26^ (100 MHz, CDCl_3_) 138.3 (d, *J* = 12.2 Hz), 133.9 (d, *J* = 2.3 Hz), 133.1 (d, *J* = 102.7 Hz), 132.4 (d, *J* = 102.6 Hz), 132.3 (d, *J* = 9.7 Hz), 131.9 (d, *J* = 2.2 Hz), 129.8 (d, *J* = 10.0 Hz), 128.6 (d, *J* = 11.7 Hz), 21.56; ^1^H NMR (300 MHz, CDCl_3_) δ 7.73–7.62 (m, 2H), 7.55–7.39 (m, 3H), 7.28 (d, *J* = 12.2 Hz, 4H), 7.15 (s, 2H), 2.31 (s, 12H), δ_H_ lit.^26^ (400 MHz, CDCl_3_) 7.68–7.63 (m, 2H), 7.55–7.51 (m, 1H), 7.47–7.42 (m, 2H), 7.26 (d, *J* = 12.4 Hz, 4H), 7.15 (s, 2H), 2.31 (s, 12H); HRMS (m/z): calcd for C_22_H_24_OP [M + H]^+^, 335.1565; found, 335.1566.

#### Diethyl Phenylphosphonate (**1d**) (Table 2, Entry 8)

Eluant: ethyl acetate–hexane 1:1; Yield: 0.068 g (84%) obtained as colorless oil; ^31^P{^1^H} NMR (121.5 MHz, CDCl_3_) δ 18.9, δ_P_ lit.^9^ (162 MHz, CDCl_3_) 18.8; ^13^C{^1^H} NMR (75.5 MHz, CDCl_3_) δ 132.5 (d, *J* = 3.0 Hz), 131.9 (d, *J* = 9.8 Hz), 128.6 (d, *J* = 15.0 Hz), 128.5 (d, *J* = 188.0 Hz), 62.2 (d, *J* = 5.4 Hz), 16.4 (d, *J* = 6.5 Hz), δ_C_ lit.^9^ (100 MHz, CDCl_3_) 132.3 (d, *J* = 2.7 Hz), 131.7 (d, *J* = 9.2 Hz), 128.44 (d, *J* = 15.2 Hz), 128.41 (d, *J* = 187.6 Hz), 62.0 (d, *J* = 5.9 Hz), 16.3 (d, *J* = 6.5 Hz); ^1^H NMR (300 MHz, CDCl_3_) δ 7.84–7.71 (m, 2H), 7.56–7.37 (m, 3H), 4.21–3.97 (m, 4H), 1.29 (t, *J* = 7.1 Hz, 6H), δ_H_ lit.^9^ (400 MHz, CDCl_3_) 7.82 (m, 2H), 7.55 (∼tq, *J*_1_ = 7.5 Hz, *J*_2_ = 1.4 Hz, 1H), 7.47 (m, 2H), 4.12 (m, 4H), 1.32 (td, *J*_1_*=* 7.0 Hz, *J*_2_ = 0.5 Hz, 6H); HRMS (*m*/*z*): calcd for C_10_H_16_O_3_P [M + H]^+^, 215.0837; found, 215.0835.

#### Diphenyl Ethylphosphinate (**1e**) (Table 2, Entry 12)

Eluant: ethyl acetate–hexane 1:1; Yield: 0.080 g (86%) obtained as colorless oil; ^31^P{^1^H} NMR (121.5 MHz, CDCl_3_) δ 32.2, δ_P_ lit.^27^ (120 MHz, CDCl_3_) 30.8; ^13^C{^1^H} NMR (75.5 MHz, CDCl_3_) δ 132.0 (d, *J* = 2.8 Hz), 131.7 (d, *J* = 137.0 Hz), 131.6 (d, *J* = 10.1 Hz), 128.4 (d, *J* = 13.1 Hz), 61.1 (d, *J* = 5.9 Hz), 16.5 (d, *J* = 6.6 Hz), δ_C_ lit.^27^ (75 MHz, CDCl_3_) 139.9 (d, *J* = 11.1 Hz), 133.7 (d, *J* = 143.4 Hz), 130.9 (d, *J* = 12.9 Hz), 127.0, 59.0, 16.7; ^1^H NMR (300 MHz, CDCl_3_) δ 7.88–7.75 (m, 4H), 7.58–7.39 (m, 6H), 4.16–4.01 (m, 2H), 1.36 (t, *J* = 7.1 Hz, 3H), δ_H_ lit.^27^ (300 MHz, CDCl_3_) 7.70–7.63 (m, 4H), 7.43–7.31 (m, 6H), 4.16–4.09 (m, 2H), 1.30 (t, *J* = 7.3 Hz, 3H); HRMS (m/z): calcd for C_14_H_16_O_2_P [M + H]^+^, 247.0888; found, 247.0889.

### General Procedure for the P–C Coupling of Arylboronic Acids and Diphenylphosphine Oxide or Diethyl Phosphite (Table 3, Entries 2–6 and **10** and Table 4, Entries 2–7)

To 0.038 mmol (0.0085 g) of Pd(OAc)_2_ in 1 mL of ethanol were added 0.41 mmol of the arlylboronic acid [4-, 2-, or 3-methylphenylboronic acid: 0.056 g, 4-fluorophenylboronic acid: 0.057 g, 4- or 3-chlorophenylboronic acid: 0.064 g], 0.49 mmol (0.10 g) of diphenylphosphine oxide or 0.49 mmol (0.063 mL) of diethyl phosphite, and 0.45 mmol (0.15 g) of cesium carbonate. Then, the resulting mixture was irradiated in a closed vial in the MW reactor isothermally at 135 or 150 °C for 1.5 or 0.5 h (Table 3, entries 2–6 and 10 and Table 4). The reaction mixture was diluted with 3 mL of EtOH, filtrated, and the residue obtained after evaporation of the filtrate was filtered through a thin (2–3 cm) layer of silica gel using ethyl acetate as the eluant. The crude mixture obtained was analyzed by ^31^P NMR spectroscopy. Then, if the sample was relevant, it was purified further by column chromatography (silica gel, ethyl acetate–hexane 1:1 as the eluant) to afford products **2f**–**4k**.

### Following Compounds Were Thus Prepared

#### (4-Methylphenyl)diphenylphosphine Oxide (**2f**) (Table 3, Entry 2)

Eluant: ethyl acetate–hexane 1:1; Yield: 0.081 g (73%) obtained as a white solid; mp 118–119 °C, mp. lit.^13^ 129.5–130.2 °C; ^31^P{^1^H} NMR (121.5 MHz, CDCl_3_) δ 29.3, δ_P_ lit.^13^ (162 MHz, CDCl_3_) 29.1; ^13^C{^1^H} NMR (125.7 MHz, CDCl_3_) δ 142.4 (d, *J* = 2.8 Hz), 132.8 (d, *J* = 105.9 Hz), 132.1 (d, *J* = 10.2 Hz), 132.0 (d, *J* = 9.9 Hz), 131.8 (d, *J* = 2.7 Hz), 129.2 (d, *J* = 12.6 Hz), 129.1 (d, *J* = 106.4 Hz), 128.4 (d, *J* = 12.1 Hz), 21.6 (s), δ_C_ lit.^13^ (100 MHz, CDCl_3_) 142.5 (d, *J* = 2.6 Hz), 132.9 (d, *J* = 104.1 Hz), 132.2 (d, *J* = 10.2 Hz), 132.1 (d, *J* = 10.0 Hz), 131.9 (d, *J* = 2.8 Hz), 129.3 (d, *J* = 12.5 Hz), 129.2 (d, *J* = 106.4 Hz), 128.5 (d, *J* = 11.9 Hz), 21.6; ^1^H NMR (500 MHz, CDCl_3_) δ 7.80–7.40 (m, 12H), 7.29–7.11 (m, 2H), 2.40 (s, 3H), δ_H_ lit.^13^ (400 MHz, CDCl_3_) 7.67–7.62 (m, 4H), 7.56–7.48 (m, 4H), 7.44–7.40 (m, 4H), 7.26–7.23 (m, 2H), 2.37 (s, 3H); HRMS (m/z): calcd for C_19_H_18_OP [M + H]^+^, 293.1095; found, 293.1101.

#### (2-Methylphenyl)diphenylphosphine Oxide (**2g**) (Table 3, Entry 3)

Eluant: ethyl acetate–hexane 1:1; Yield: 0.074 g (67%) obtained as white crystals; mp. 120–121 °C, mp. lit.^28^ 121.5–122.9 °C; ^31^P{^1^H} NMR (202.4 MHz, CDCl_3_) δ 31.8, δ_P_ lit.^28^ (202 MHz, CDCl_3_) 31.8; ^13^C{^1^H} NMR (125.7 MHz, CDCl_3_) δ 143.4 (d, *J* = 8.0 Hz), 133.5 (d, *J* = 12.9 Hz), 132.8 (d, *J* = 103.6 Hz), 132.1 (d, *J* = 2.6 Hz), 132.0 (d), 131.8 (d, *J* = 2.7 Hz), 130.8 (d, *J* = 103.2 Hz), 128.6 (d, *J* = 12.0 Hz), 125.2 (d, *J* = 12.8 Hz), 21.7 (d, *J* = 4.7 Hz), δ_C_ lit.^28^ (125 MHz, CDCl_3_) 143.2 (d, *J* = 8.2 Hz), 133.4 (d, *J* = 12.4 Hz), 132.6 (d, *J* = 102.6 Hz), 132.0 (d, *J* = 2.7 Hz), 131.8 (d, *J* = 10.0 Hz), 131.7 (d, *J* = 2.7 Hz), 130.7 (d, *J* = 103.5 Hz), 128.5 (d, *J* = 12.7 Hz), 125.1 (d, *J* = 12.7 Hz), 21.6 (d, *J* = 5.4 Hz); ^1^H NMR (500 MHz, CDCl_3_) δ 7.66 (m, 4H), 7.55 (tq, *J*_1_ = 7.3 Hz, *J*_2_ = 1.6 Hz, 2H), 7.47 (td, *J*_1_ = 7.7 Hz, *J*_2_ = 2.8 Hz, 4H), 7.42 (tt, *J*_1_ = 7.6 Hz, *J*_2_ = 1.6 Hz, 1H), 7.28 (dd, *J*_1_ = 7.6 Hz, *J*_2_ = 4.1 Hz, 1H), 7.13 (td, *J*_1_ = 7.6 Hz, *J*_2_ = 2.9 Hz, 1H), 7.03 (ddd, *J*_1_ = 14.1 Hz, *J*_2_ = 7.8 Hz, *J*_3_ = 1.1 Hz, 1H), 2.45 (s, 3H), δ_H_ lit.^28^ (500 MHz, CDCl_3_) 7.68–7.62 (m, 4H), 7.57–7.52 (m, 2H), 7.49–7.45 (m, 4H), 7.44–7.39 (m, 1H), 7.30–7.26 (m, 1H), 7.15–7.10 (m, 1H), 7.05–7.00 (m, 1H), 2.45 (s, 3H); HRMS (*m*/*z*): calcd for C_19_H_18_OP [M + H]^+^, 293.1095; found, 293.1094.

#### (3-Methylphenyl)diphenylphosphine Oxide (**2h**) (Table 3, Entry 4)

Eluant: ethyl acetate–hexane 1:1; Yield: 0.089 g (80%) obtained as a white solid; mp. 122–123 °C, mp. lit.^26^ 123.7–124.2 °C; ^31^P{^1^H} NMR (202.4 MHz, CDCl_3_) δ 29.3, δ_P_ lit.^29^ (162 MHz, CDCl_3_) 29.5; ^13^C{^1^H} NMR (125.7 MHz, CDCl_3_) δ 138.5 (d, *J* = 12.1 Hz), 132.8 (d, *J* = 3.0 Hz), 132.7 (d, *J* = 104.0 Hz), 132.5 (d, *J* = 9.5 Hz), 132.3 (d, *J* = 96.7 Hz), 132.1 (d, *J* = 9.9 Hz), 131.9 (d, *J* = 2.6 Hz), 129.2 (d, *J* = 10.2 Hz), 128.5 (d, *J* = 12.0 Hz), 128.3 (d, *J* = 12.9 Hz), 21.4 (s), δ_C_ lit.^29^ (100 MHz, CDCl_3_) 138.4 (d, *J* = 15.9 Hz), 133.1, 132.8 (d, *J* = 2.4 Hz), 132.5 (d, *J* = 9.5 Hz), 132.2 (d, *J* = 103.4 Hz), 132.0 (d, *J* = 9.8 Hz), 131.8 (d, *J* = 2.5 Hz), 129.2 (d, *J* = 10.2 Hz), 128.5 (d, *J* = 12.1 Hz), 128.2, 21.5; ^1^H NMR (500 MHz, CDCl_3_) δ 7.72–7.67 (m, 4H), 7.61–7.55 (m, 3H), 7.50–7.47 (m, 4H), 7.42–7.34 (m, 3H), 2.39 (s, 3H), δ_H_ lit.^29^ (400 MHz, CDCl_3_) 7.64 (dd, *J*_1_ = 11.6 Hz, *J*_*2*_ = 7.6 Hz, 4H), 7.56–7.49 (m, 3H), 7.44–7.43 (m, 4H), 7.32 (m, 3H), 2.33 (s, 3H); HRMS (*m*/*z*): calcd for C_19_H_18_OP [M + H]^+^, 293.1095; found, 293.1097.

#### (4-Fluorophenyl)diphenylphosphine Oxide (**2i**) (Table 3, Entry 5)

Eluant: ethyl acetate–hexane 1:1; Yield: 0.079 g (70%) obtained as pale yellow crystals; mp. 134–135 °C, mp.^30^ 134–135 °C; ^31^P{^1^H} NMR (121.5 MHz, CDCl_3_) δ 28.5, δ_P_ lit.^23^ (162 MHz, CDCl_3_) 28.3; ^13^C{^1^H} NMR (125.7 MHz, CDCl_3_) δ 165.0 (dd, *J*_1_ = 3.2 Hz, *J*_2_ = 253.6 Hz), 134.5 (dd, *J*_1_ = 11.3 Hz, *J*_2_ = 8.8 Hz), 132.3 (d, *J* = 105.0 Hz), ∼132.1 (d, *J* ∼ 3.0 Hz), 132.0 (d, *J* = 12.2 Hz), 128.53 (d, *J* = 12.2 Hz), 128.52 (dd, *J*_1_ = 106.5 Hz, *J*_2_ = 3.4 Hz), 115.8 (dd, *J*_1_ = 13.2 Hz, *J*_2_ = 21.4 Hz), δ_C_ lit.^23^ (100 MHz, CDCl_3_) 165.0 (dd, *J*_1_ = 254.2 Hz, *J*_2_ = 3.2 Hz), 134.6 (dd, *J*_1_ = 11.3 Hz, *J*_2_ = 8.9 Hz), 132.5 (d, *J* = 105.2 Hz), 132.1 (d, *J* = 4.1 Hz), 132.0, 128.7 (dd, *J*_1_ = 106.6 Hz, *J*_2_ = 3.1 Hz), 128.6 (d, *J* = 12.5 Hz), 115.9 (dd, *J*_1_ = 22.6 Hz, *J*_2_ = 13.3 Hz); ^1^H NMR (500 MHz, CDCl_3_) δ 7.75–7.37 (m, 12H), 7.20–7.06 (m, 2H), δ_H_ lit.^23^ (400 MHz, CDCl_3_) 7.67–7.60 (m, 6H), 7.53–7.49 (m, 2H), 7.44–7.41 (m, 4H), 7.11 (t, *J* = 8.5 Hz, 2H); HRMS (*m*/*z*): calcd for C_18_H_15_FOP [M + H]^+^, 297.0845; found, 297.0850.

#### 1,4-Phenylenebis(diphenylphosphine oxide) (**3A**) (Table 3, Entry 6)

In this case, dichloromethane–methanol 97:3 was used as the eluant. Yield: 0.021 g (23%) obtained as white crystals; mp. 300–301 °C, mp. lit.^25^ >300 °C; ^31^P{^1^H} NMR (202.4 MHz, CDCl_3_) δ 28.6, δ_P_ lit.^25^ (162 MHz, CDCl_3_) 30.5; ^13^C{^1^H} NMR (125.7 MHz, CDCl_3_) δ 137.0 (dd, *J*_1_ = 100.6 Hz, *J*_2_ = 2.7 Hz), 132.3, 132.06 (d, *J* = 10.1 Hz), 132.06, 131.7 (d, *J* = 105.2 Hz), 128.7 (d, *J*_1_ = 12.8 Hz), δ_C_ lit.^25^ (100 MHz, CDCl_3_) 136.9 (d, *J* = 100.2 Hz), 132.3, 132.0 (d, *J* = 13.9 Hz), 131.9, 131.0, 128.7 (d, *J* = 12.2 Hz); ^1^H NMR (500 MHz, CDCl_3_) δ 7.76 (m, 4H), 7.66 (ddd, *J*_1_ = 12.1 Hz, *J*_2_ = 8.4 Hz, *J*_3_ = 1.3 Hz, 8H), 7.57 (tq, *J*_1_ = 7.4 Hz, *J*_2_ = 1.5 Hz, 4H), 7.48 (td, *J*_1_ = 7.8 Hz, *J*_2_ = 2.8 Hz, 8H), δ_H_ lit.^25^ (400 MHz, CDCl_3_) 7.77–7.74 (m, 4H), 7.69–7.64 (m, 8H), 7.58–7.55 (m, 4H), 7.50–7.46 (m, 8H); HRMS (*m*/*z*): calcd for C_30_H_25_O_2_P_2_ [M + H]^+^, 479.1330; found, 479.1320.

#### 1,3-Phenylenebis(diphenylphosphine oxide) (**3B**) (Table 3, Entry 10)

In this instance, dichloromethane–methanol 97:3 was used as the eluant. Yield: 0.023 g (25%) obtained as colorless oil; ^31^P{^1^H} NMR (202.4 MHz, CDCl_3_) δ 28.5, δ_P_ lit.^25^ (162 MHz, CDCl_3_) 30.5; ^13^C{^1^H} NMR (125.7 MHz, CDCl_3_) δ 135.5 (dd, *J*_1_ = 10.1 Hz, *J*_2_ = 3.1 Hz), 135.4 (t, *J* = 11.2 Hz), 133.6 (dd, *J*_*1*_ = 101.8 Hz, *J*_2_ = 10.7 Hz), 132.2 (d, *J* = 2.3 Hz), 132.0 (d, *J* = 10.2 Hz), 131.7 (d, *J* = 105.1 Hz), 129.0 (t, *J* = 11.3 Hz), 128.6 (d, *J* = 12.5 Hz), δ_C_ lit.^25^ (100 MHz, CDCl_3_) 135.4–135.2 (m, 2C), 135.1, 133.5 (dd, *J*_1_ = 101.7 Hz, *J*_2_ = 10.7 Hz), 132.0, 131.8 (d, *J* = 10.3 Hz), 131.5 (d, *J* = 104.9 Hz), 128.8 (t, *J* = 11.2 Hz), 128.4 (d, *J* = 12.6 Hz), 127.1; ^1^H NMR (500 MHz, CDCl_3_) δ 7.96 (ddm, *J*_1_ = 12.5 Hz, *J*_2_ = 7.7 Hz, 2H), 7.69 (tt, *J*_1_ = 11.7 Hz, *J*_2_ = 1.5 Hz, 1H), 7.62 (tt, *J*_1_ = 7.7 Hz, *J*_2_ = 2.5 Hz, 1H), 7.58 (dd, *J*_1_ = 12.1 Hz, *J*_2_ = 7.9 Hz, 8H), 7.53 (t, *J* = 7.4 Hz, 4H), 7.41 (td, *J*_1_ = 7.7 Hz, *J*_2_ = 2.8 Hz, 8H), δ_H_ lit.^25^ (400 MHz, CDCl_3_) 7.98–7.93 (m, 2H), 7.71 (t, *J* = 11.7 Hz, 1H), 7.63–7.50 (m, 13H), 7.43–7.38 (m, 8H); HRMS (m/z): calcd for C_30_H_25_O_2_P_2_ [M + H]^+^, 479.1330; found, 479.1323.

#### Diethyl (4-Methylphenyl)phosphonate (**4f**) (Table 4, Entry 2)

Eluant: ethyl acetate–hexane 1:1; Yield: 0.067 g (77%) obtained as colorless oil; ^31^P{^1^H} NMR (121.5 MHz, CDCl_3_) δ 20.5, δ_P_ lit.^17^ (162 MHz, CDCl_3_) 19.5; ^13^C{^1^H} NMR (75.5 MHz, CDCl_3_) δ 142.8 (d, *J* = 3.1 Hz), 131.7 (d, *J* = 10.3 Hz), 129.1 (d, *J* = 15.4 Hz), 125.0 (d, *J* = 190.1 Hz), 61.9 (d, *J* = 5.3 Hz), 21.6 (d, *J* = 1.15 Hz), 16.2 (d, *J* = 6.5 Hz), δ_C_ lit.^17^ (100 MHz, CDCl_3_) 142.9 (d, *J* = 3.3 Hz), 131.8 (d, *J* = 10.4 Hz), 129.3 (d, *J* = 15.4 Hz), 125.0 (d, *J* = 190.2 Hz), 61.9 (d, *J* = 5.3 Hz), 21.6, 16.3 (d, *J* = 6.3 Hz); ^1^H NMR (300 MHz, CDCl_3_) δ 7.75–7.63 (m, 2H), 7.25–7.20 (m, 2H), 4.19–3.94 (m, 4H), 2.37 (s, 3H), 1.28 (t, *J* = 7.1 Hz, 6H), δ_H_ lit.^17^ (400 MHz, CDCl_3_) 7.63 (dd, *J*_1_ = 12.9 Hz, *J*_2_ = 7.8 Hz, 2H), 7.24–7.16 (m, 2H), 4.12–3.93 (m, 4H), 2.33 (s, 3H), 1.24 (t, *J* = 6.9 Hz, 6H); HRMS (*m*/*z*): calcd for C_11_H_18_O_3_P [M + H]^+^, 229.0994 found 229.0996.

#### Diethyl (2-Methylphenyl)phosphonate (**4g**) (Table 4, Entry 3)

Eluant: ethyl acetate–hexane 1:1; Yield: 0.067 g (78%) obtained as colorless oil; ^31^P{^1^H} NMR (202.4 MHz, CDCl_3_) δ 19.5, δ_P_ lit.^17^ (162 MHz, CDCl_3_) 19.4; ^13^C{^1^H} NMR (125.7 MHz, CDCl_3_) δ 141.8 (d, *J* = 10.1 Hz), 133.9 (d, *J* = 10.4 Hz), 132.4 (d, *J* = 2.8 Hz), 131.2 (d, *J* = 15.0 Hz), 126.8 (d, *J* = 184.0 Hz), 125.4 (d, *J* = 14.8 Hz), 61.9 (d, *J* = 5.5 Hz), 21.2 (d, *J* = 3.7 Hz), 16.3 (d, *J* = 6.6 Hz), δ_C_ lit.^17^ (100 MHz, CDCl_3_) 141.8 (d, *J* = 10.4 Hz), 133.9 (d, *J* = 10.3 Hz), 132.4 (d, *J* = 2.9 Hz), 131.1 (d, *J* = 15.5 Hz), 127.0 (d, *J* = 184.4 Hz), 125.3 (d, *J* = 14.9 Hz), 61.8 (d, *J* = 5.6 Hz), 21.2 (d, *J* = 3.7 Hz), 16.3 (d, *J* = 6.6 Hz); ^1^H NMR (500 MHz, CDCl_3_) δ 7.96–7.90 (m, 1H), 7.47–7.43 (m, 1H), 7.32–7.25 (m, 2H), 4.24–4.05 (m, 4H), 2.60 (s, 3H), 1.35 (t, *J* = 7.1 Hz, 6H), δ_H_ lit.^17^ (400 MHz, CDCl_3_) 7.95–7.88 (m, 1H), 7.45–7.40 (m, 1H), 7.29–7.23 (m, 2H), 4.20–4.03 (m, 4H), 2.58 (s, 3H), 1.33 (t, *J* = 7.0 Hz, 6H); HRMS (*m*/*z*): calcd for C_11_H_18_O_3_P [M + H]^+^, 229.0994; found, 229.0993.

#### Diethyl (3-Methylphenyl)phosphonate (**4h**) (Table 4, Entry 4)

Eluant: ethyl acetate–hexane 1:1; Yield: 0.069 g (80%) obtained as colorless oil; ^31^P{^1^H} NMR (202.4 MHz, CDCl_3_) δ 19.5, δ_P_ lit.^17^ (162 MHz, CDCl_3_) 19.2; ^13^C{^1^H} NMR (125.7 MHz, CDCl_3_) δ 138.3 (d, *J* = 14.9 Hz), 133.2 (d, *J* = 3.1 Hz), 132.3 (d, *J* = 10.0 Hz), 128.8 (d, *J* = 9.6 Hz), 128.4 (d, *J* = 15.8 Hz), 128.1 (d, *J* = 187.6 Hz), 62.1 (d, *J* = 5.4 Hz), 21.3 (s), 16.3 (d, *J* = 6.5 Hz), δ_C_ lit.^17^ (100 MHz, CDCl_3_) 138.2 (d, *J* = 14.8 Hz), 133.1 (d, *J* = 3.0 Hz), 132.2 (d, *J* = 9.7 Hz), 128.7 (d, *J* = 9.7 Hz), 128.3 (d, *J* = 15.4 Hz), 128.0 (d, *J* = 187.0 Hz), 61.9 (d, *J* = 5.2 Hz), 21.2, 16.3 (d, *J* = 6.2 Hz); ^1^H NMR (500 MHz, CDCl_3_) δ 7.67–7.57 (m, 2H), 7.38–7.34 (m, 2H), 4.19–4.03 (m, 4H), 2.40 (s, 3H), 1.33 (t, *J* = 7.1 Hz, 6H), δ_H_ lit.^17^ (400 MHz, CDCl_3_) 7.63–7.52 (m, 2H), 7.33–7.27 (m, 2H), 4.16–3.97 (m, 4H), 2.34 (s, 3H), 1.27 (t, *J* = 7.1 Hz, 6H); HRMS (*m*/*z*): calcd for C_11_H_18_O_3_P [M + H]^+^, 229.0994; found, 229.0992.

#### Diethyl (4-Fluorophenyl)phosphonate (**4i**) (Table 4, Entry 5)

Eluant: ethyl acetate–hexane 1:1; Yield: 0.068 g (77%) obtained as colorless oil; ^31^P{^1^H} NMR (121.5 MHz, CDCl_3_) δ 18.7, δ_P_ lit.^17^ (162 MHz, CDCl_3_) 17.7; ^13^C{^1^H} NMR (75.5 MHz, CDCl_3_) δ 165.2 (dd, *J*_1_ = 3.9 Hz, *J*_2_ = 253.4 Hz), 134.2 (dd, *J*_1_ = 11.2 Hz, *J*_2_ = 8.9 Hz), 124.4 (dd, *J*_1_ = 192.7 Hz, *J*_2_ = 3.4 Hz), 115.6 (dd, *J*_1_ = 16.3 Hz, *J*_2_ = 21.4 Hz), 62.0 (d, *J* = 5.4 Hz), 16.2 (d, *J* = 6.4 Hz), δ_C_ lit.^17^ (100 MHz, CDCl_3_) 165.3 (dd, *J*_1_ = 253.1 Hz, *J*_2_ = 4.1 Hz), 134.5 (dd, *J*_1_ = 11.2 Hz, *J*_2_ = 8.8 Hz), 124.4 (d, *J* = 192.7 Hz), 115.8 (dd, *J*_1_ = 22.1 Hz, *J*_2_ = 16.2 Hz), 62.1 (d, *J* = 5.4 Hz), 16.3 (d, *J* = 6.2 Hz); ^1^H NMR (300 MHz, CDCl_3_) δ 7.81–7.70 (m, 2H), 7.11–7.00 (m, 2H), 4.15–3.93 (m, 4H), 1.32–1.15 (m, 6H), δ_H_ lit.^17^ (400 MHz, CDCl_3_) 7.80 (ddd, *J*_1_ = 14.0 Hz, *J*_2_ = 7.4 Hz, *J*_3_ = 6.9 Hz, 2H), 7.17–7.09 (m, 2H), 4.19–4.00 (m, 4H), 1.30 (t, *J* = 7.2 Hz, 6H); HRMS (*m*/*z*): calcd for C_10_H_15_FO_3_P [M + H]^+^, 233.0743; found, 233.0742.

#### Diethyl (4-Chlorophenyl)phosphonate (**4j**) (Table 4, Entry 6)

Eluant: ethyl acetate–hexane 1:1; Yield: 0.062 g (66%) obtained as colorless oil; ^31^P{^1^H} NMR (121.5 MHz, CDCl_3_) δ 18.5, δ_P_ lit.^31^ (162 MHz, CDCl_3_) 18.1; ^13^C{^1^H} NMR (75.5 MHz, CDCl_3_) δ 139.0 (d, *J* = 4.1 Hz), 133.2 (d, *J* = 10.7 Hz), 128.9 (d, *J* = 15.6 Hz), 127.1 (d, *J* = 190.9 Hz), 62.3 (d, *J* = 5.5 Hz), 16.4 (d, *J* = 6.4 Hz), δ_C_ lit.^31^ (62.5 MHz, CDCl_3_) 138.5, 134.5, 129.2, 123.3, 58.0, 16.8; ^1^H NMR (300 MHz, CDCl_3_) δ 7.81–7.65 (m, 2H), 7.51–7.40 (m, 2H), 4.21–4.00 (m, 4H), 1.32 (t, *J* = 7.0 Hz, 6H), δ_H_ lit.^31^ (250 MHz, CDCl_3_) 7.69–7.61 (m, 2H), 7.38–7.33 (m, 2H), 4.04–3.95 (m, 4H), 1.22 (t, 6H); HRMS (*m*/*z*): calcd for C_10_H_15_ClO_3_P [M + H]^+^, 249.0447; found, 249.0446.

#### Diethyl (3-Chlorophenyl)phosphonate (**4k**) (Table 4, Entry 7)

Eluant: ethyl acetate–hexane 1:1; Yield: 0.067 g (71%) obtained as colorless oil; ^31^P{^1^H} NMR (121.5 MHz, CDCl_3_) δ 17.4, δ_P_ lit.^32^ (202.4 MHz, CDCl_3_) 16.7; ^13^C{^1^H} NMR (125.7 MHz, CDCl_3_) δ 134.7 (d, *J* = 20.3 Hz), 132.4 (d, *J* = 3.0 Hz), 131.6 (d, *J* = 10.7 Hz), 130.7 (d, *J* = 187.9 Hz), 129.74 (d, *J* = 16.3 Hz), 129.69 (d, *J* = 9.2 Hz), 62.3 (d, *J* = 5.5 Hz), 16.2 (d, *J* = 6.4 Hz), δ_C_ lit.^32^ (125.7 MHz, CDCl_3_) 134.8 (d, *J* = 20.3 Hz), 132.5 (d, *J* = 3.0 Hz), 131.6 (d, *J* = 10.7 Hz), 130.7 (d, *J* = 188.2 Hz), 130.0 (d, *J* = 16.2 Hz), 129.8 (d, *J* = 9.1 Hz), 62.5 (d, *J* = 5.5 Hz), 16.3 (d, *J* = 6.4 Hz); ^1^H NMR (500 MHz, CDCl_3_) δ 7.81–7.59 (m, 2H), 7.51–7.43 (m, 1H), 7.40–7.31 (m, 1H), 4.20–3.96 (m, 4H), 1.30 (t, *J* = 7.1 Hz, 6H), δ_H_ lit.^32^ (500 MHz, CDCl_3_) 7.78–7.37 (m, 4H), 4.18–4.02 (m, 4H), 1.31 (t, *J* = 7.0 Hz, 6H); HRMS (*m*/*z*): calcd for C_10_H_15_ClO_3_P [M + H]^+^, 249.0447; found, 249.0448.

### Computational Methods

All computations were carried out with the Gaussian16 program package (G16),^33^ using standard convergence criteria for the gradients of the root-mean-square (RMS) force, maximum force, and RMS displacement and maximum displacement vectors (3.0 × 10^–4^, 4.5 × 10^–4^, 1.2 × 10^–3^, and 1.8 × 10^–3^). Computations were carried out at the M06-2X level of theory.^34^ The basis set of 6-31G(d,p) was applied for C, H, O, P, N, and B and SDD/MWB28 for Pd.^35^ The vibrational frequencies were computed at the same levels of theory, in order to confirm properly all structures as residing at minima on their potential energy hypersurfaces (PESs). Thermodynamic functions U, H, G, and S were computed at 398.15 K. Besides the vacuum calculations, the IEFPCM method was also applied to model the solvent effect, by using the default settings of G16, setting the ε = 24.852 (for ethanol).^36^ See the Supporting Information for details.

## Data Availability

The data underlying this study are available in the published article and its Supporting Information.

## Supporting Information Available

^31^P, ^13^C, and ^1^H NMR spectra and data of the theoretical calculations (PDF)
