# Supplementary material for: Microwave-Assisted Palladium Acetate-Catalyzed C–P Cross-Coupling of Arylboronic Acids and >P(O)H Reagents in the Absence of the Usual Mono- and Bidentate P-Ligands: Mechanistic Insights
Source: J Org Chem. 2023 Aug 9;88(16):11980–91. doi: 10.1021/acs.joc.3c01269 (PMC10442920; doi:10.1021/acs.joc.3c01269)
Supplement: Supplementary file 1 — jo3c01269_si_001.pdf [file jo3c01269_si_001.pdf]

Supporting Information

Microwave-Assisted Palladium-Acetate Catalyzed C–P Cross Coupling of Arylboronic Acids and >P(O)H Reagents in the Absence of the Usual Mono- and Bidental P-Ligands; Mechanistic Insights

Bianka Huszár,[a] Zoltán Mucsi[a,b] and György Keglevich[a*]

[a]Department of Organic Chemistry and Technology, Faculty of Chemical Technology and Biotechnology, Budapest University of Technology and Economics, 1521 Budapest, Hungary

keglevich.gyorgy@vbk.bme.hu

[b]Faculty of Materials and Chemical Sciences, University of Miskolc, Miskolc H-3515, Hungary

Table of contents

1. NMR spectra for compounds **1a–4k** pg. S2–S52.
2. Table S1–S3 containing the computed row data pg. S53–S54.
3. Additional Tables containing XYZ coordinates of computed species pg. S55–S90.

$^{31}P\{^{1}H\}$ NMR (121.5 MHz, $CDCl_3$)

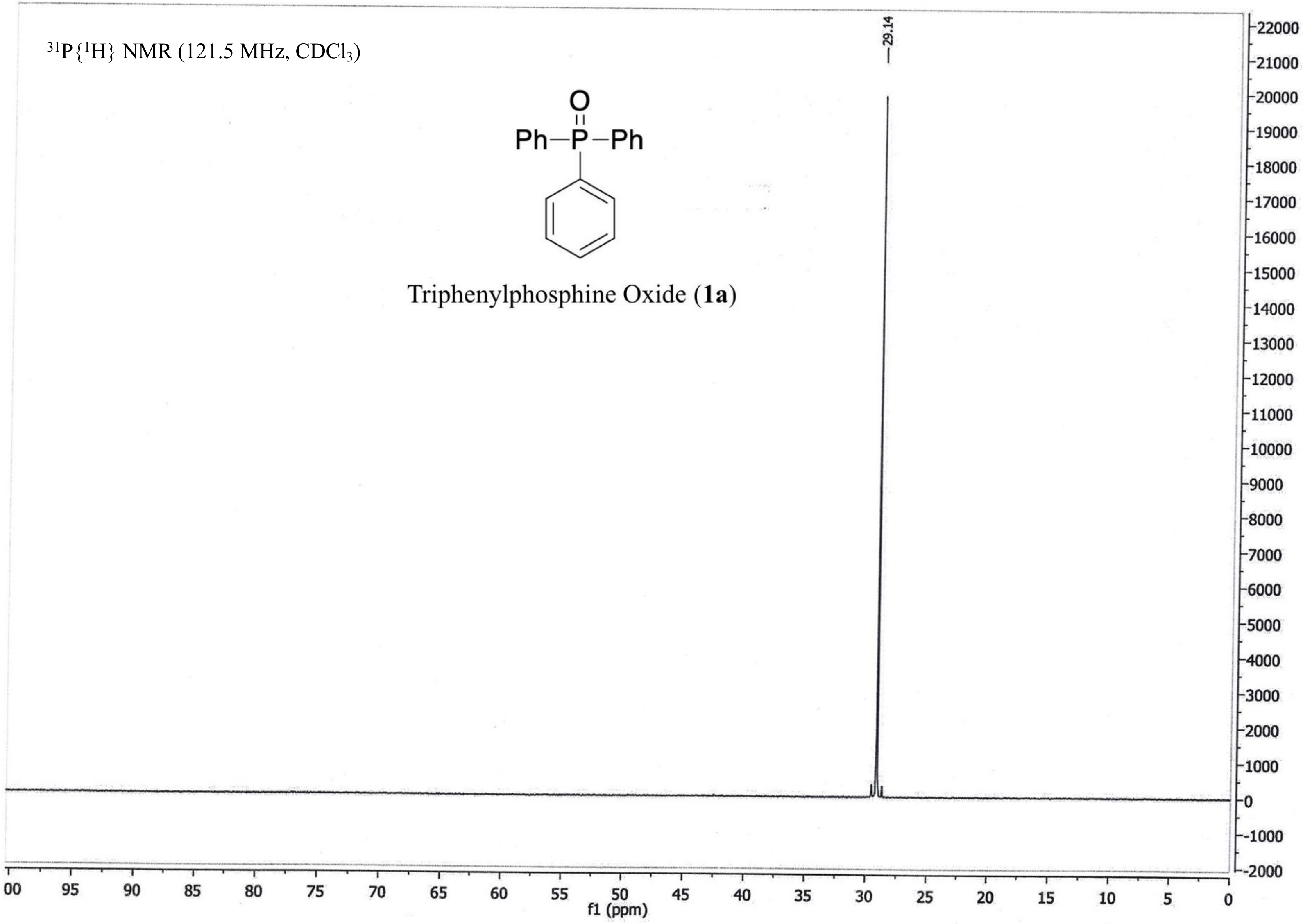

Triphenylphosphine Oxide (**1a**)

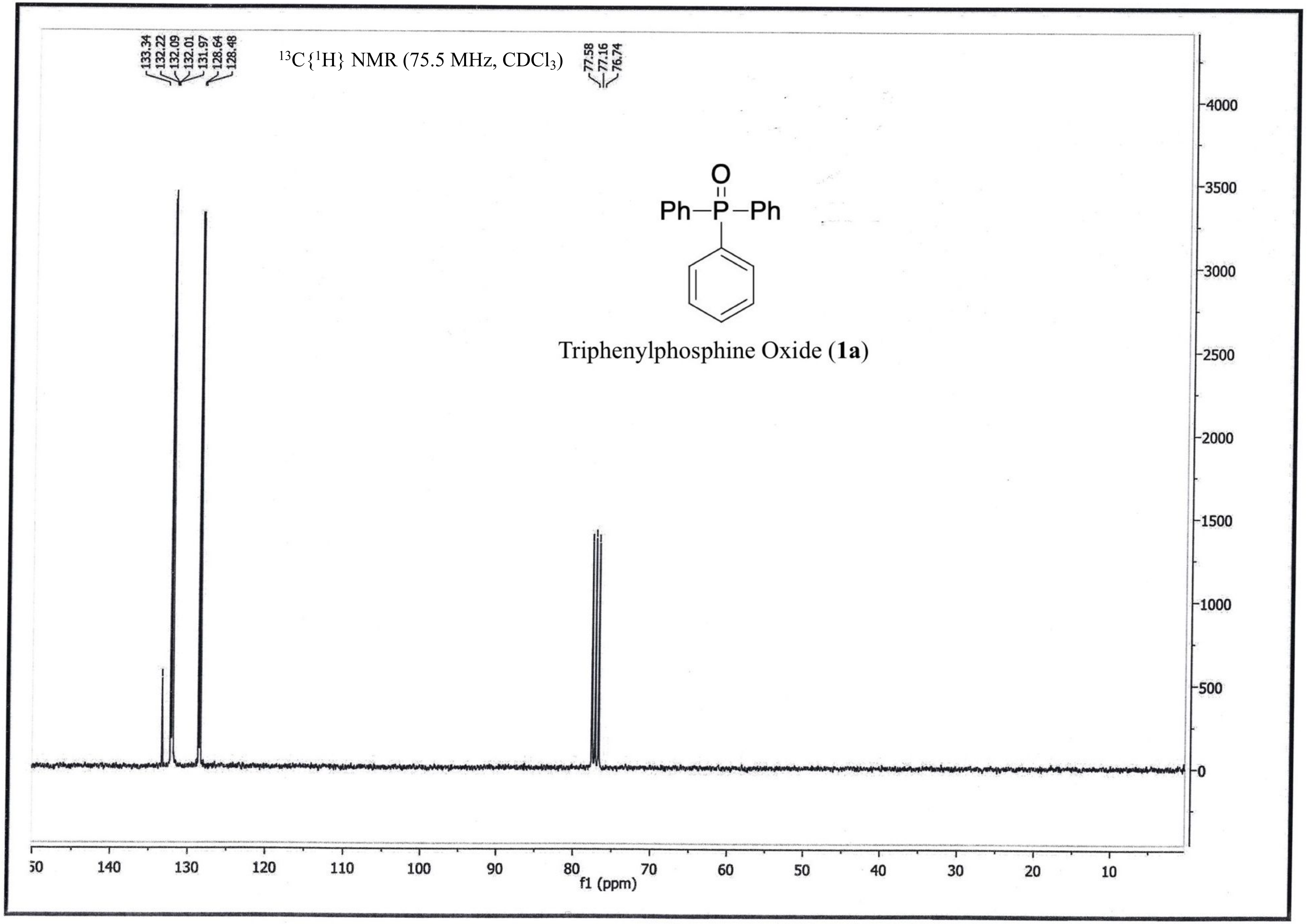
133.34
132.22
132.09
132.01
131.97
128.64
128.48
$^{13}C\{^1H\}$ NMR (75.5 MHz, CDCl$_3$)
77.58
77.16
76.74
O
Ph—P—Ph
Triphenylphosphine Oxide (**1a**)
4000
3500
3000
2500
2000
1500
1000
500
0
140
130
120
110
100
90
80
70
60
50
40
30
20
10
f1 (ppm)

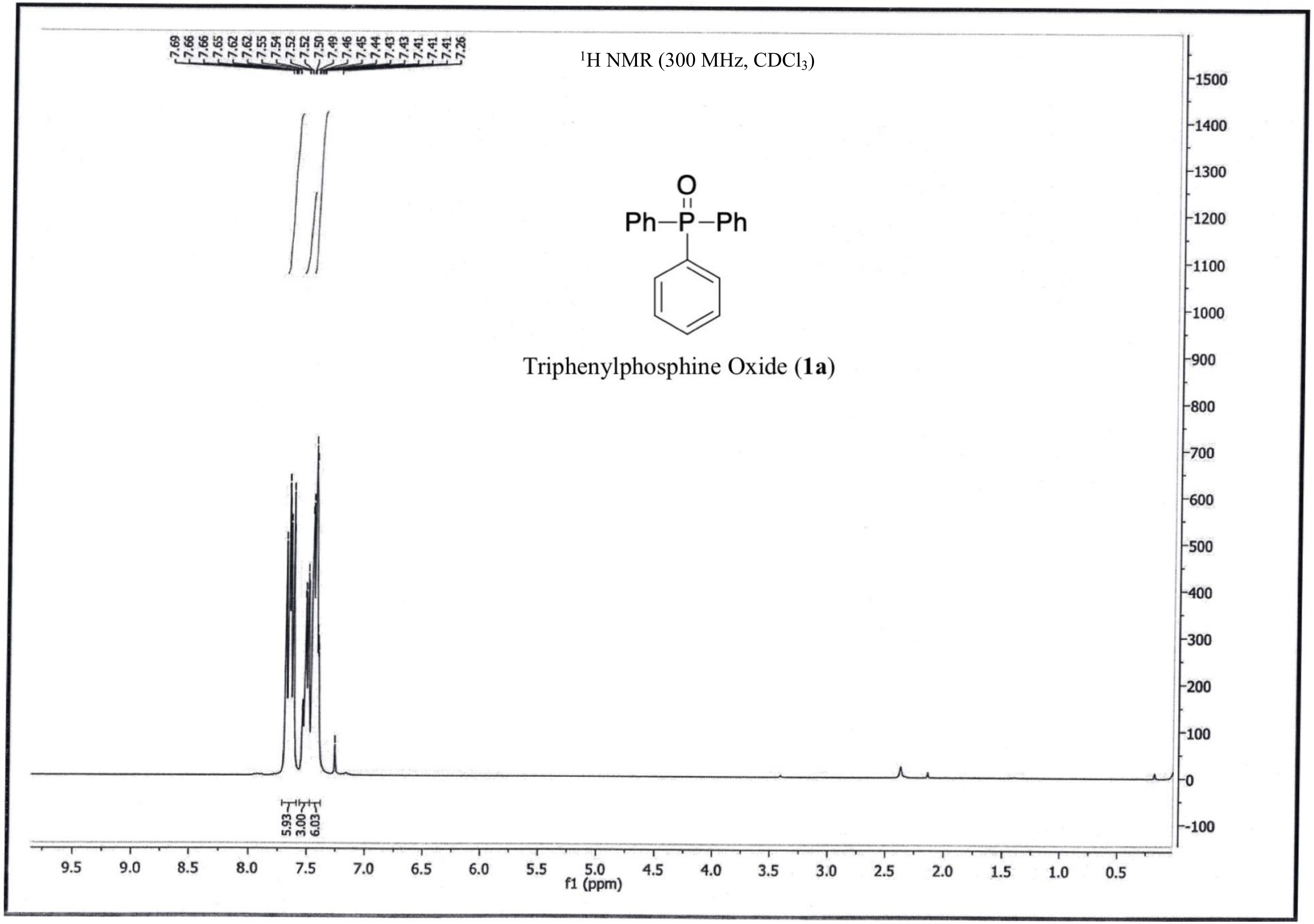

Triphenylphosphine Oxide (**1a**)

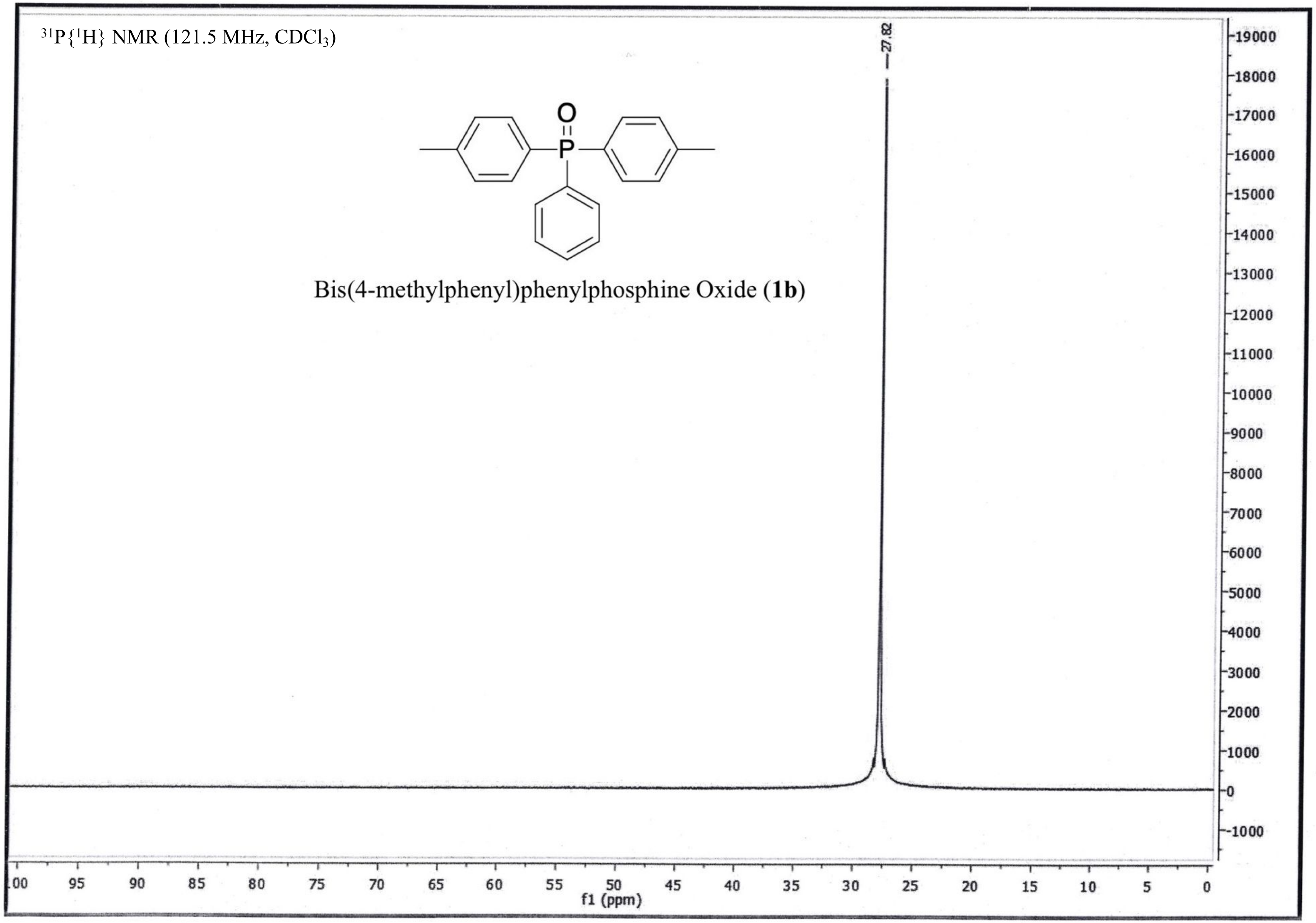

$^{31}P\{^1H\}$ NMR (121.5 MHz, $CDCl_3$)
O
P
Bis(4-methylphenyl)phenylphosphine Oxide (**1b**)
27.82
19000
18000
17000
16000
15000
14000
13000
12000
11000
10000
9000
8000
7000
6000
5000
4000
3000
2000
1000
0
-1000
100 95 90 85 80 75 70 65 60 55 50 45 40 35 30 25 20 15 10 5 0
f1 (ppm)

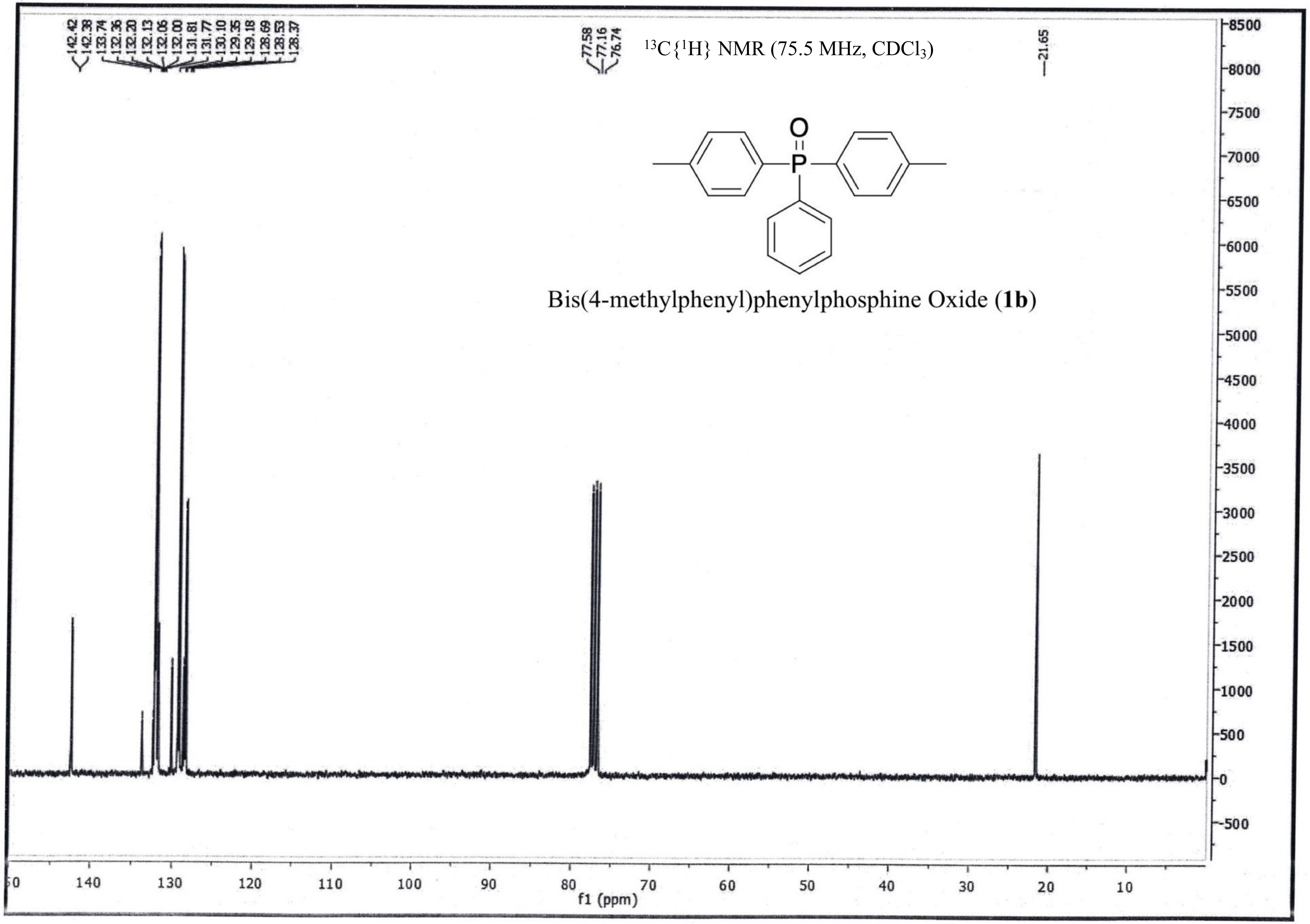
142.42
142.38
133.74
132.36
132.20
132.13
132.06
132.00
131.81
131.77
130.10
129.35
129.18
128.69
128.53
128.37
77.58
77.16
76.74
$^{13}C\{^{1}H\}$ NMR (75.5 MHz, $CDCl_3$)
21.65
O
P
Bis(4-methylphenyl)phenylphosphine Oxide (**1b**)
f1 (ppm)

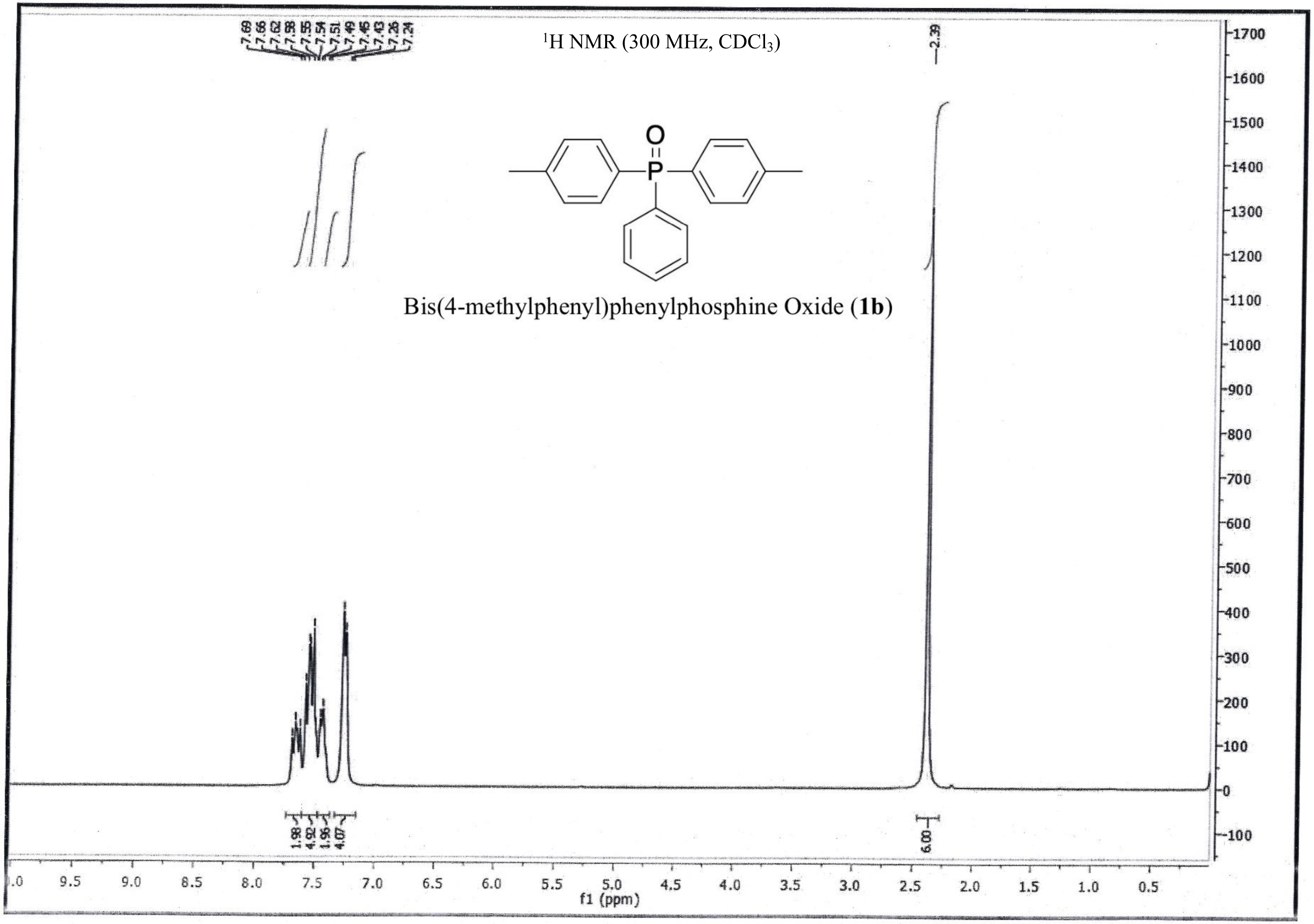

^{1}H NMR (300 MHz, $CDCl_3$)

Bis(4-methylphenyl)phenylphosphine Oxide (**1b**)

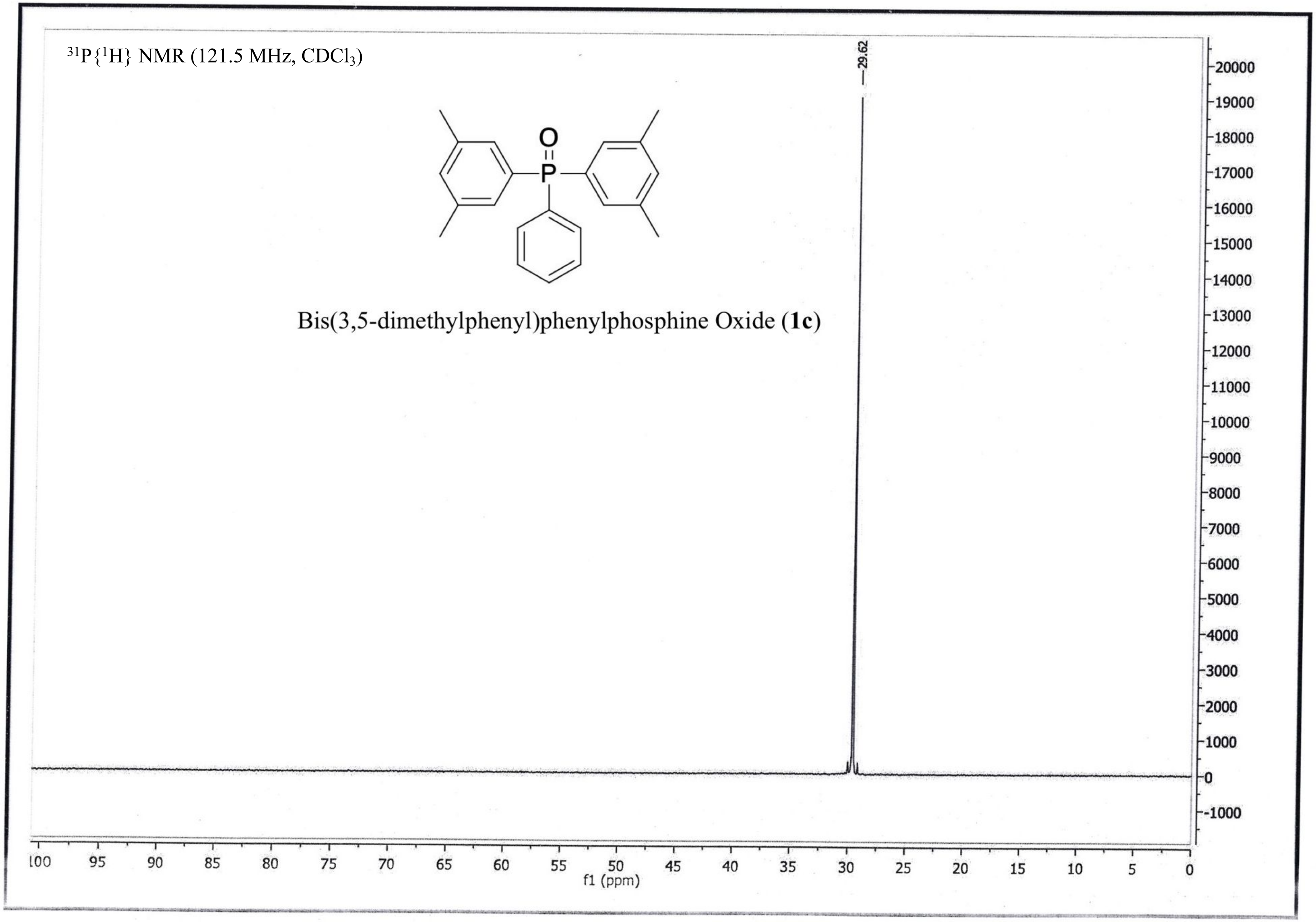

31P{1H} NMR (121.5 MHz, CDCl3)
O
P
Bis(3,5-dimethylphenyl)phenylphosphine Oxide (1c)
29.62
20000
19000
18000
17000
16000
15000
14000
13000
12000
11000
10000
9000
8000
7000
6000
5000
4000
3000
2000
1000
0
-1000
100 95 90 85 80 75 70 65 60 55 50 45 40 35 30 25 20 15 10 5 0
f1 (ppm)

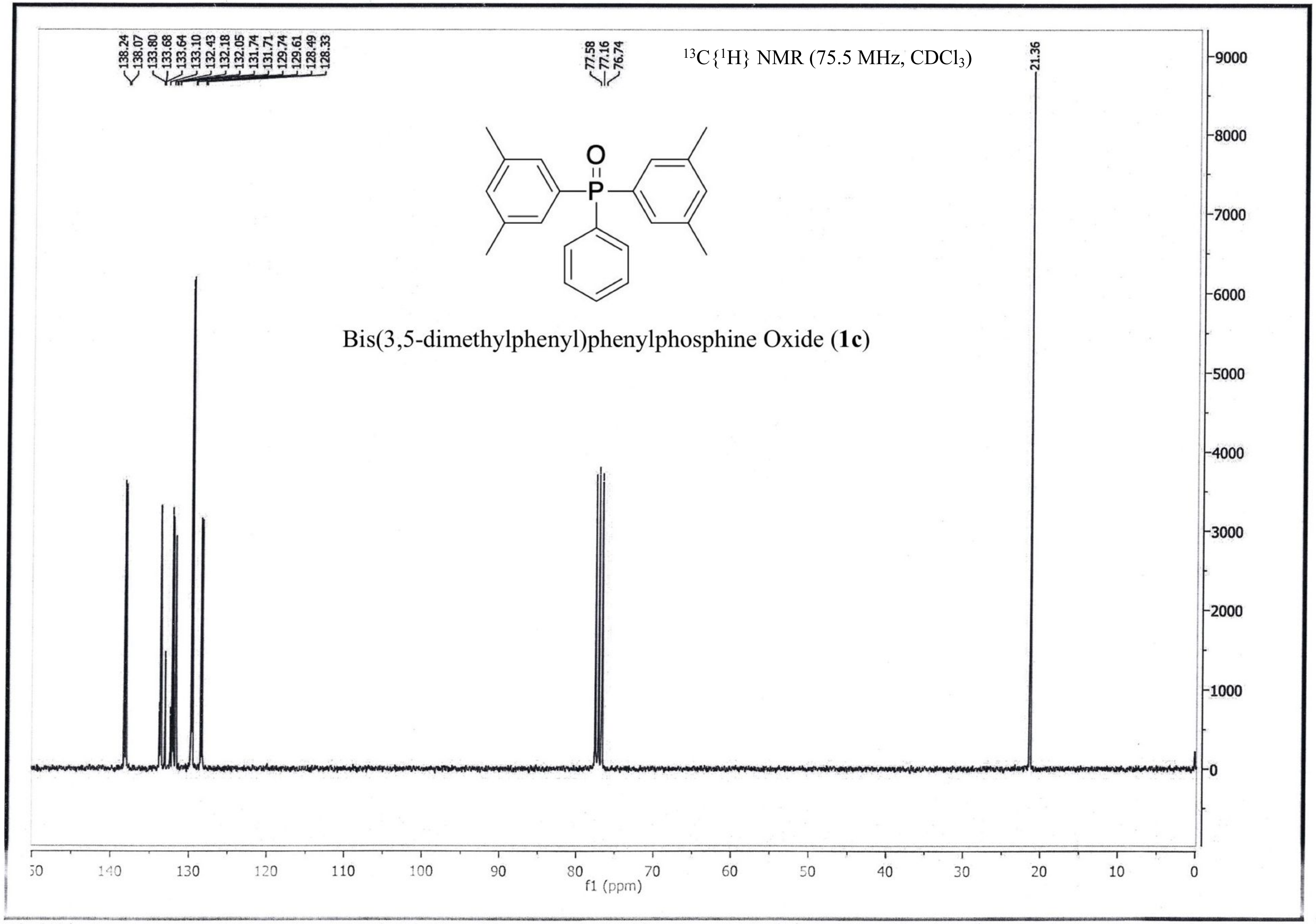
138.24
138.07
133.80
133.68
133.64
133.10
132.43
132.18
132.05
131.74
131.71
129.74
129.61
128.49
128.33
77.58
77.16
76.74
$^{13}C\{^{1}H\}$ NMR (75.5 MHz, $CDCl_3$)
21.36
O
P
Bis(3,5-dimethylphenyl)phenylphosphine Oxide (**1c**)
9000
8000
7000
6000
5000
4000
3000
2000
1000
0
140
130
120
110
100
90
80
70
60
50
40
30
20
10
0
f1 (ppm)

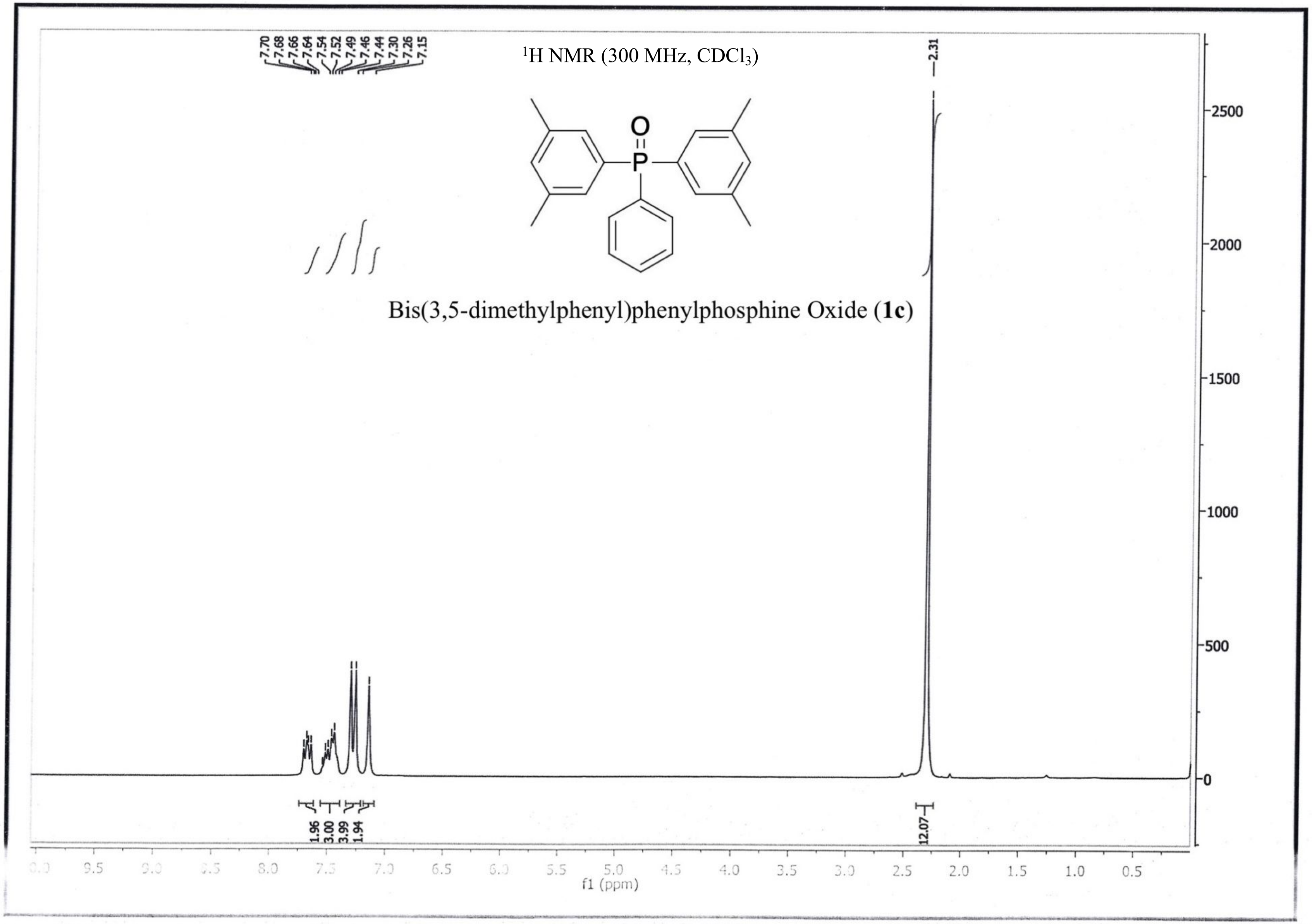
7.70
7.68
7.66
7.64
7.54
7.52
7.49
7.46
7.44
7.30
7.26
7.15
2.31
^{1}H NMR (300 MHz, $CDCl_3$)
Bis(3,5-dimethylphenyl)phenylphosphine Oxide (**1c**)
1.96
3.00
3.99
1.94
12.07
f1 (ppm)

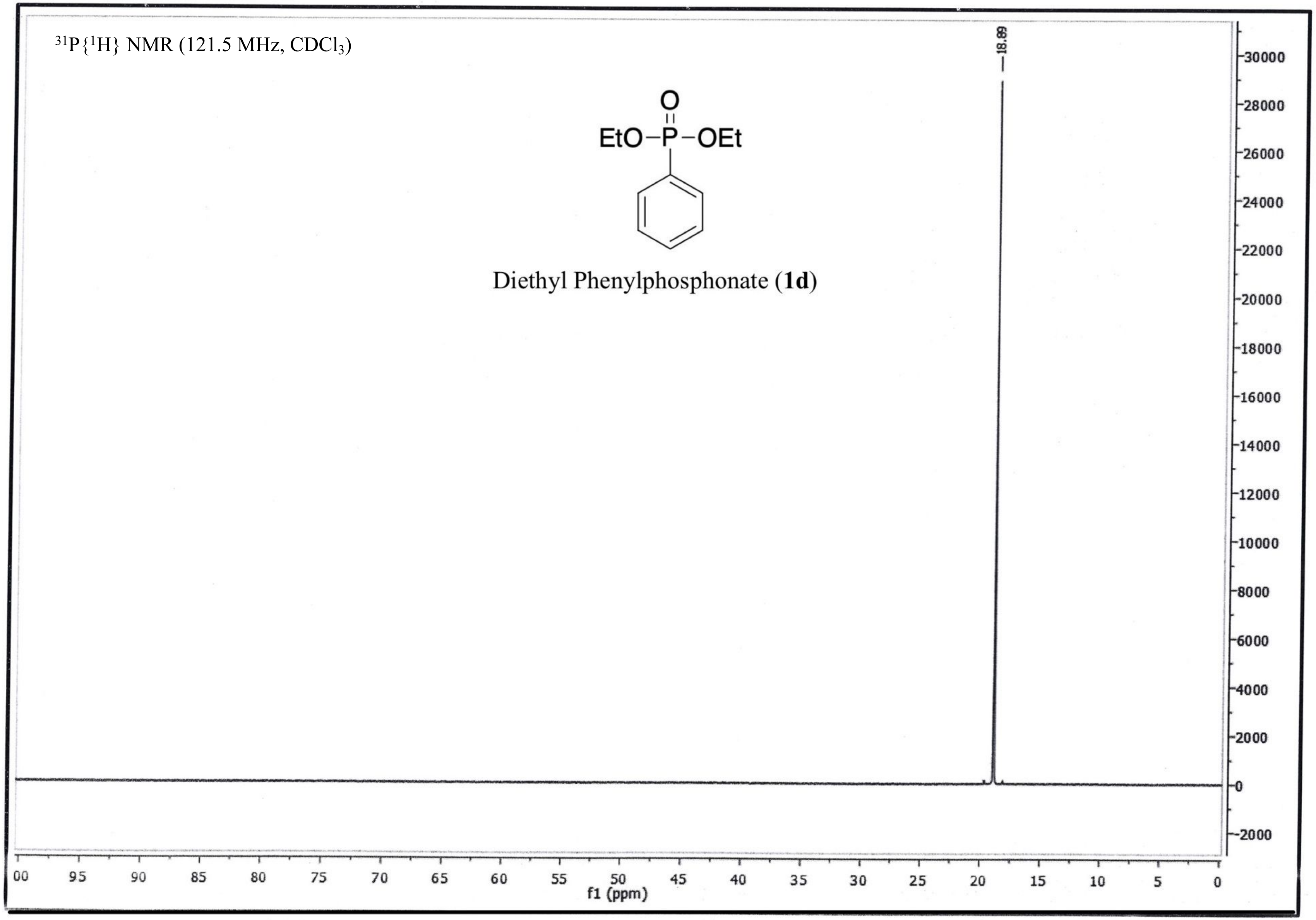
$^{31}P\{^1H\}$ NMR (121.5 MHz, $CDCl_3$)
EtO–P–OEt
O
Diethyl Phenylphosphonate (**1d**)
18.89
f1 (ppm)

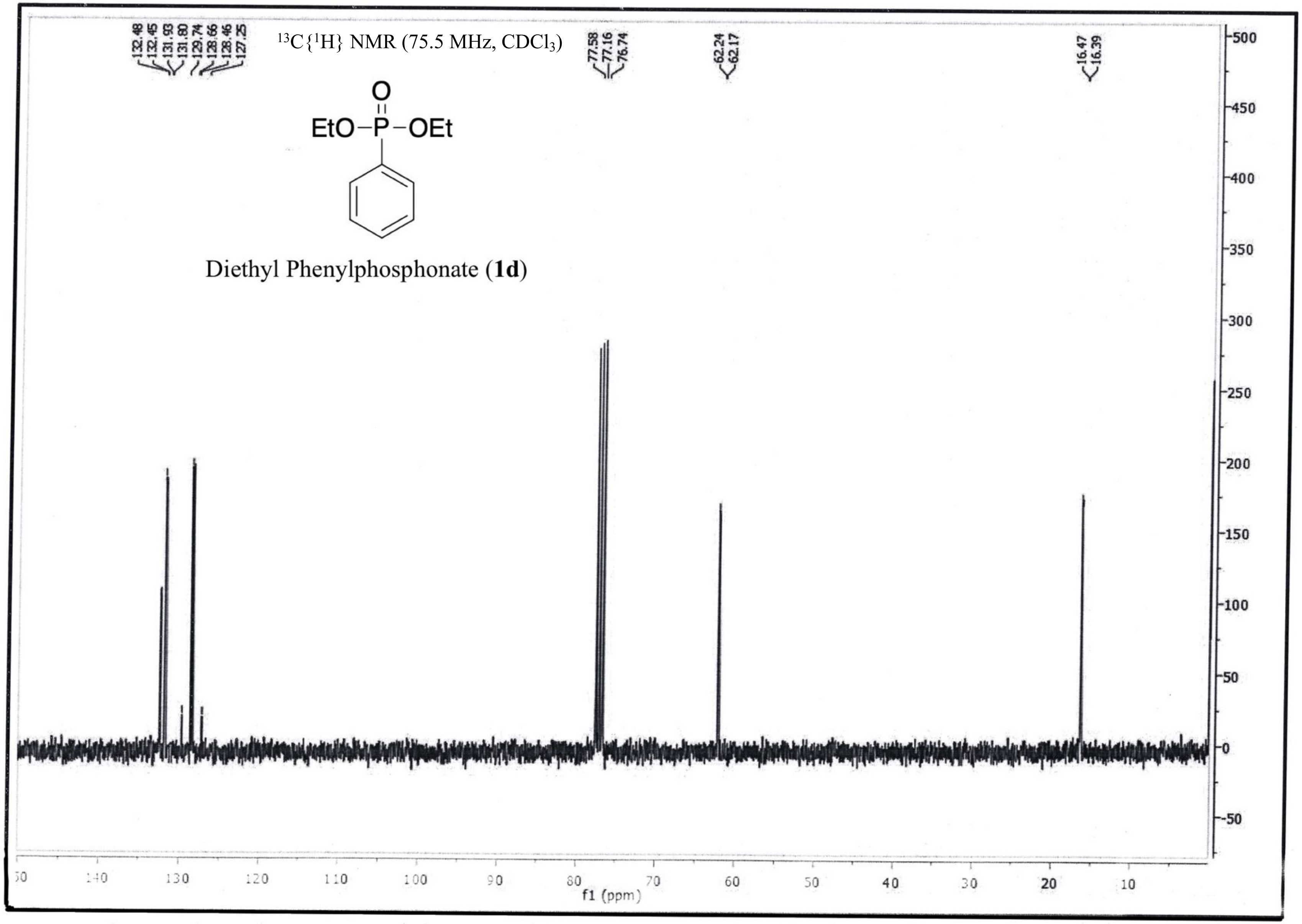
132.48
132.45
131.93
131.80
129.74
128.66
128.46
127.25
$^{13}C\{^1H\}$ NMR (75.5 MHz, $CDCl_3$)
77.58
77.16
76.74
62.24
62.17
16.47
16.39
EtO–P–OEt
O
Diethyl Phenylphosphonate (**1d**)
f1 (ppm)

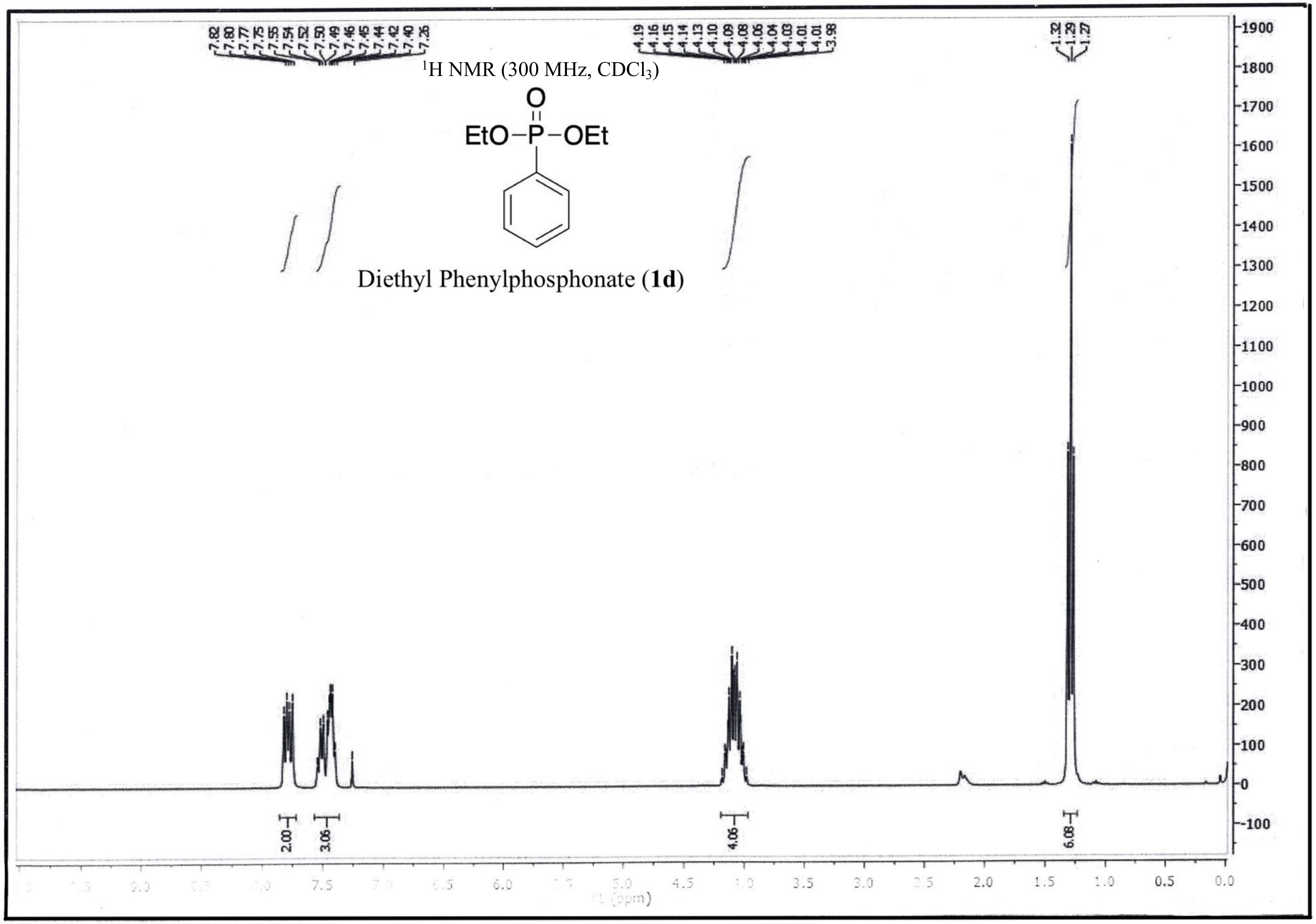

7.82 7.80 7.77 7.75 7.55 7.54 7.52 7.50 7.49 7.46 7.45 7.44 7.42 7.40 7.26
4.19 4.16 4.15 4.14 4.13 4.10 4.09 4.08 4.06 4.04 4.03 4.01 4.01 3.98
1.32 1.29 1.27
¹H NMR (300 MHz, CDCl₃)
EtO–P–OEt
O
Diethyl Phenylphosphonate (1d)
2.00
3.06
4.06
6.08
f1 (ppm)

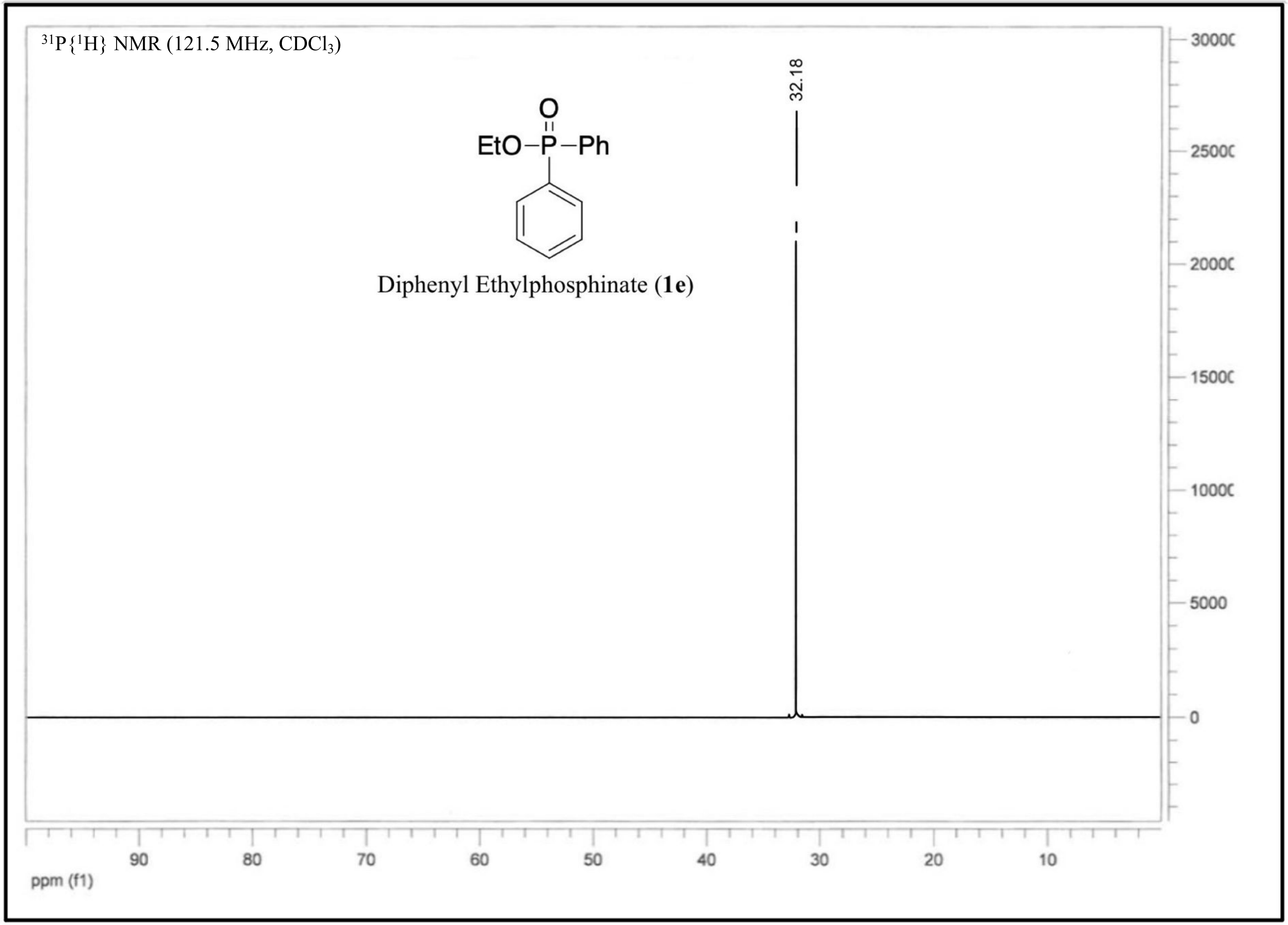

$^{31}P\{^1H\}$ NMR (121.5 MHz, $CDCl_3$)
32.18
EtO
P
O
Ph
Diphenyl Ethylphosphinate (**1e**)
30000
25000
20000
15000
10000
5000
0
90
80
70
60
50
40
30
20
10
ppm (f1)

$^{13}C\{^1H\}$ NMR (75.5 MHz, $CDCl_3$)

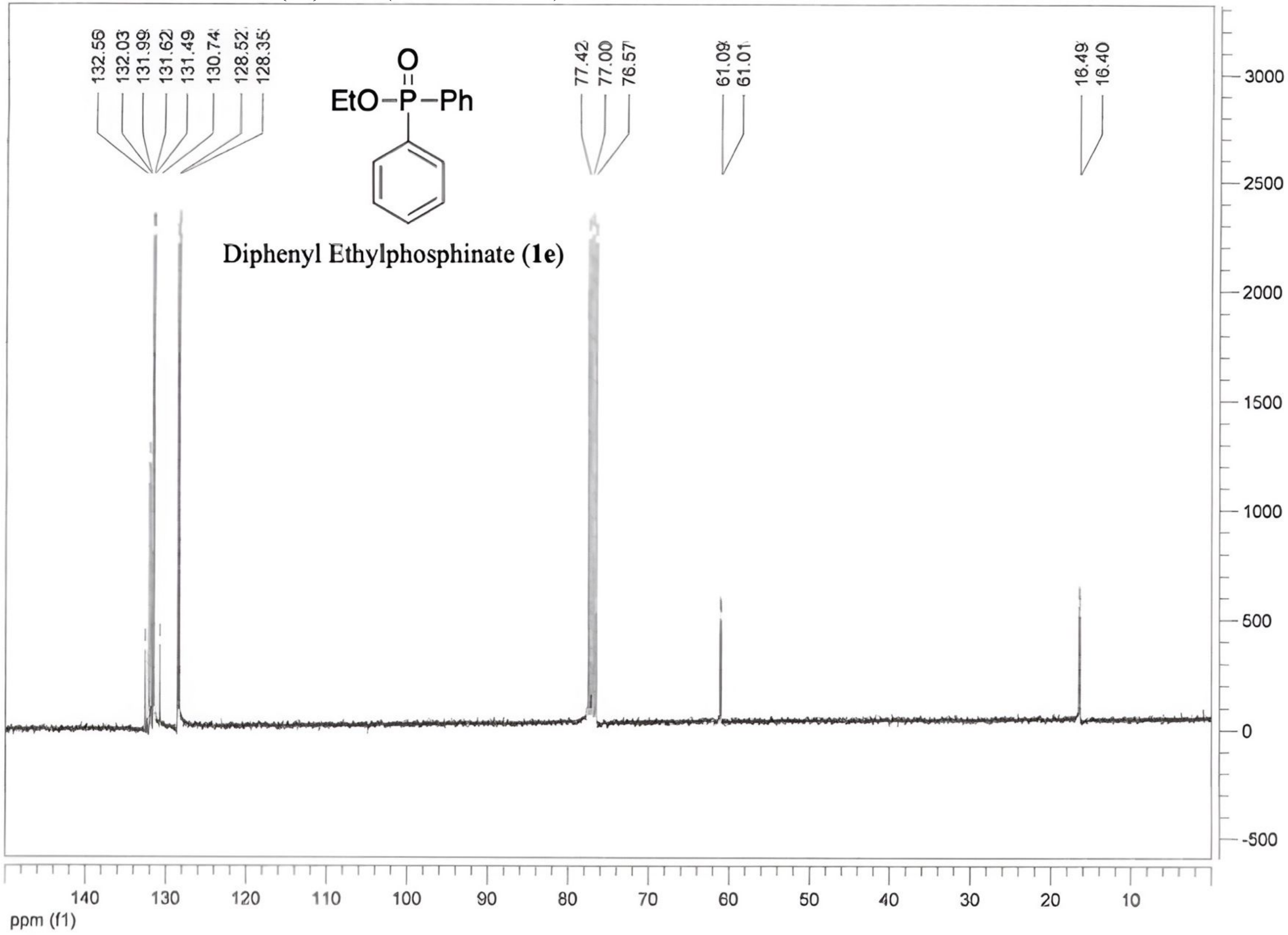

Diphenyl Ethylphosphinate (**1e**)

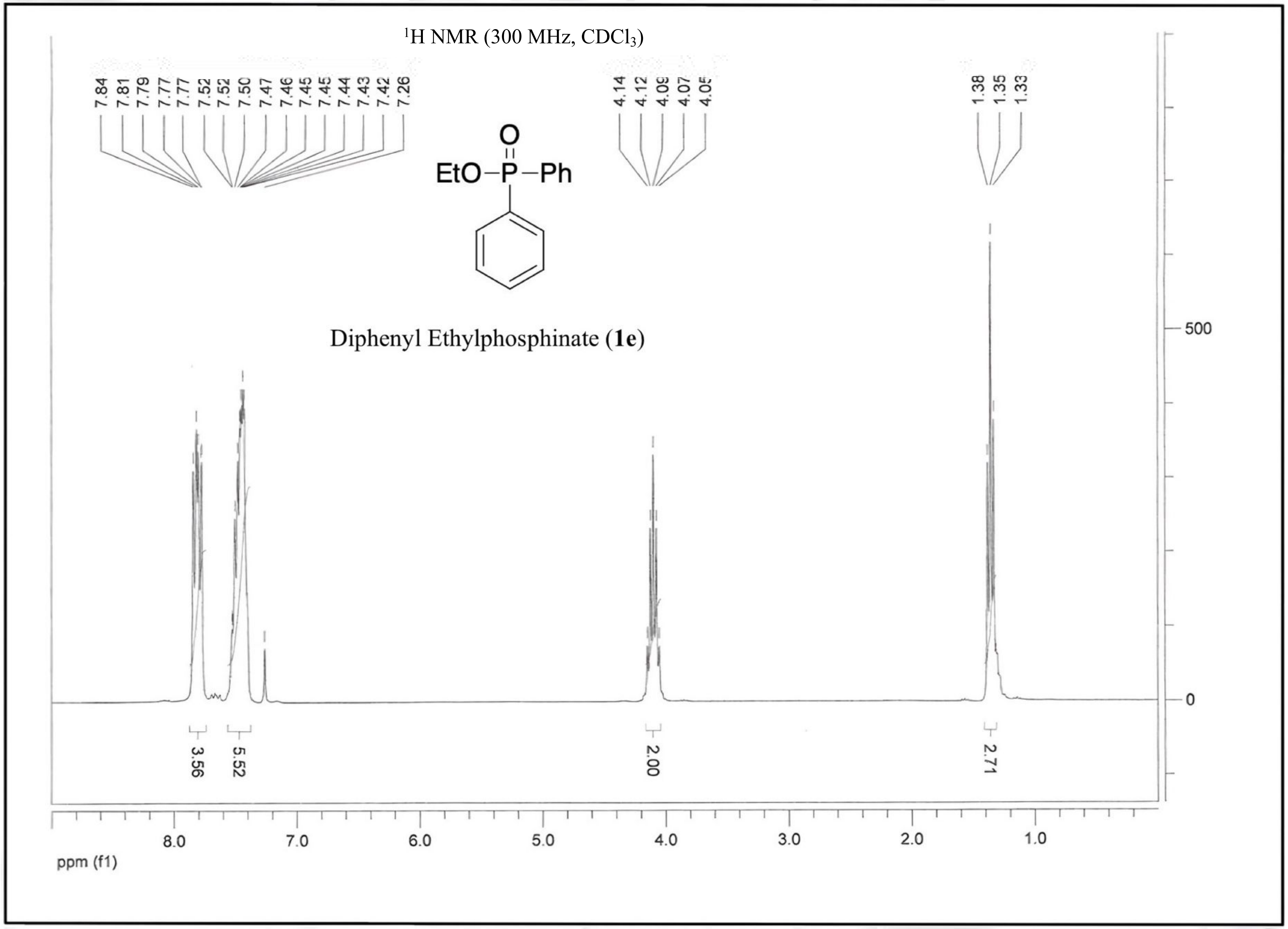
1H NMR (300 MHz, CDCl3)
7.84 7.81 7.79 7.77 7.77 7.52 7.52 7.50 7.47 7.46 7.45 7.45 7.44 7.43 7.42 7.26
4.14 4.12 4.09 4.07 4.05
1.38 1.35 1.33
O
EtO
P
Ph
Diphenyl Ethylphosphinate (1e)
500
0
3.56
5.52
2.00
2.71
8.0
7.0
6.0
5.0
4.0
3.0
2.0
1.0
ppm (f1)

$^{31}P\{^{1}H\}$ NMR (121.5 MHz, $CDCl_3$)

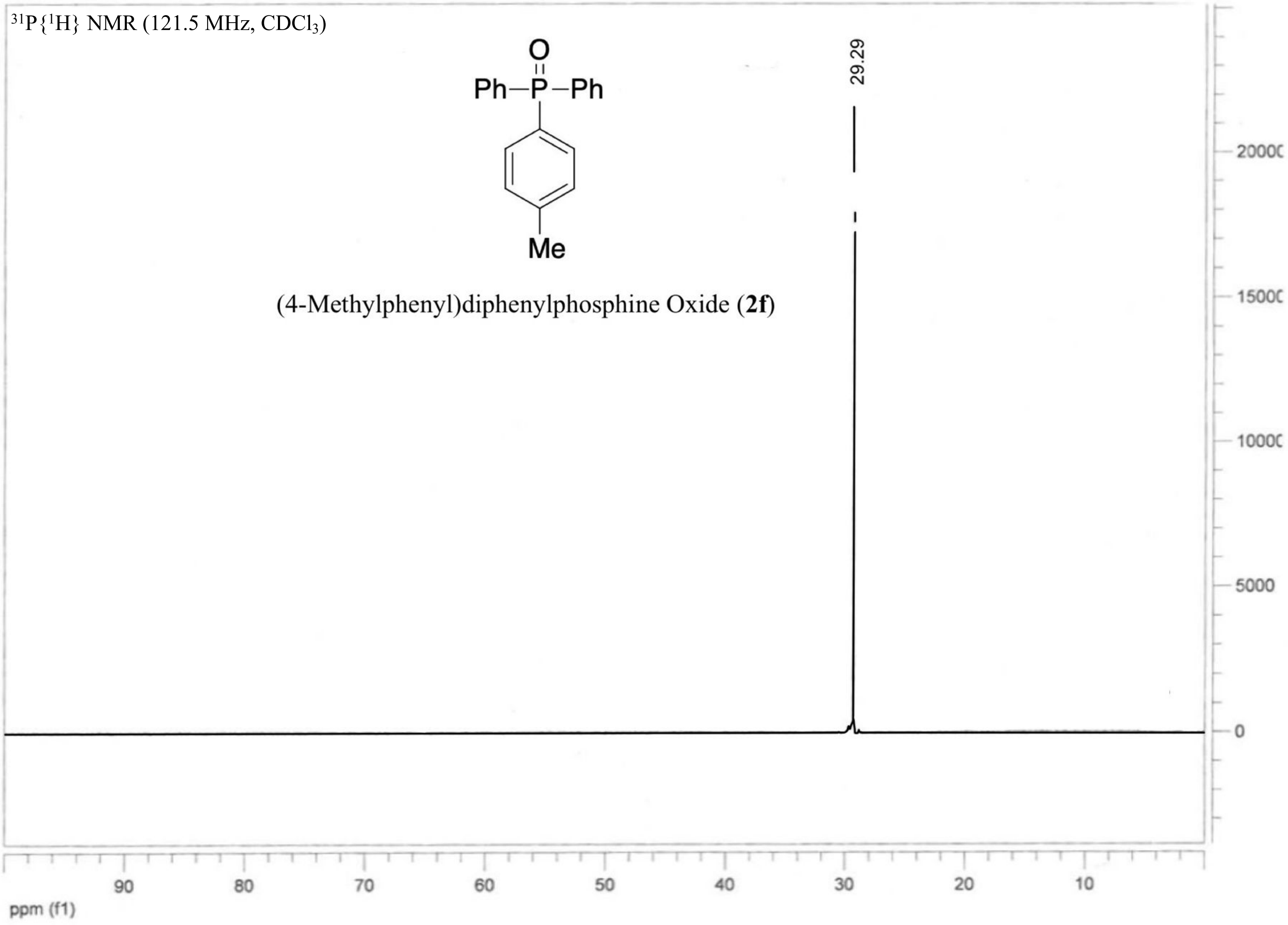

(4-Methylphenyl)diphenylphosphine Oxide (**2f**)

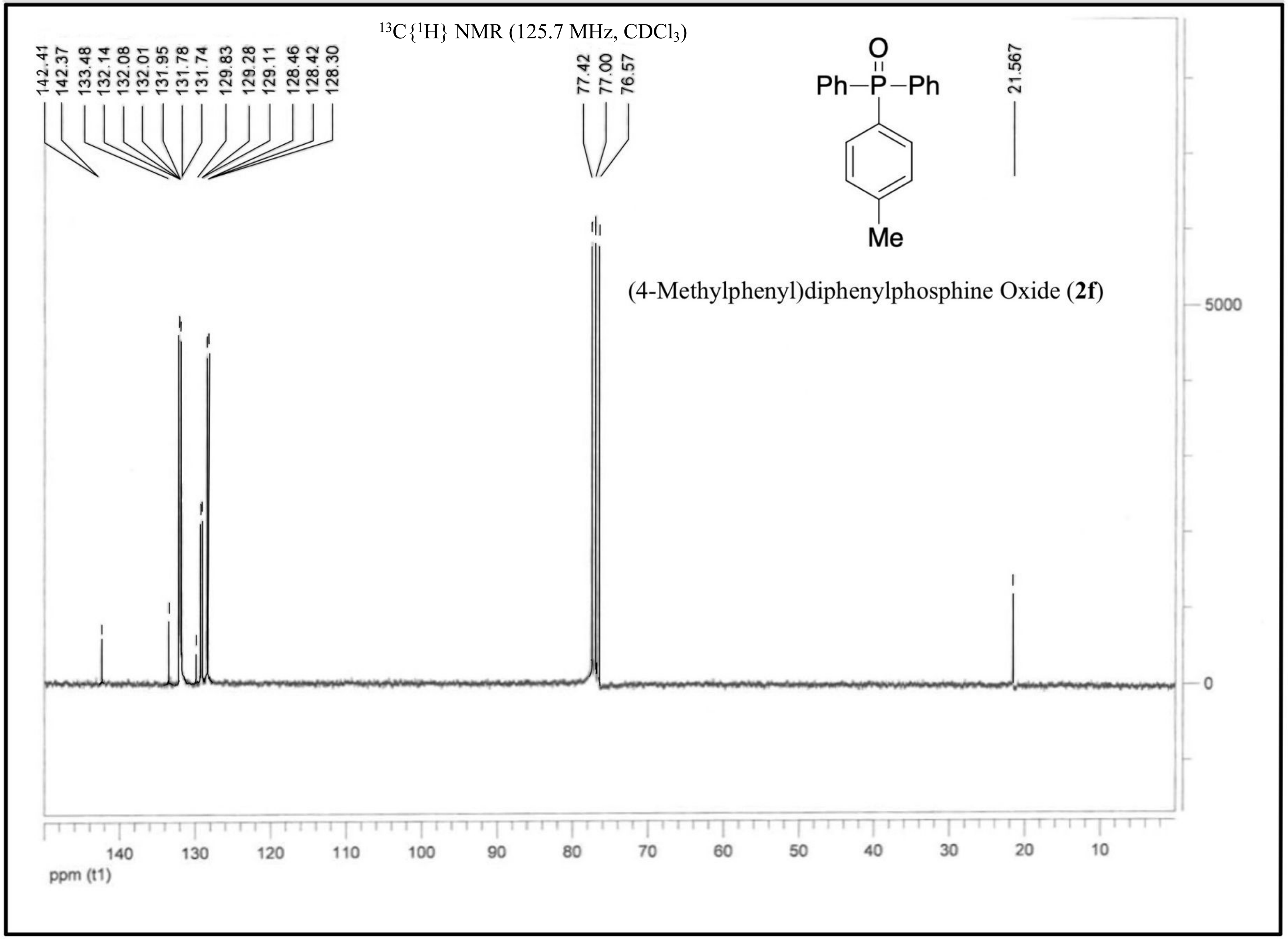
$^{13}C\{^{1}H\}$ NMR (125.7 MHz, $CDCl_3$)
142.41
142.37
133.48
132.14
132.08
132.01
131.95
131.78
131.74
129.83
129.28
129.11
128.46
128.42
128.30
77.42
77.00
76.57
21.567
O
Ph–P–Ph
Me
(4-Methylphenyl)diphenylphosphine Oxide (**2f**)
5000
0
140
130
120
110
100
90
80
70
60
50
40
30
20
10
ppm (t1)

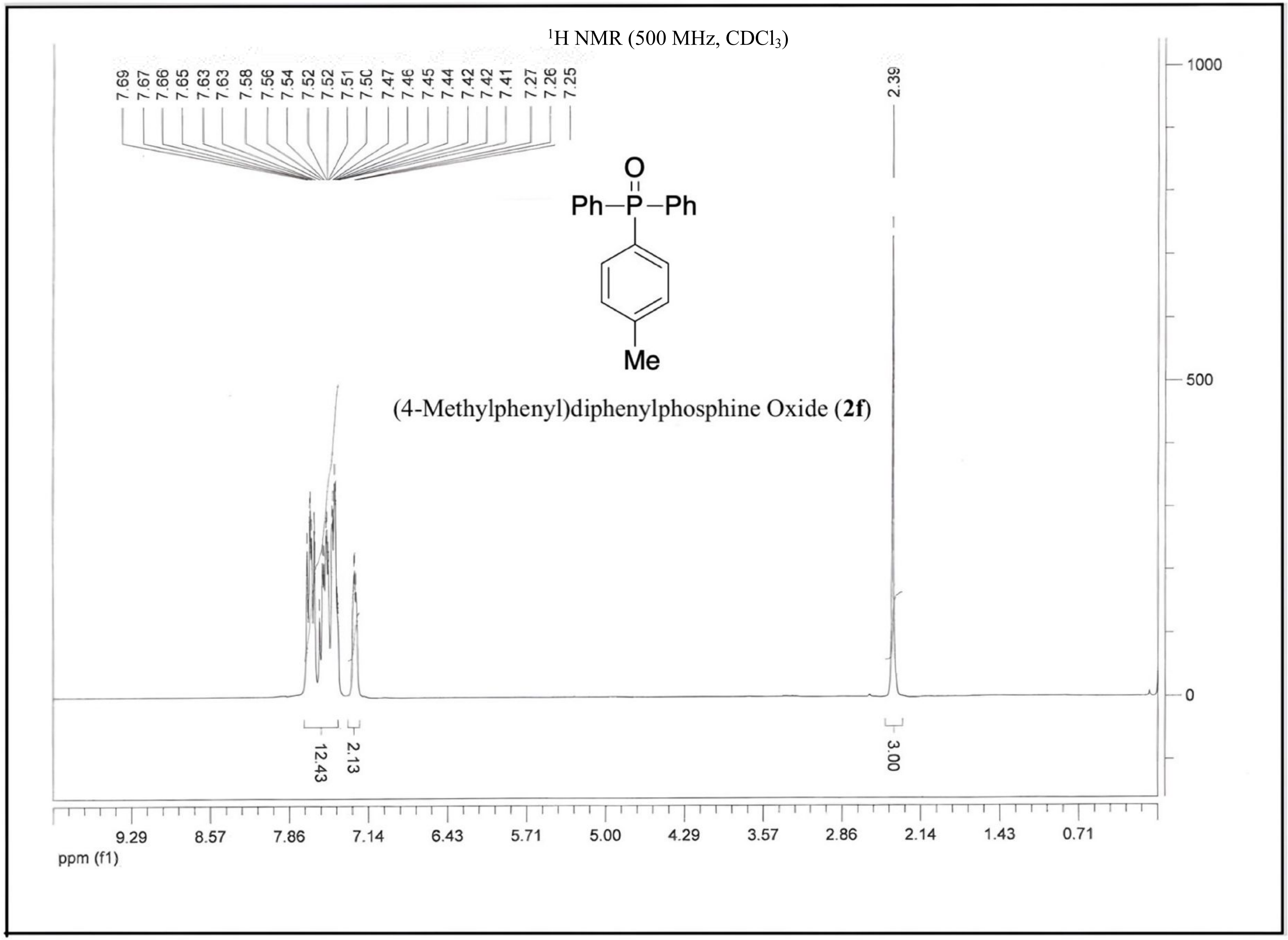

1H NMR (500 MHz, CDCl3)
7.69 7.67 7.66 7.65 7.63 7.63 7.58 7.56 7.54 7.52 7.52 7.51 7.50 7.47 7.46 7.45 7.44 7.42 7.42 7.41 7.27 7.26 7.25
2.39
Ph
P
O
Ph
Me
(4-Methylphenyl)diphenylphosphine Oxide (2f)
1000
500
0
12.43
2.13
3.00
9.29 8.57 7.86 7.14 6.43 5.71 5.00 4.29 3.57 2.86 2.14 1.43 0.71
ppm (f1)

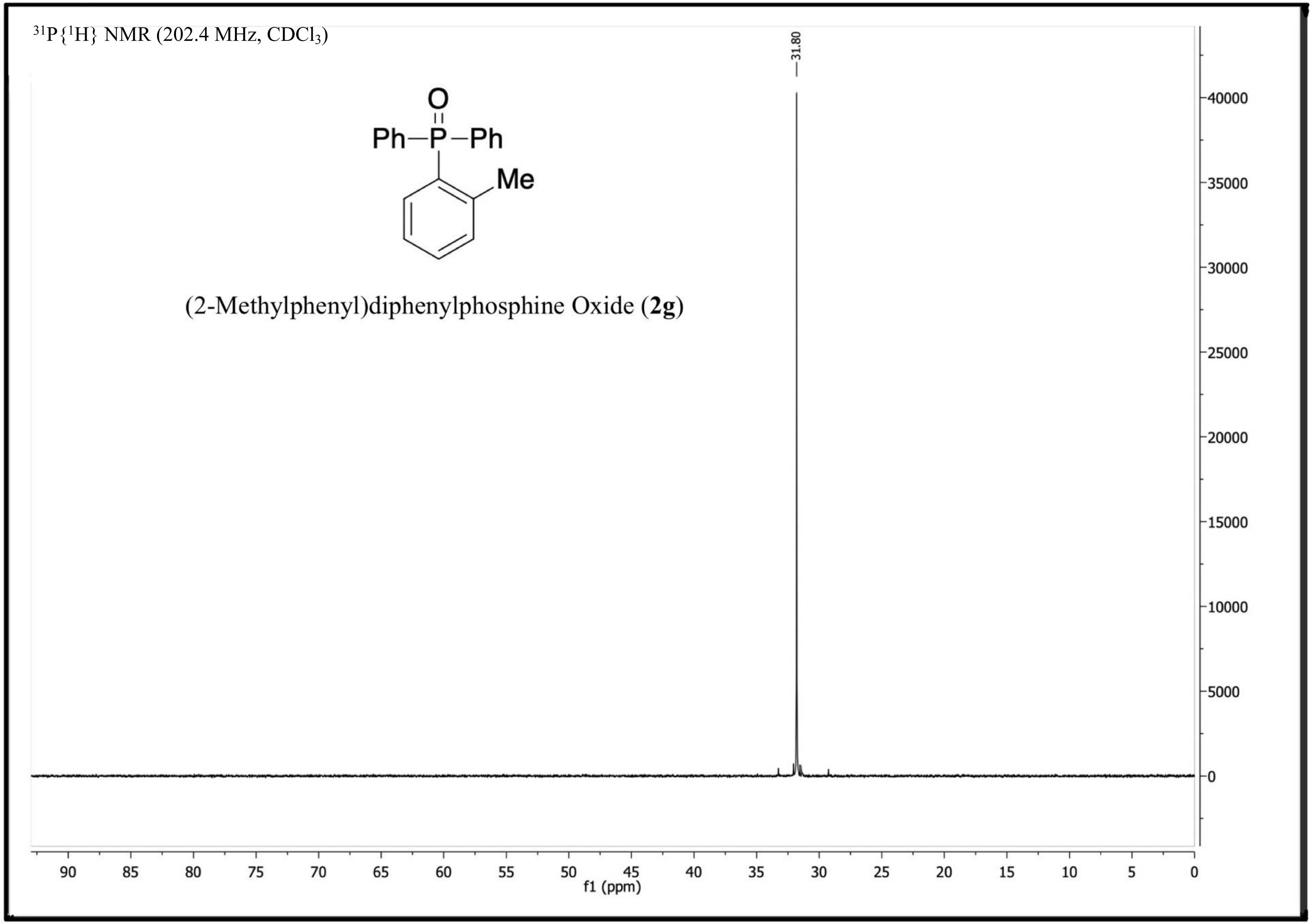

$^{31}P\{^1H\}$ NMR (202.4 MHz, $CDCl_3$)
31.80
O
Ph
P
Ph
Me
(2-Methylphenyl)diphenylphosphine Oxide (**2g**)
40000
35000
30000
25000
20000
15000
10000
5000
0
90 85 80 75 70 65 60 55 50 45 40 35 30 25 20 15 10 5 0
f1 (ppm)

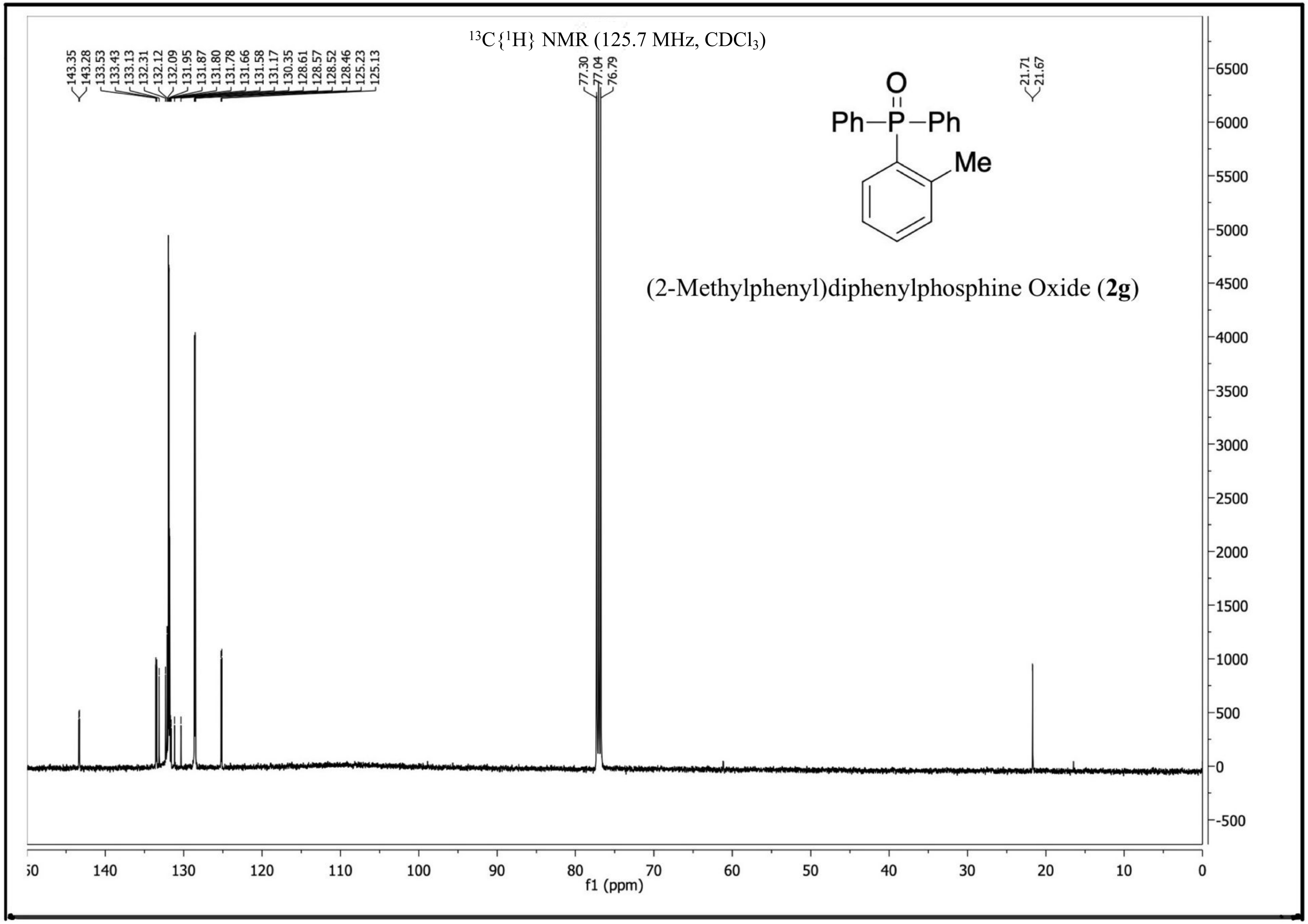

13C{1H} NMR (125.7 MHz, CDCl3)
143.35 143.28 133.53 133.43 133.13 132.31 132.12 132.09 131.95 131.87 131.80 131.78 131.66 131.58 131.17 130.35 128.61 128.57 128.52 128.46 125.23 125.13
77.30 77.04 76.79
21.71 21.67
Ph–P(=O)–Ph
Me
(2-Methylphenyl)diphenylphosphine Oxide (2g)
f1 (ppm)

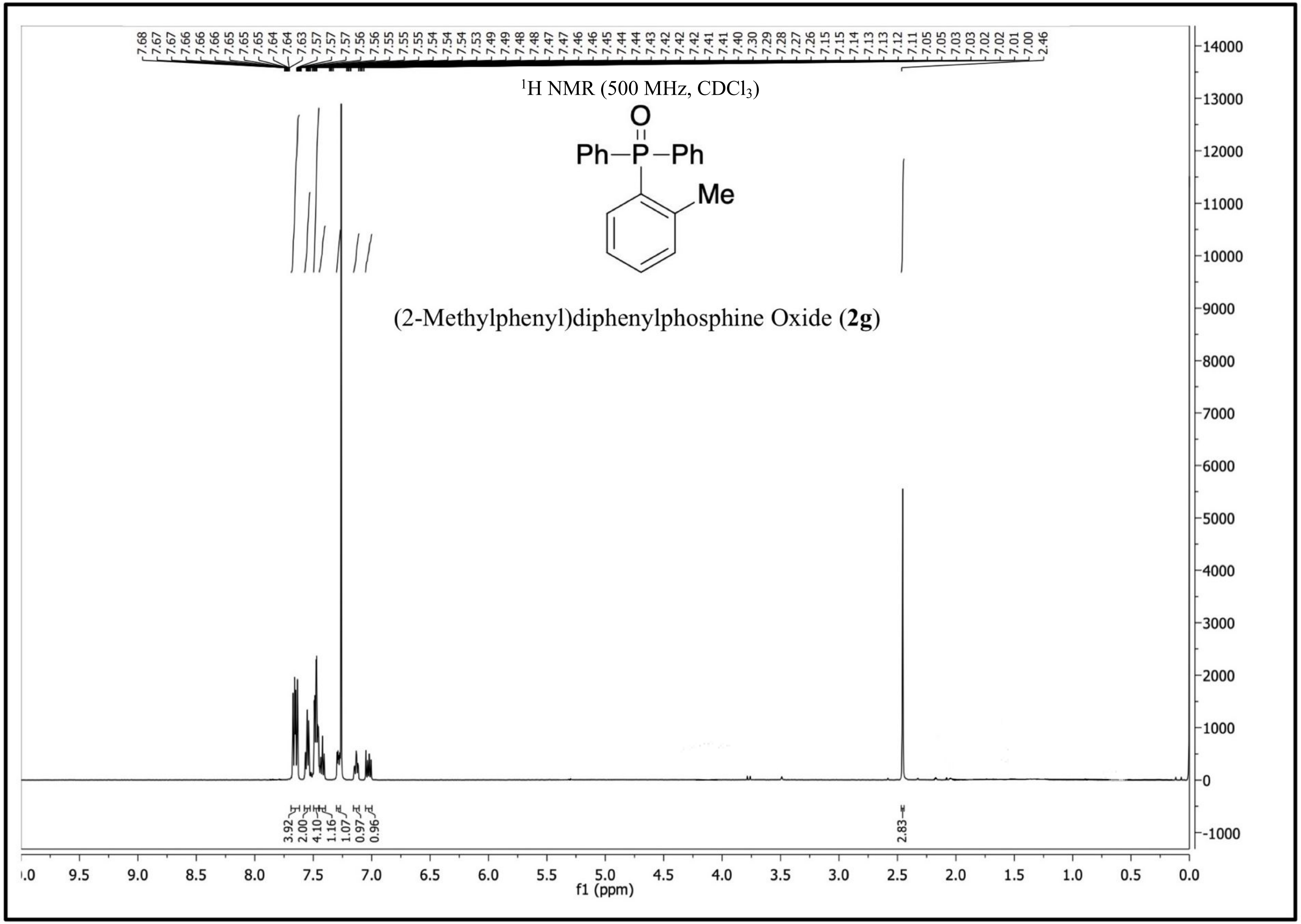

¹H NMR (500 MHz, CDCl₃)
Ph–P–Ph
O
Me
(2-Methylphenyl)diphenylphosphine Oxide (2g)
f1 (ppm)

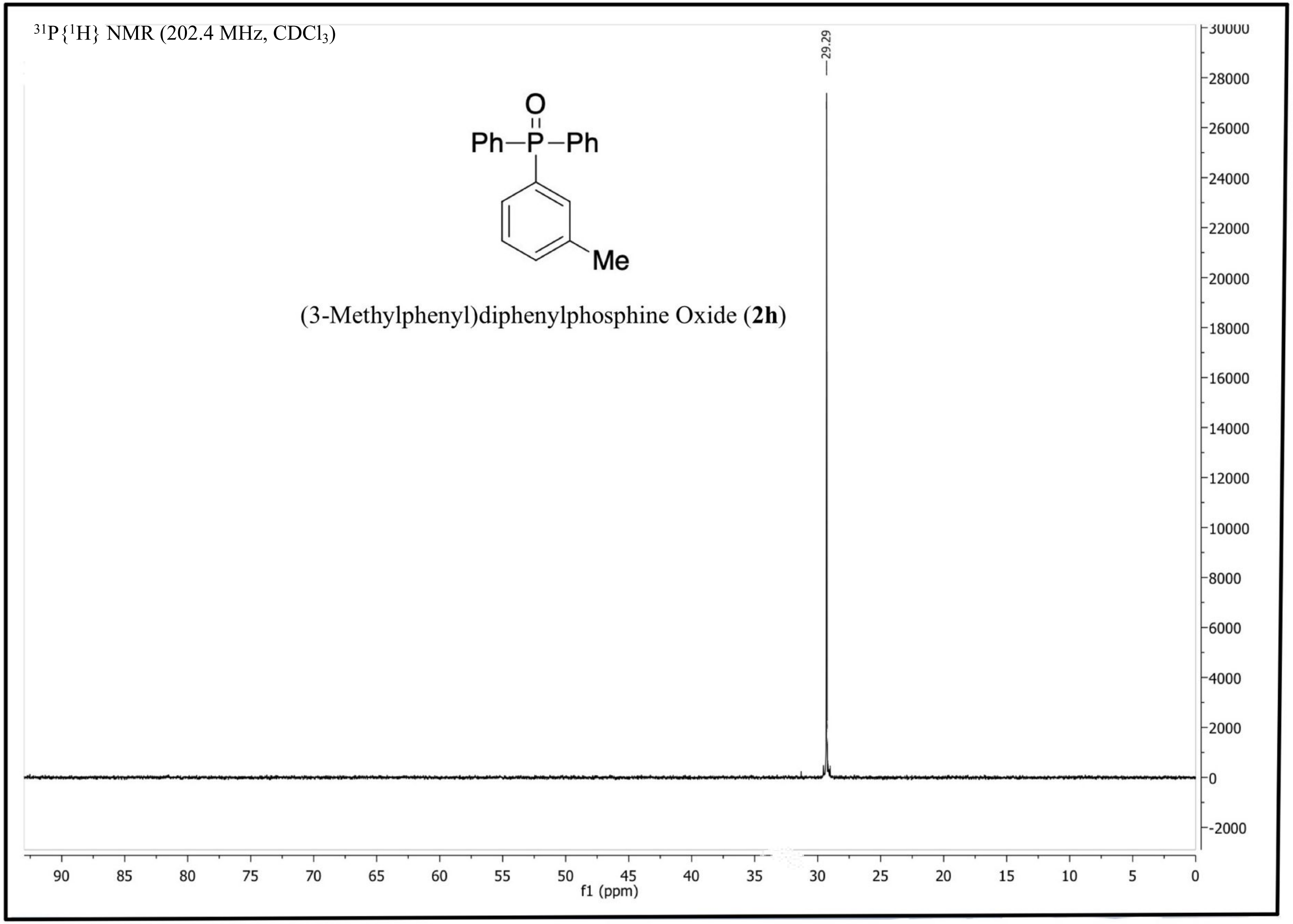

$^{31}P\{^1H\}$ NMR (202.4 MHz, $CDCl_3$)
29.29
O
Ph
P
Ph
Me
(3-Methylphenyl)diphenylphosphine Oxide (**2h**)
30000
28000
26000
24000
22000
20000
18000
16000
14000
12000
10000
8000
6000
4000
2000
0
-2000
90
85
80
75
70
65
60
55
50
45
40
35
30
25
20
15
10
5
0
f1 (ppm)

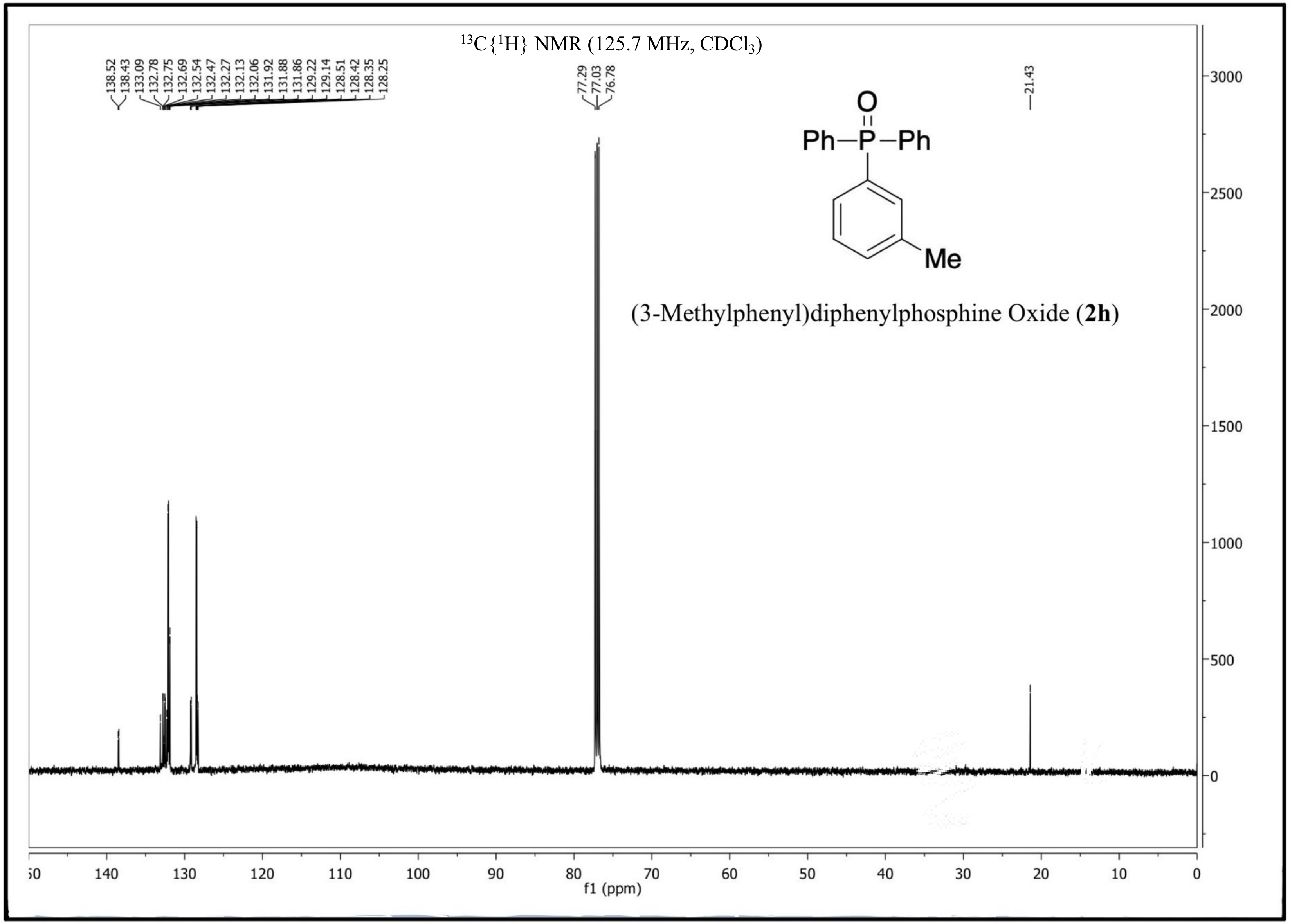
$^{13}C\{^1H\}$ NMR (125.7 MHz, $CDCl_3$)
138.52 138.43 133.09 132.78 132.75 132.69 132.54 132.47 132.27 132.13 132.06 131.92 131.88 131.86 129.22 129.14 128.51 128.42 128.35 128.25
77.29 77.03 76.78
21.43
Ph–P–Ph
O
Me
(3-Methylphenyl)diphenylphosphine Oxide (**2h**)
f1 (ppm)
3000
2500
2000
1500
1000
500
0

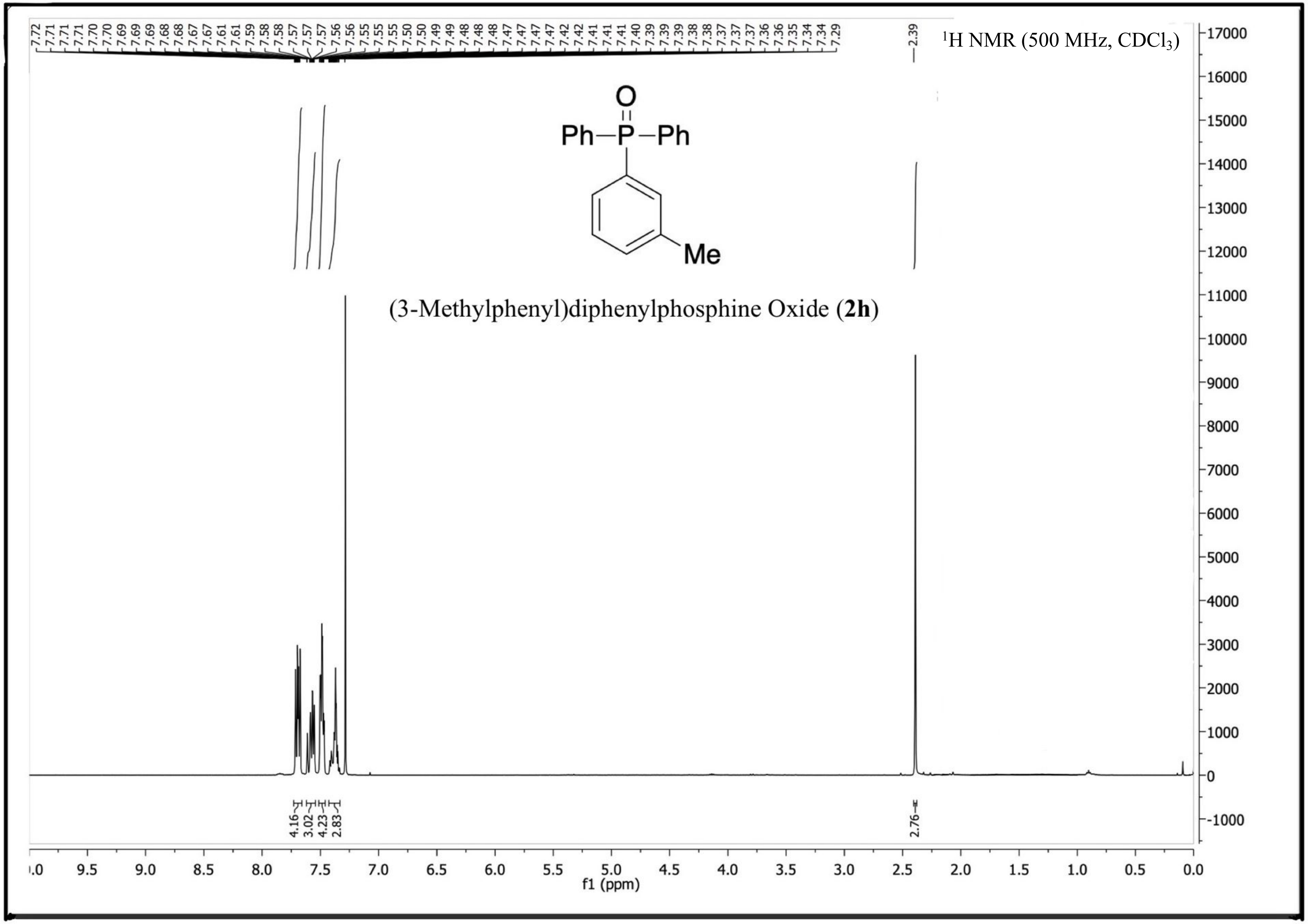

7.72 7.71 7.71 7.71 7.70 7.70 7.69 7.69 7.69 7.68 7.67 7.67 7.67 7.61 7.59 7.58 7.58 7.57 7.57 7.57 7.57 7.56 7.56 7.55 7.55 7.55 7.50 7.50 7.49 7.49 7.48 7.48 7.48 7.47 7.47 7.47 7.47 7.42 7.42 7.41 7.41 7.41 7.40 7.39 7.39 7.39 7.38 7.38 7.37 7.37 7.37 7.36 7.36 7.35 7.34 7.34 7.29
2.39
1H NMR (500 MHz, CDCl3)
O
Ph
P
Ph
Me
(3-Methylphenyl)diphenylphosphine Oxide (2h)
4.16
3.02
4.23
2.83
2.76
f1 (ppm)

$^{31}P\{^{1}H\}$ NMR (121.5 MHz, $CDCl_3$)

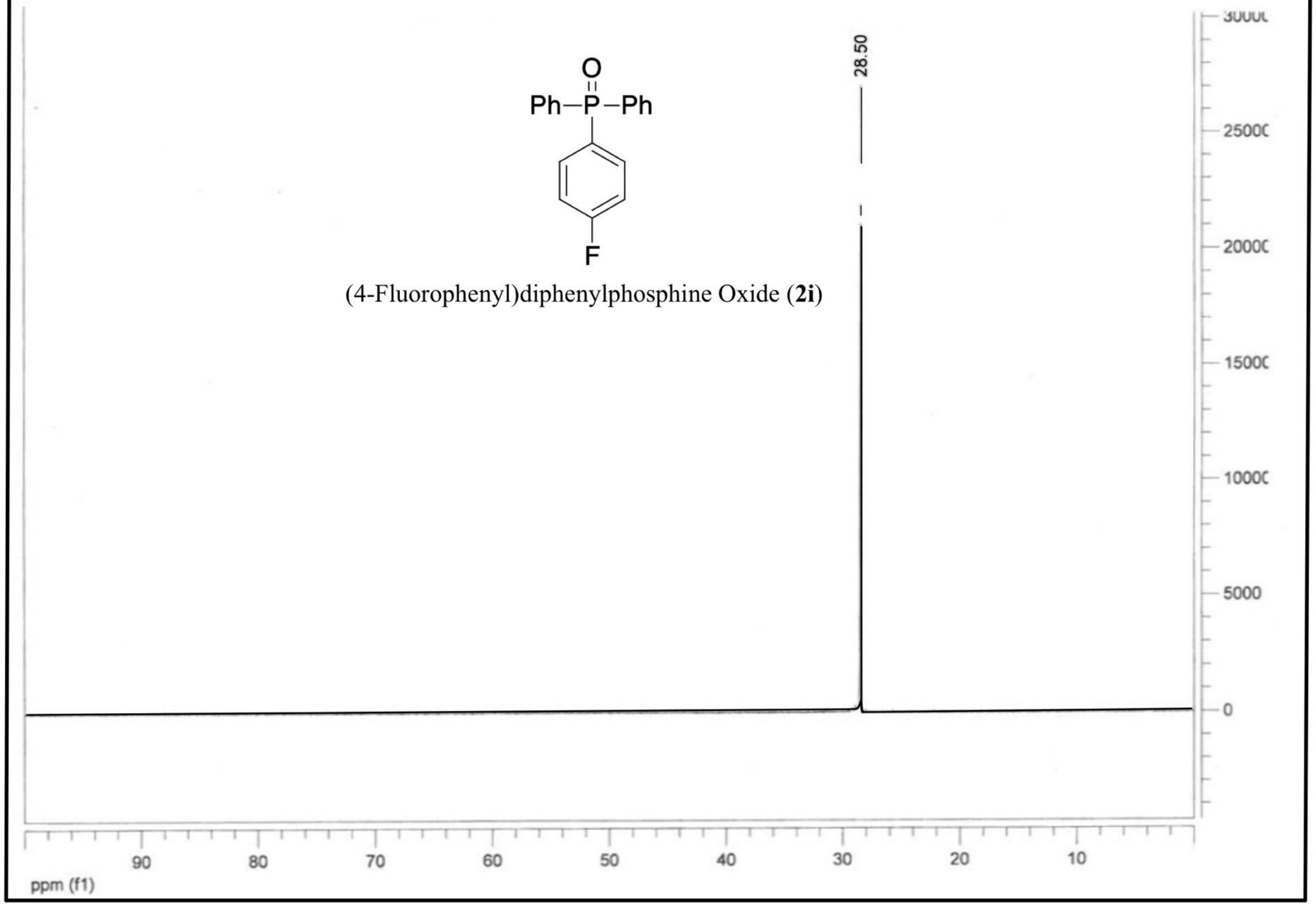

(4-Fluorophenyl)diphenylphosphine Oxide (**2i**)

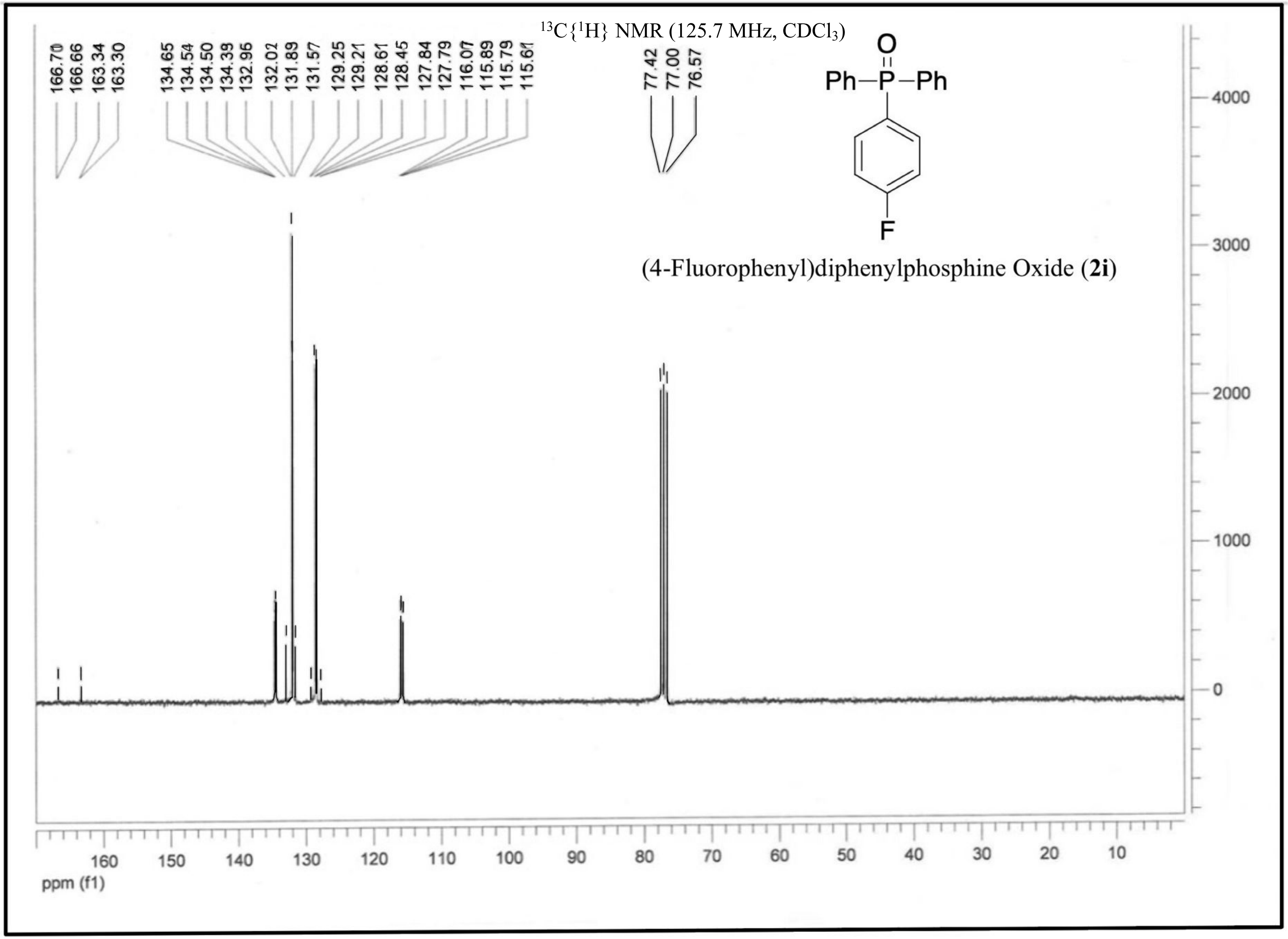

(4-Fluorophenyl)diphenylphosphine Oxide (**2i**)

^{1}H NMR (500 MHz, $CDCl_3$)

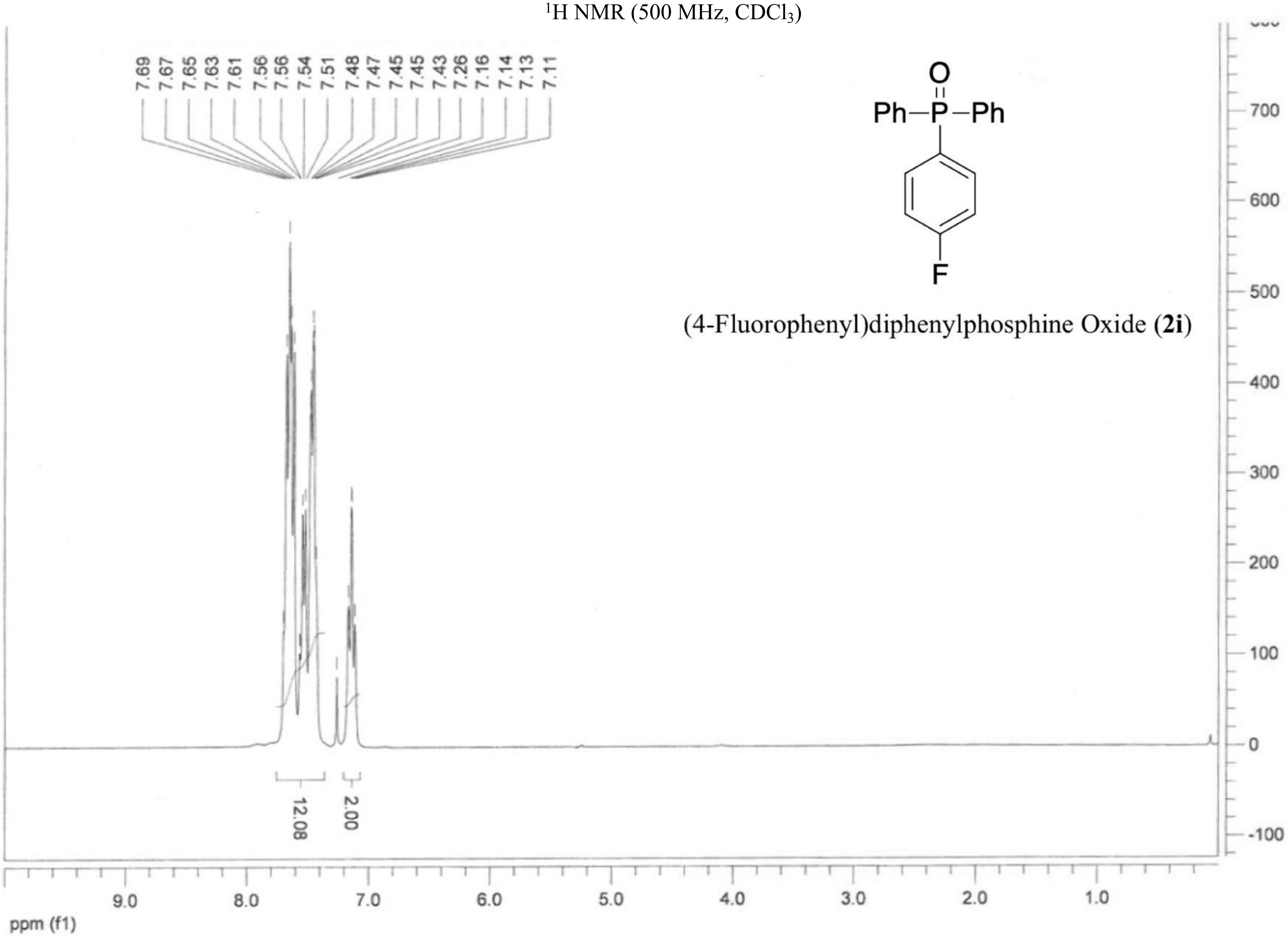

(4-Fluorophenyl)diphenylphosphine Oxide (**2i**)

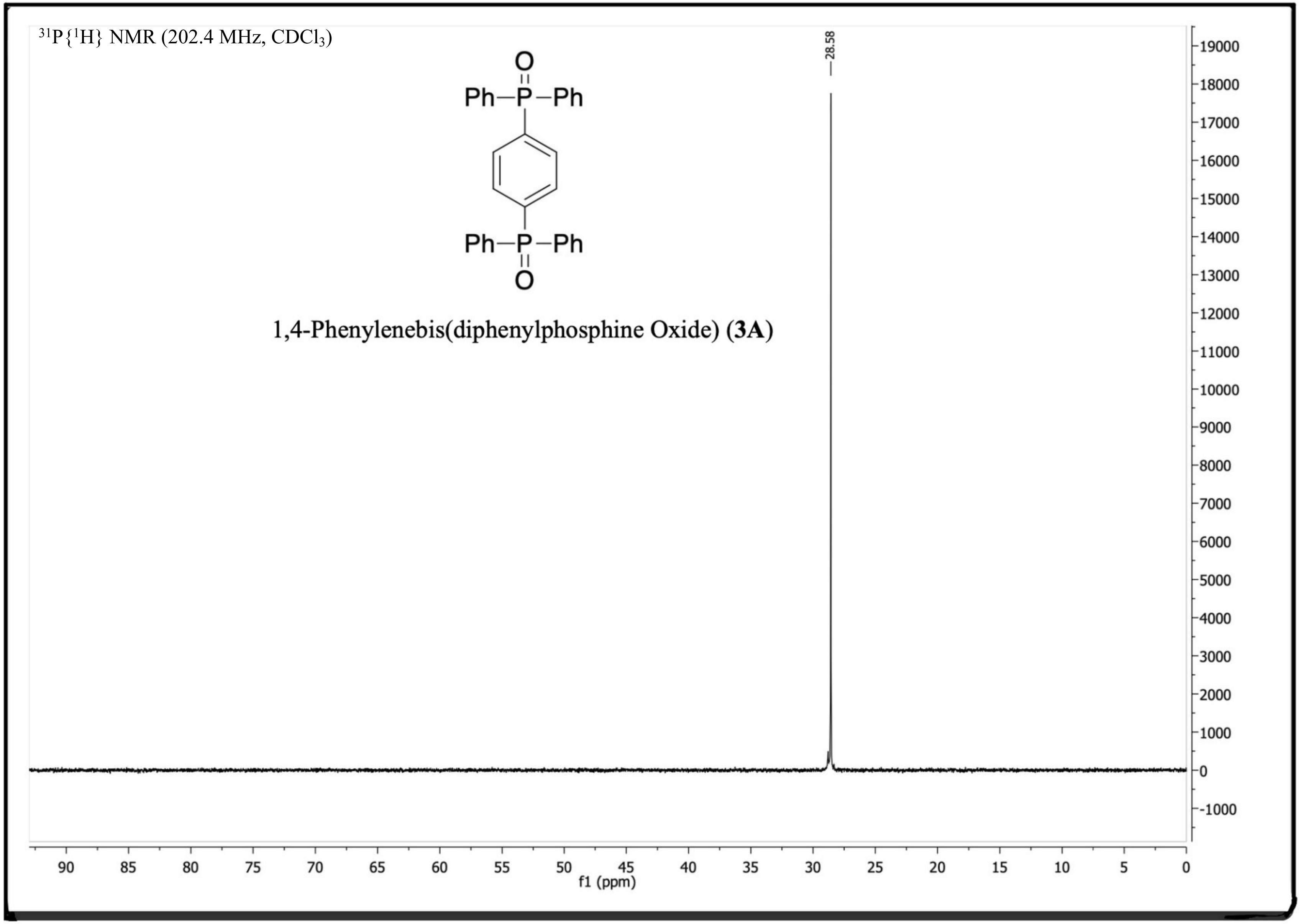

$^{31}P\{^{1}H\}$ NMR (202.4 MHz, $CDCl_3$)
28.58
Ph
P
O
1,4-Phenylenebis(diphenylphosphine Oxide) (**3A**)
f1 (ppm)

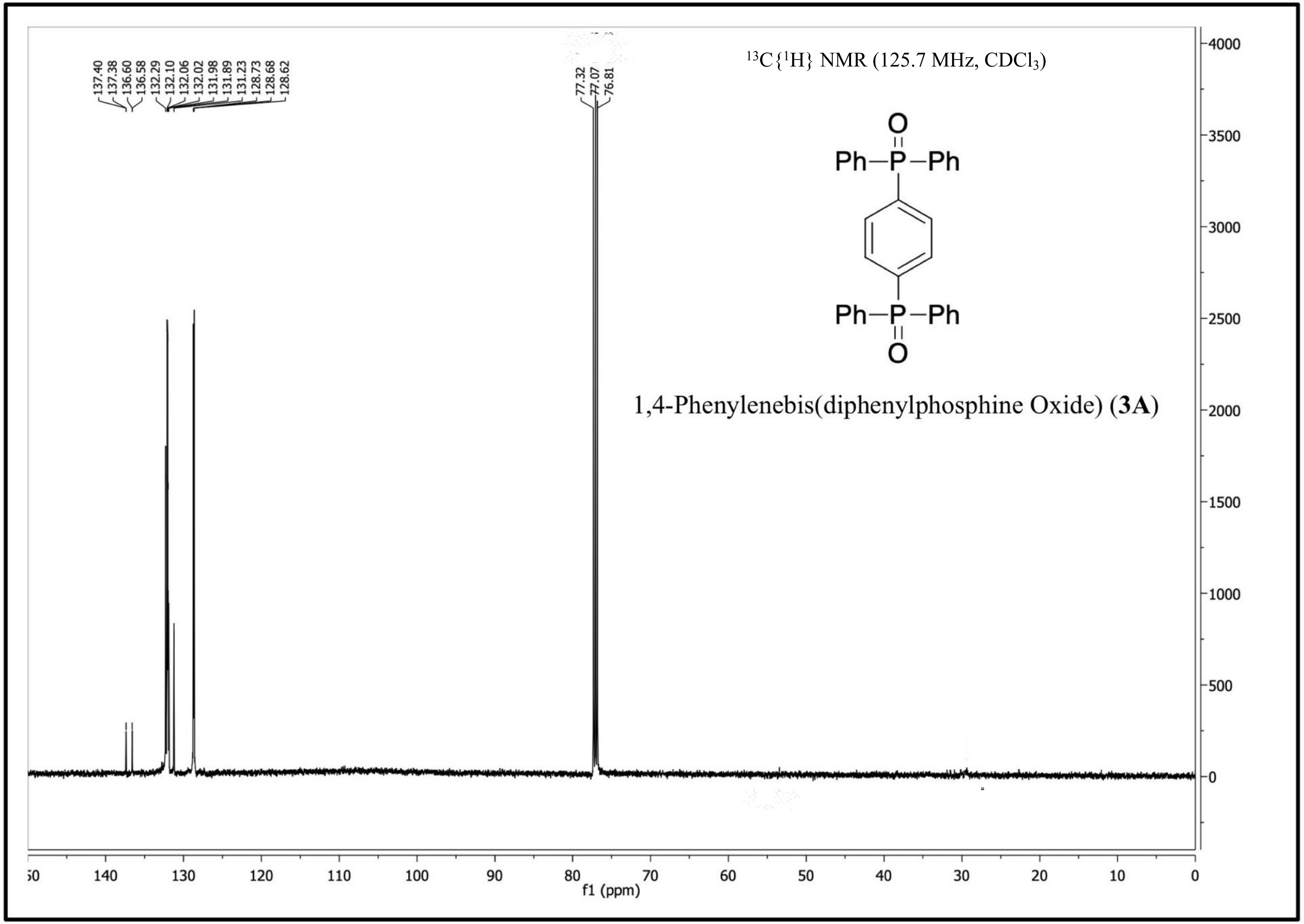

137.40
137.38
136.60
136.58
132.29
132.10
132.06
132.02
131.98
131.89
131.23
128.73
128.68
128.62
77.32
77.07
76.81
$^{13}C\{^1H\}$ NMR (125.7 MHz, CDCl$_3$)
Ph
P
O
1,4-Phenylenebis(diphenylphosphine Oxide) (**3A**)
f1 (ppm)

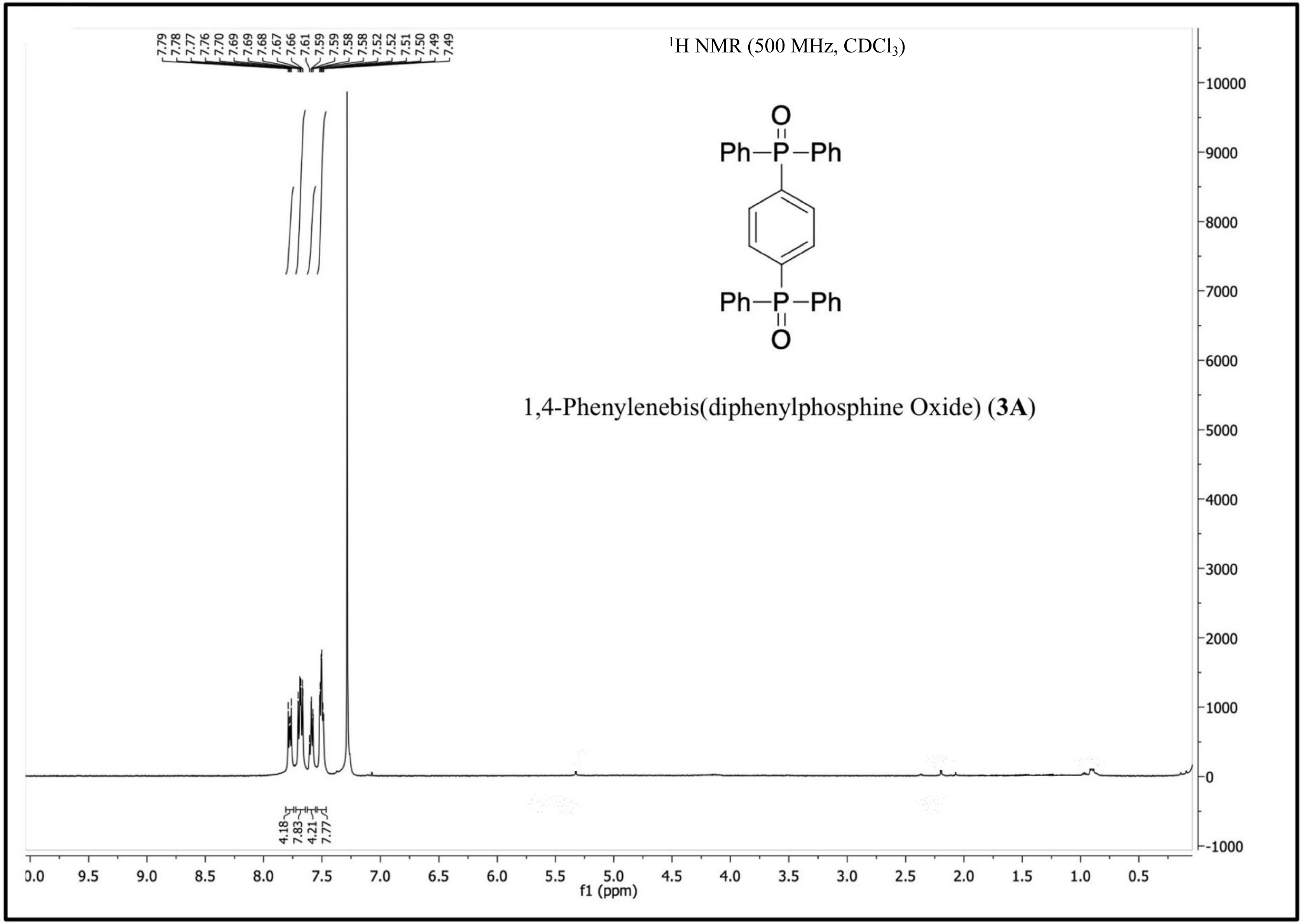

^{1}H NMR (500 MHz, CDCl$_3$)
1,4-Phenylenebis(diphenylphosphine Oxide) (**3A**)
Ph–P–Ph
O
7.79 7.78 7.77 7.76 7.70 7.69 7.69 7.68 7.67 7.66 7.61 7.59 7.59 7.58 7.58 7.52 7.52 7.51 7.50 7.49 7.49
4.18 7.83 4.21 7.77
f1 (ppm)

$^{31}P\{^{1}H\}$ NMR (202.4 MHz, $CDCl_3$)

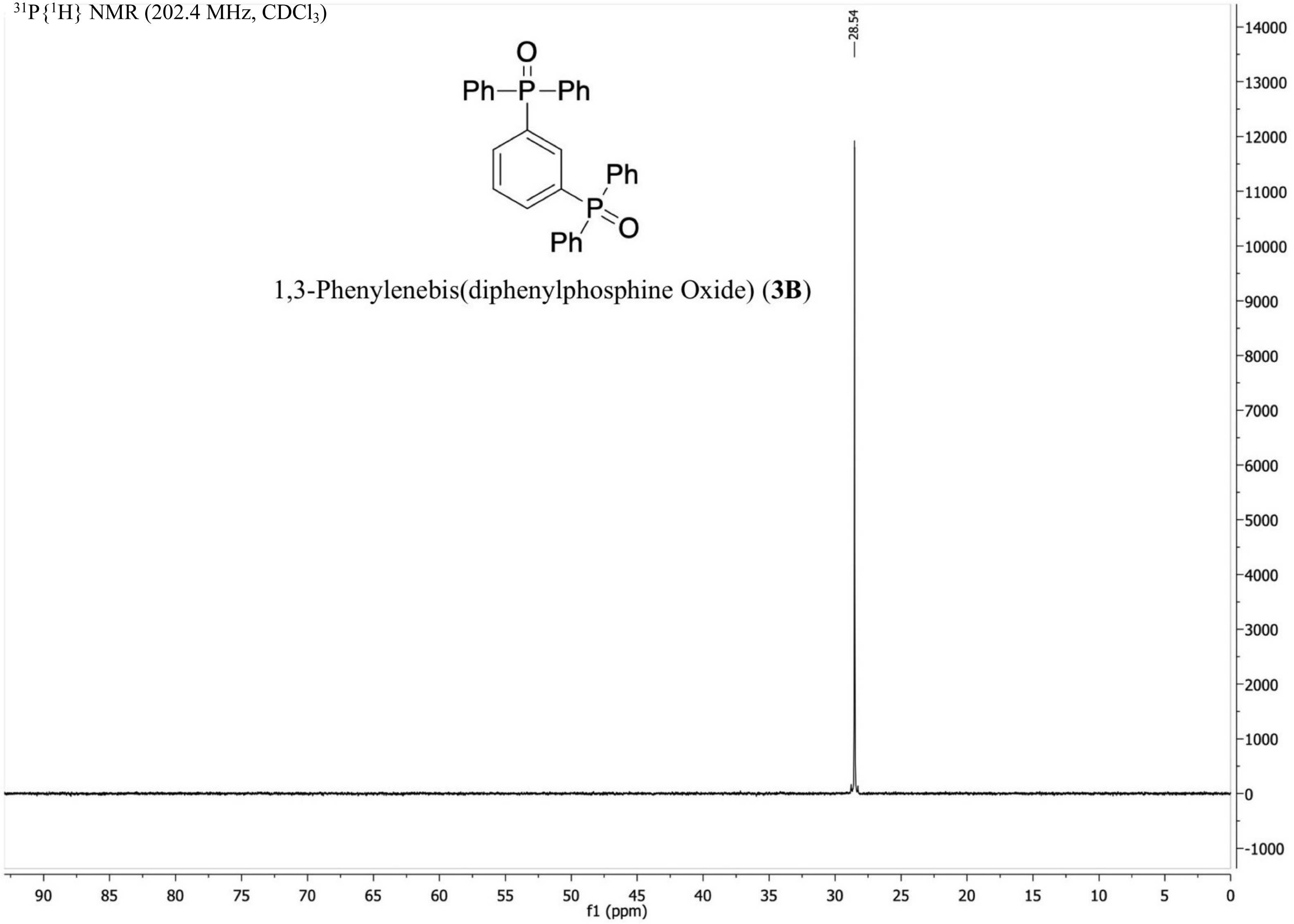

1,3-Phenylenebis(diphenylphosphine Oxide) (**3B**)

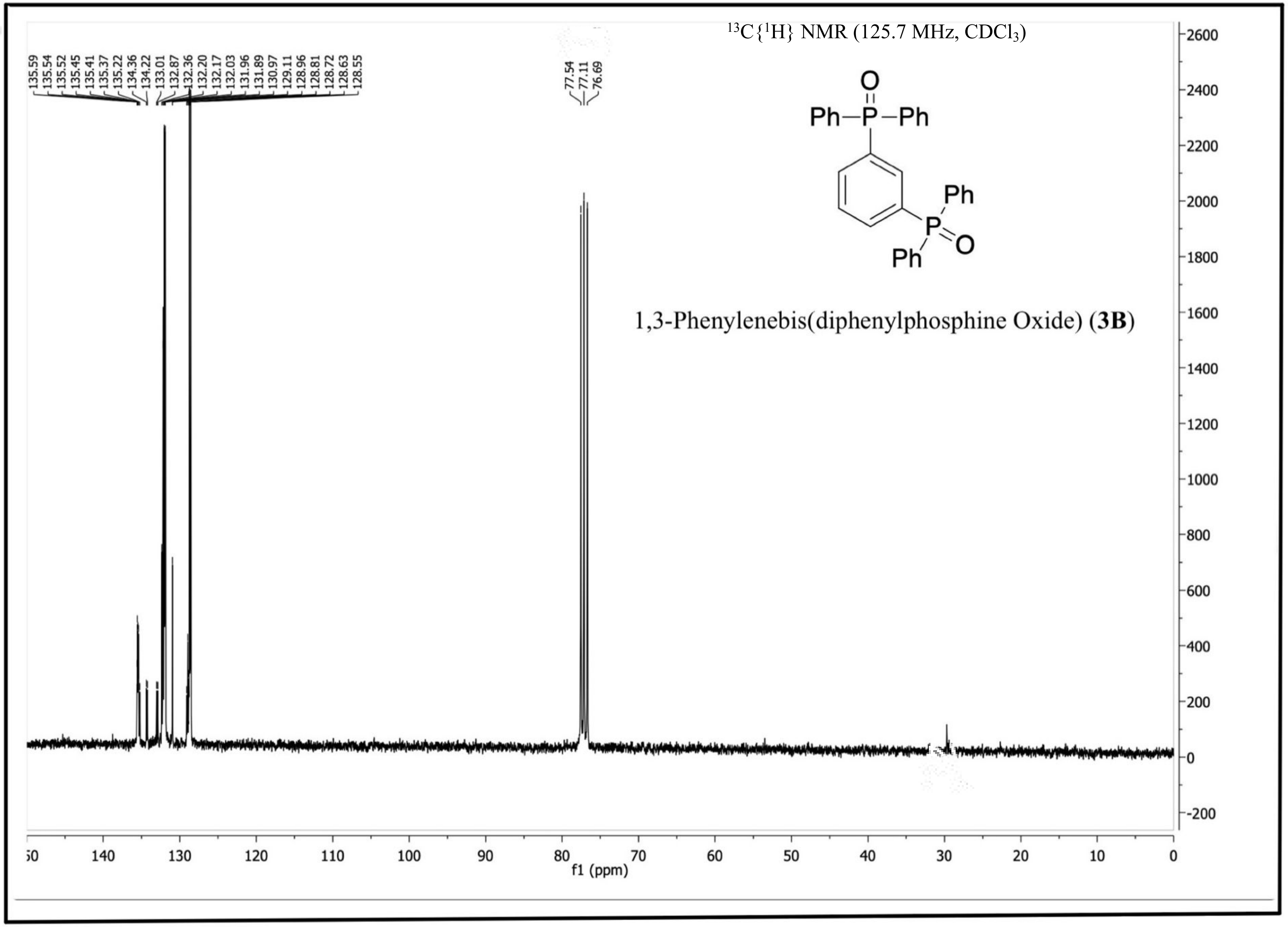

13C{1H} NMR (125.7 MHz, CDCl3)
1,3-Phenylenebis(diphenylphosphine Oxide) (3B)
Ph
P
O
135.59
135.54
135.52
135.45
135.41
135.37
135.22
134.36
134.22
133.01
132.87
132.36
132.20
132.17
132.03
131.96
131.89
130.97
129.11
128.96
128.81
128.72
128.63
128.55
77.54
77.11
76.69
f1 (ppm)

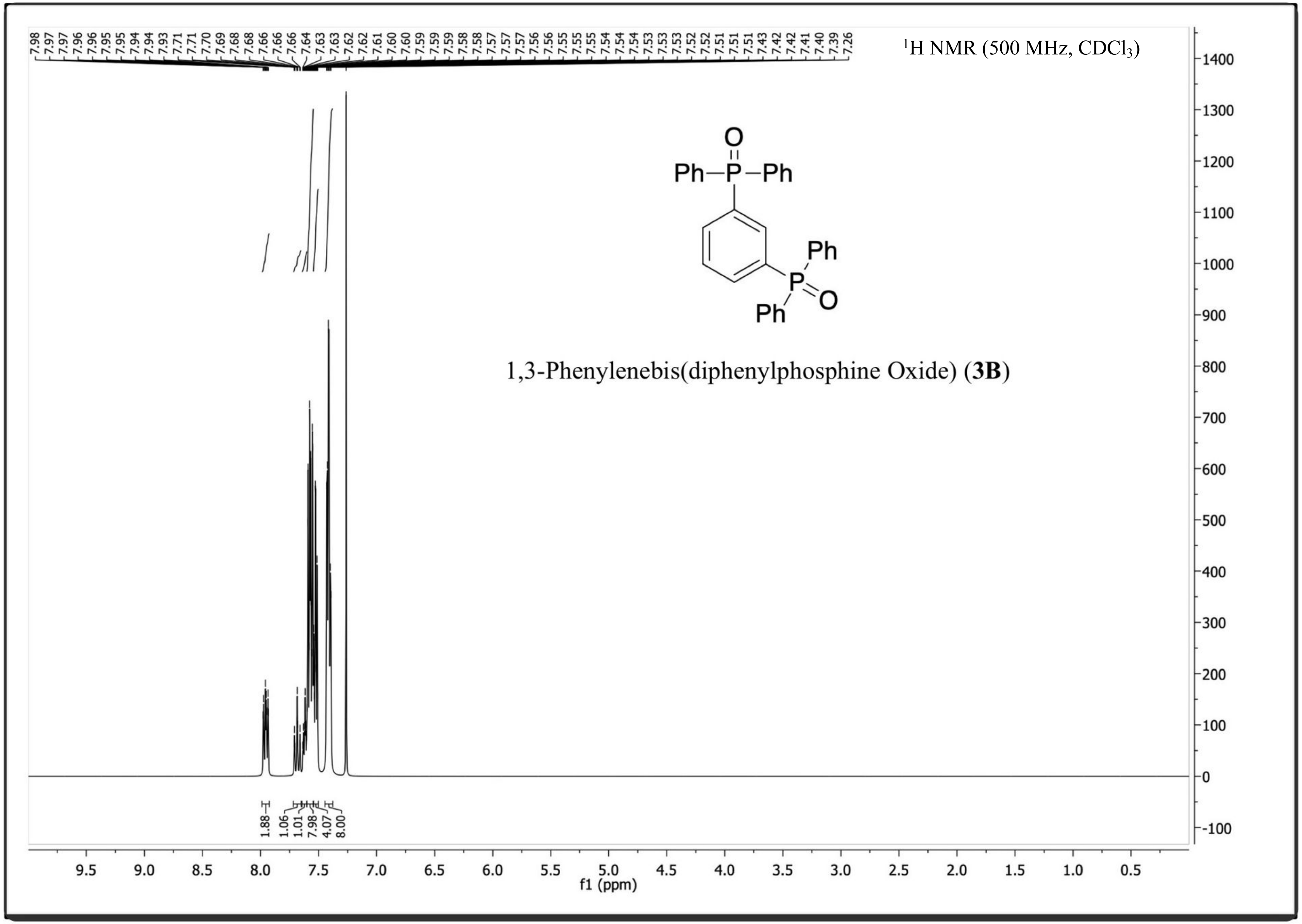

^{1}H NMR (500 MHz, CDCl$_3$)
1,3-Phenylenebis(diphenylphosphine Oxide) (**3B**)
Ph
P
O
f1 (ppm)

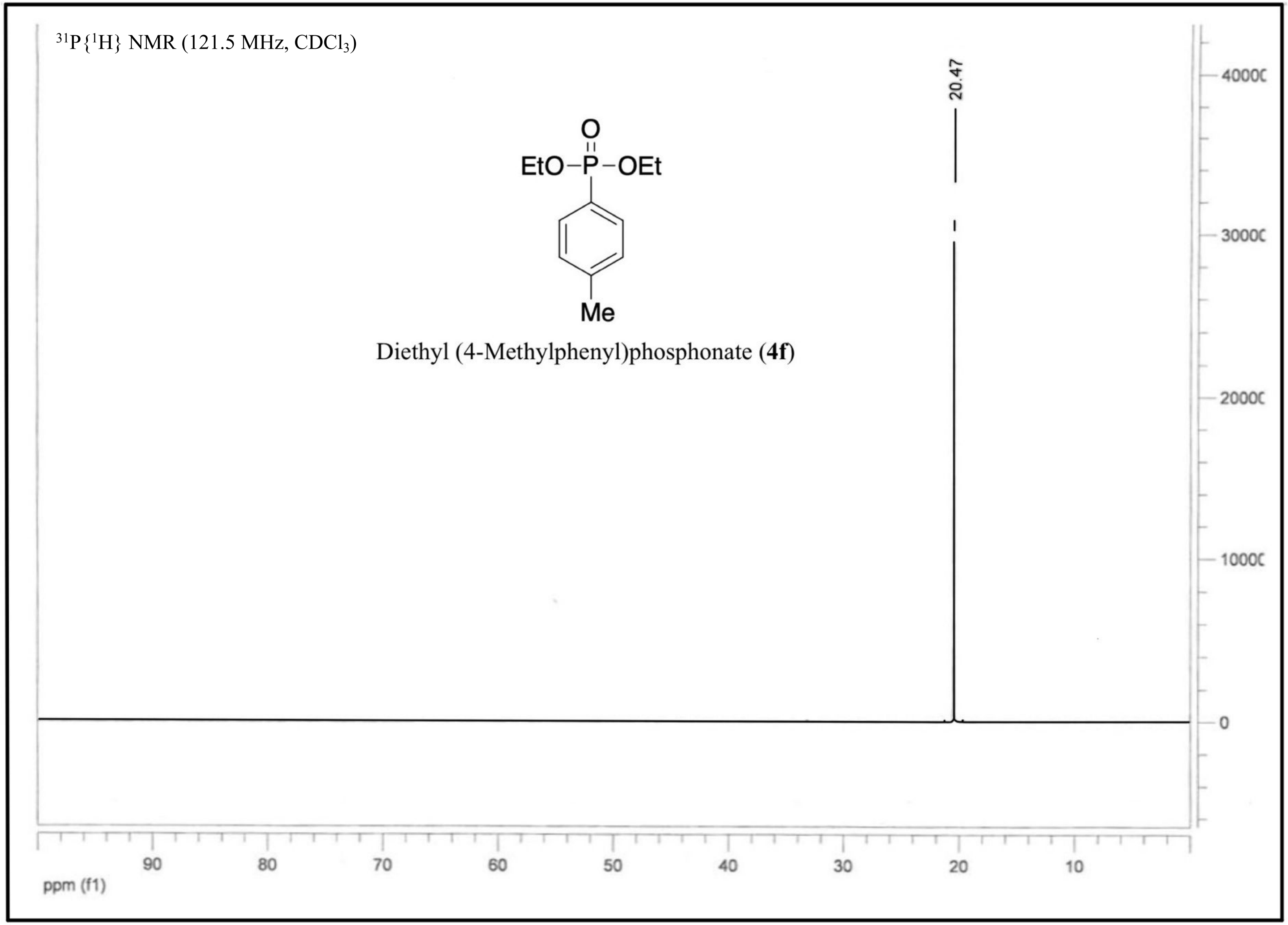

31P{1H} NMR (121.5 MHz, CDCl3)
20.47
EtO
P
OEt
O
Me
Diethyl (4-Methylphenyl)phosphonate (4f)
ppm (f1)
90
80
70
60
50
40
30
20
10
40000
30000
20000
10000
0

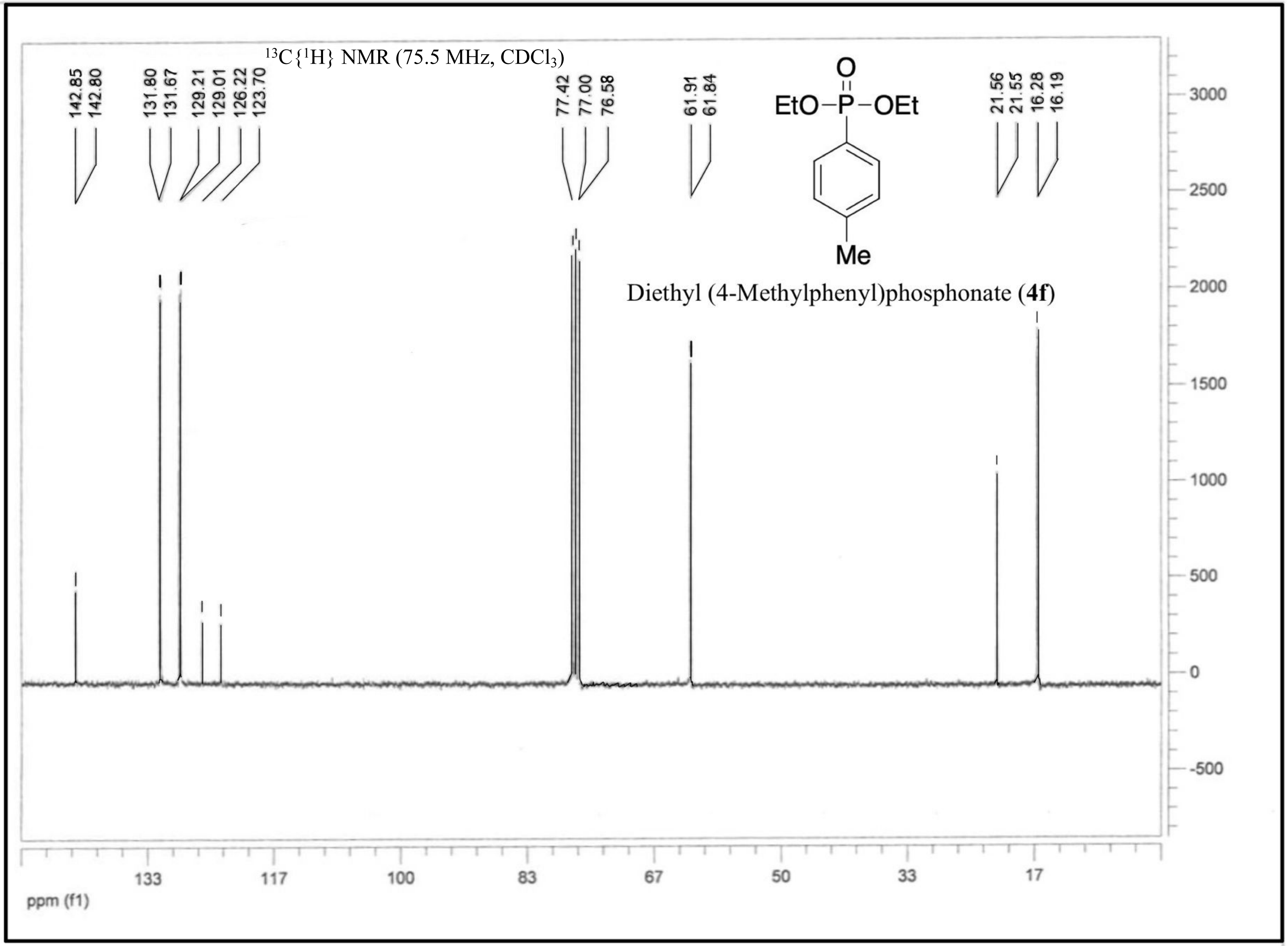

Diethyl (4-Methylphenyl)phosphonate (**4f**)

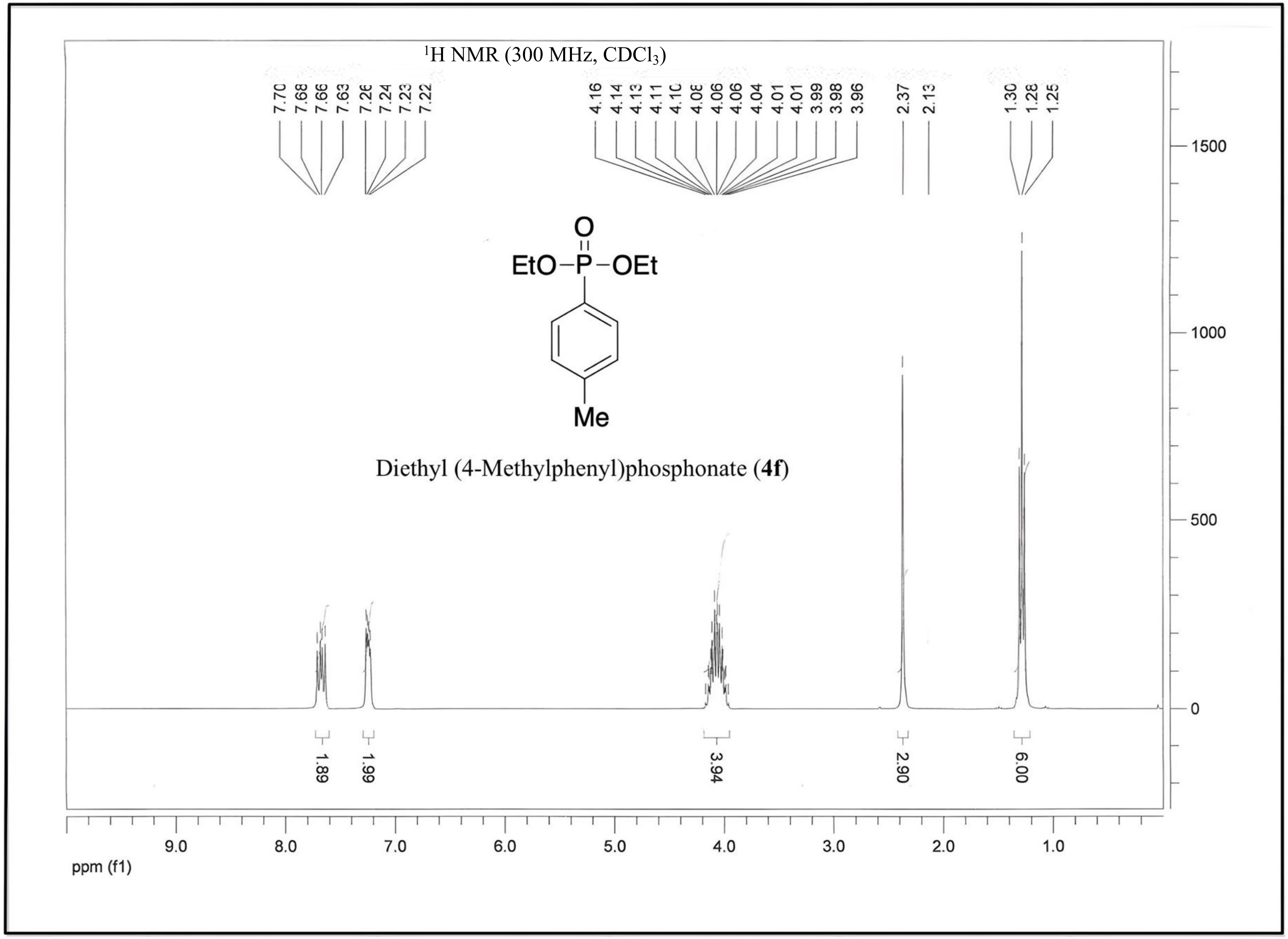
1H NMR (300 MHz, CDCl3)
7.70 7.68 7.66 7.63 7.26 7.24 7.23 7.22
4.16 4.14 4.13 4.11 4.10 4.08 4.06 4.06 4.04 4.01 4.01 3.99 3.98 3.96
2.37 2.13
1.30 1.28 1.25
EtO P OEt
O
Me
Diethyl (4-Methylphenyl)phosphonate (4f)
1500
1000
500
0
1.89 1.99 3.94 2.90 6.00
9.0 8.0 7.0 6.0 5.0 4.0 3.0 2.0 1.0
ppm (f1)

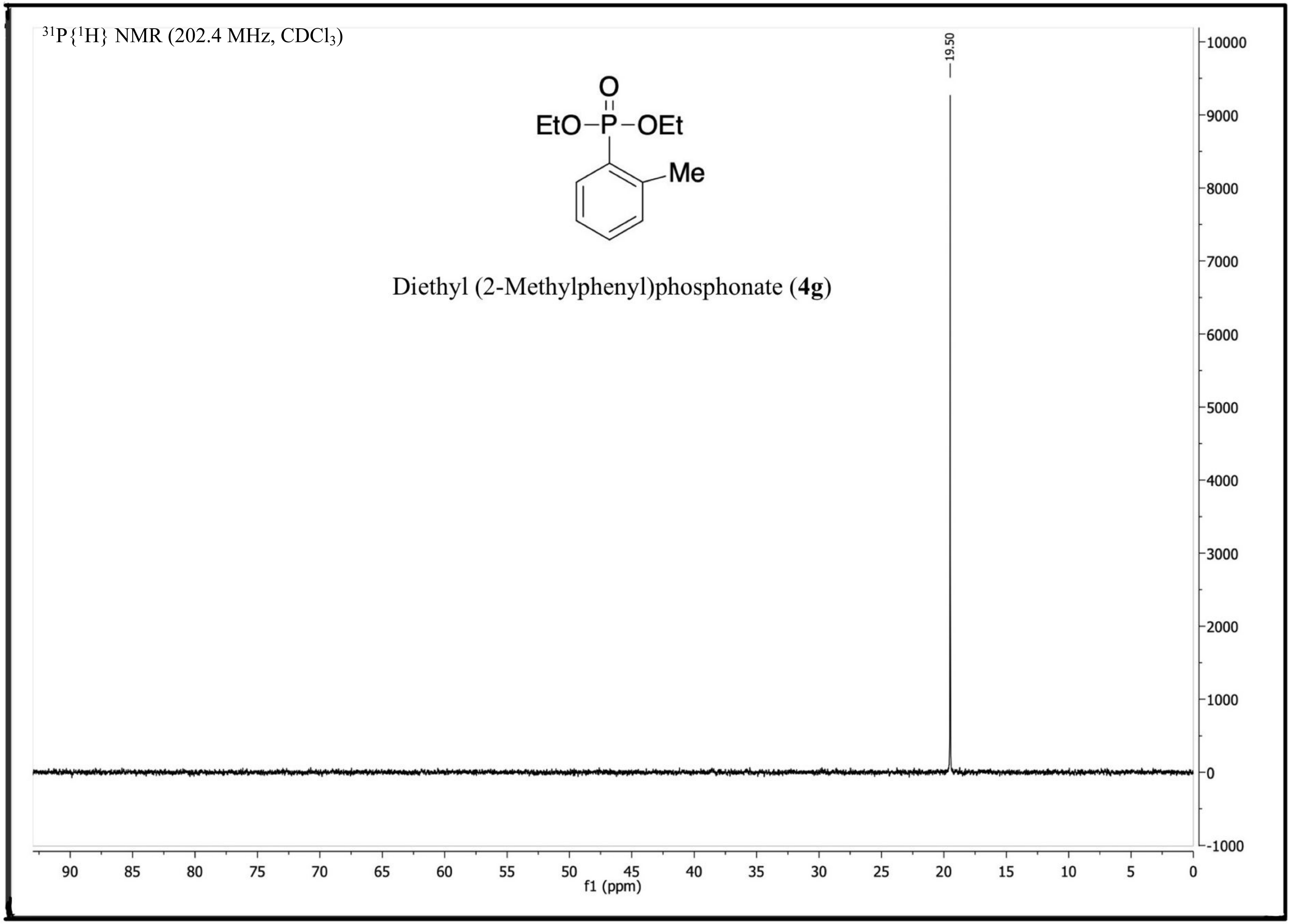
31P{1H} NMR (202.4 MHz, CDCl3)
19.50
EtO–P–OEt
O
Me
Diethyl (2-Methylphenyl)phosphonate (4g)
f1 (ppm)

$^{13}C\{^{1}H\}$ NMR (125.7 MHz, $CDCl_3$)

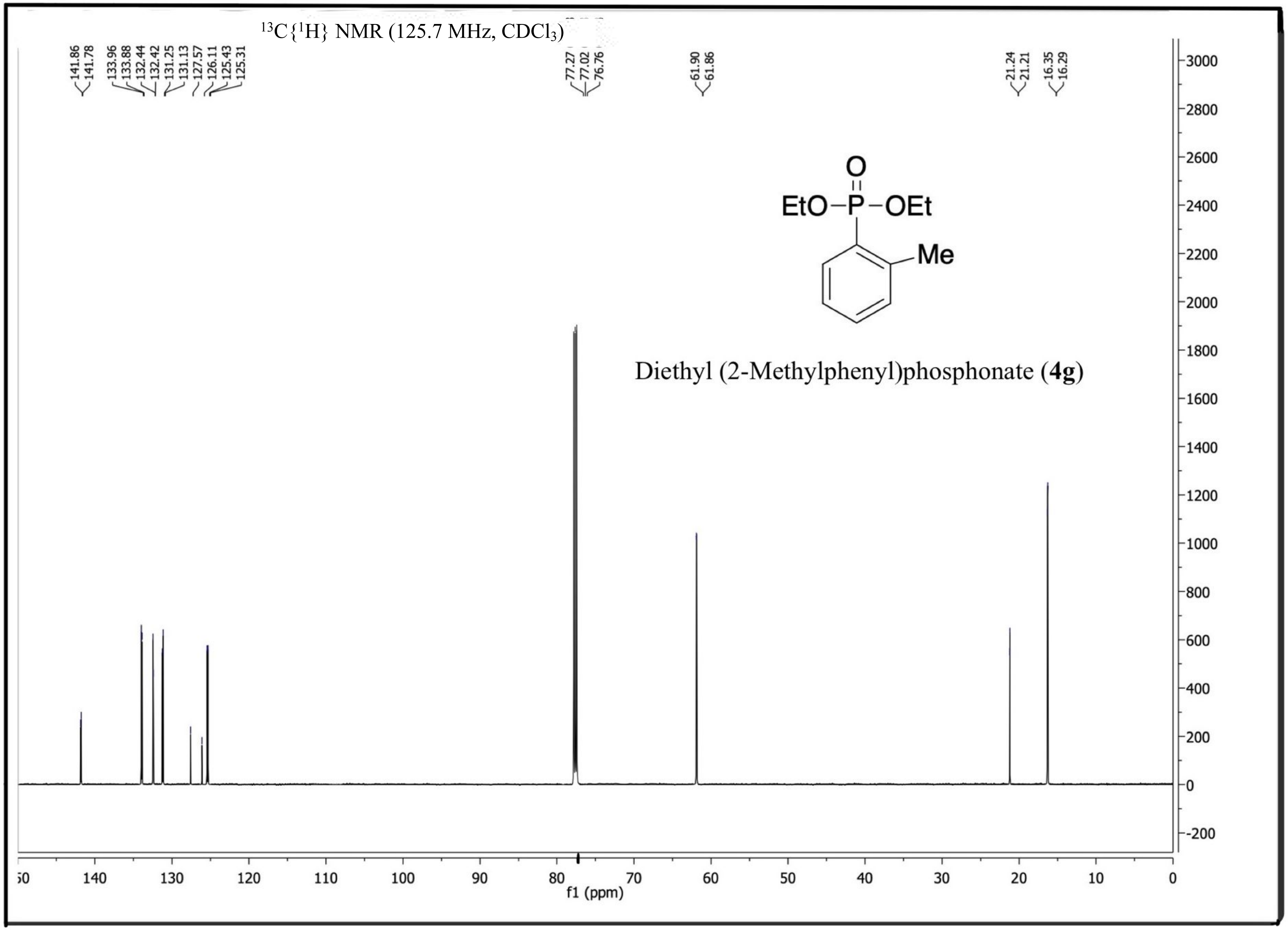

Diethyl (2-Methylphenyl)phosphonate (**4g**)

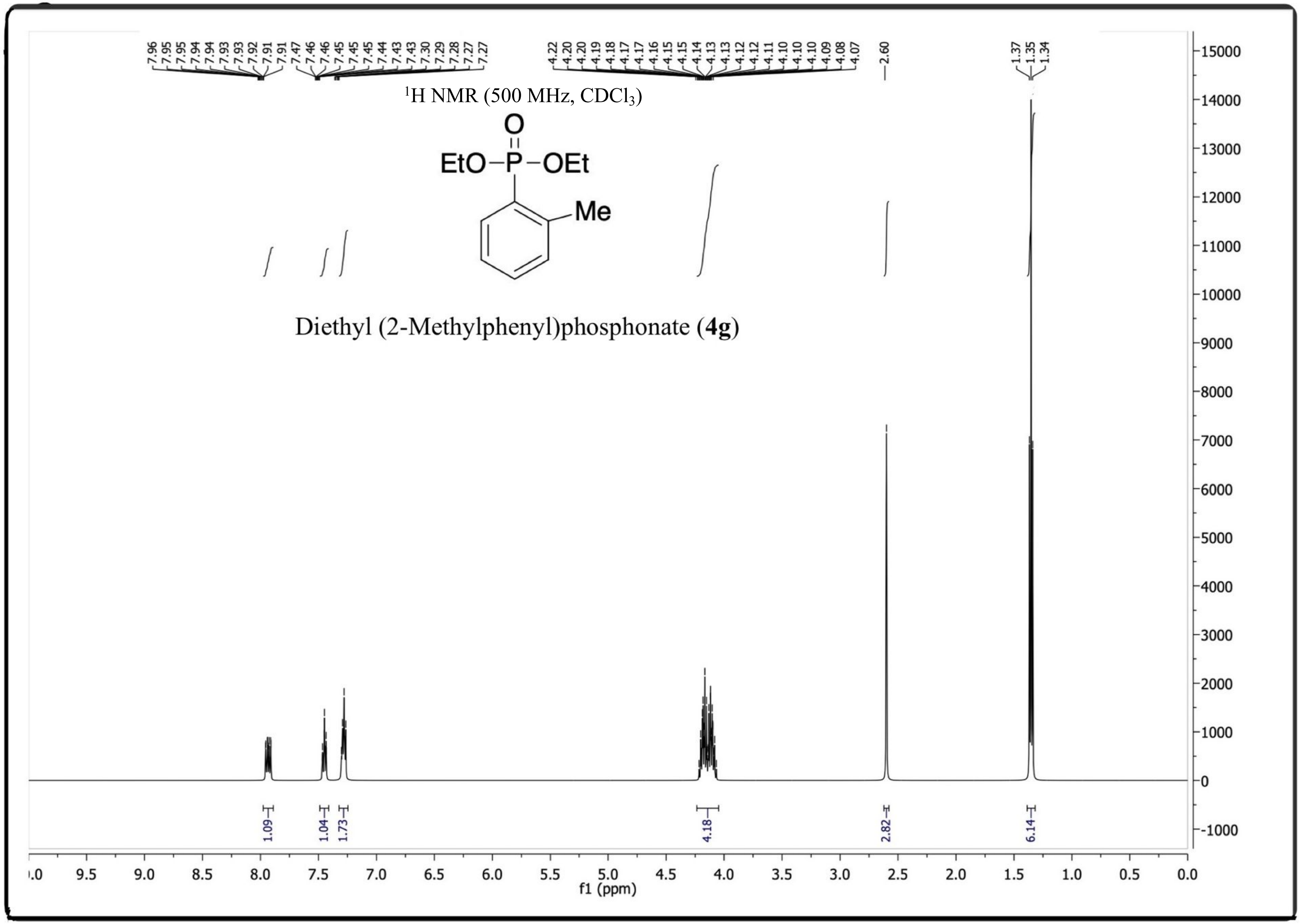

7.96
7.95
7.95
7.94
7.94
7.93
7.93
7.92
7.91
7.91
7.47
7.46
7.46
7.45
7.45
7.45
7.44
7.43
7.43
7.30
7.29
7.28
7.27
7.27
4.22
4.20
4.20
4.19
4.18
4.17
4.17
4.16
4.15
4.15
4.14
4.13
4.13
4.12
4.12
4.11
4.10
4.10
4.10
4.09
4.08
4.07
2.60
1.37
1.35
1.34
¹H NMR (500 MHz, CDCl₃)
O
EtO
P
OEt
Me
Diethyl (2-Methylphenyl)phosphonate (4g)
1.09
1.04
1.73
4.18
2.82
6.14
f1 (ppm)

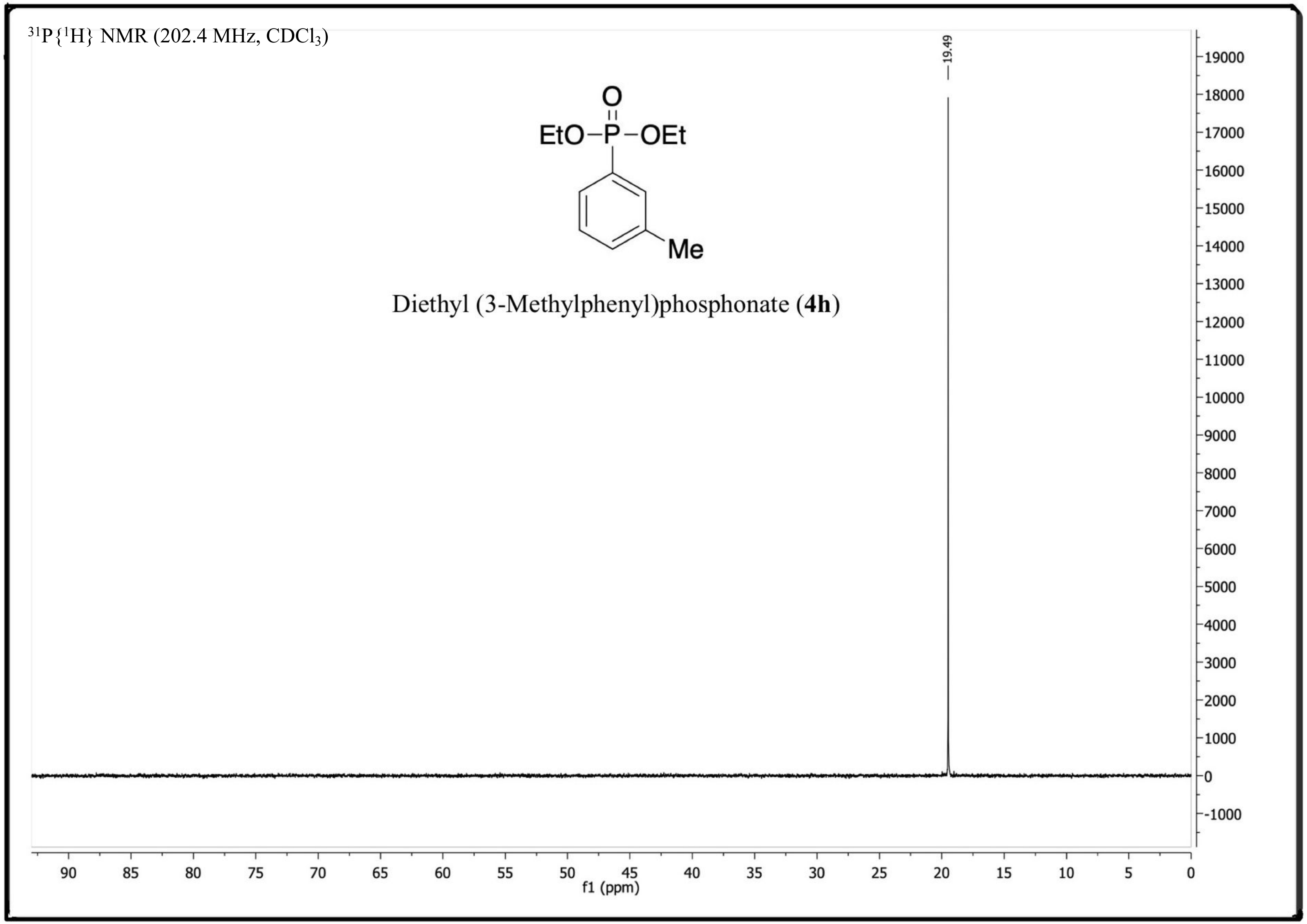
$^{31}P\{^1H\}$ NMR (202.4 MHz, $CDCl_3$)
19.49
EtO
OEt
O
P
Me
Diethyl (3-Methylphenyl)phosphonate (**4h**)
f1 (ppm)

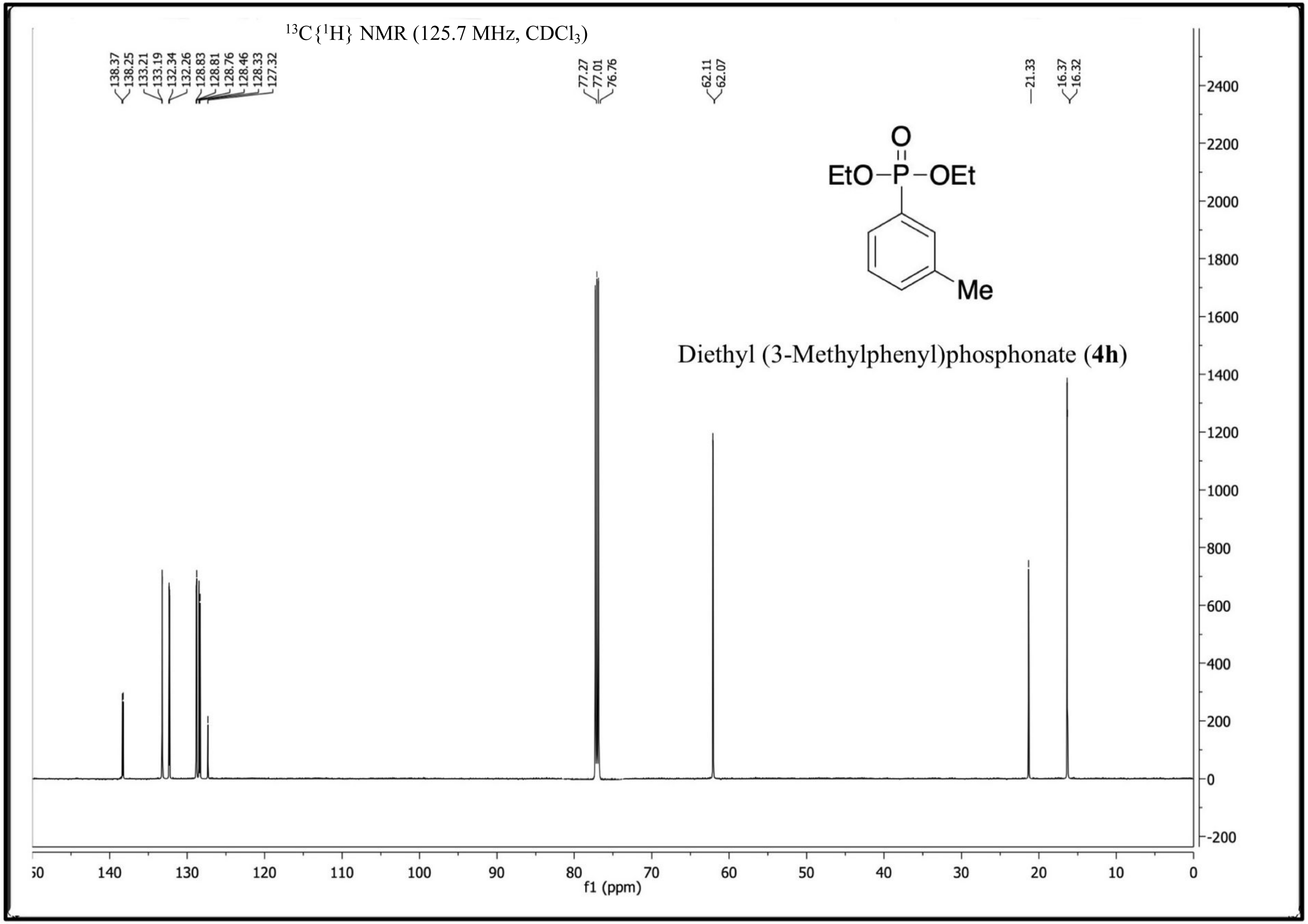

$^{13}C\{^1H\}$ NMR (125.7 MHz, CDCl$_3$)
138.37
138.25
133.21
133.19
132.34
132.26
128.83
128.81
128.76
128.46
128.33
127.32
77.27
77.01
76.76
62.11
62.07
21.33
16.37
16.32
EtO
P
OEt
O
Me
Diethyl (3-Methylphenyl)phosphonate (**4h**)
f1 (ppm)

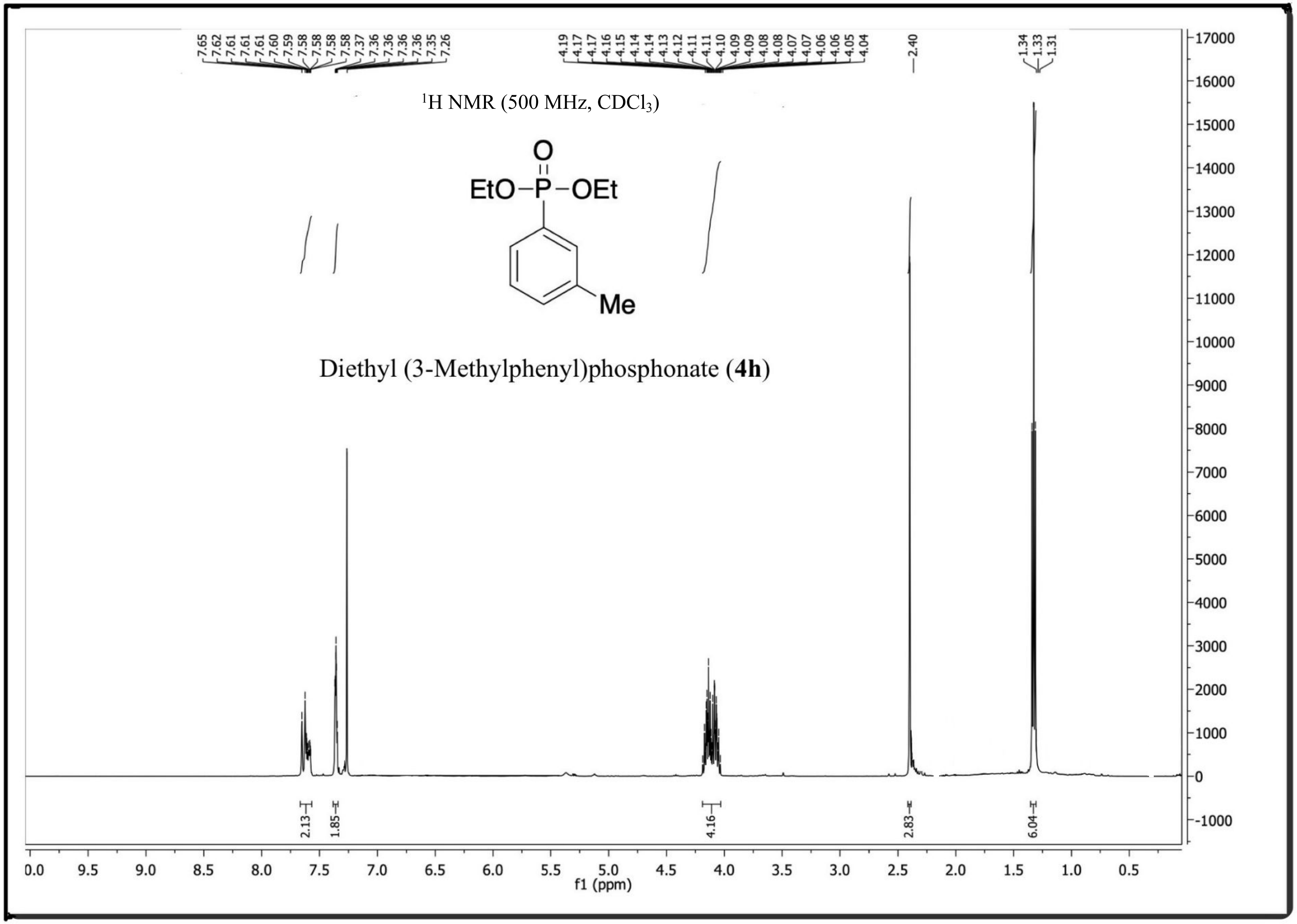
7.65
7.62
7.61
7.61
7.61
7.60
7.59
7.58
7.58
7.58
7.58
7.37
7.36
7.36
7.36
7.36
7.35
7.26
4.19
4.17
4.17
4.16
4.15
4.14
4.14
4.13
4.12
4.11
4.11
4.10
4.09
4.09
4.08
4.08
4.07
4.07
4.06
4.06
4.05
4.04
2.40
1.34
1.33
1.31
¹H NMR (500 MHz, CDCl₃)
EtO
P
OEt
O
Me
Diethyl (3-Methylphenyl)phosphonate (4h)
2.13
1.85
4.16
2.83
6.04
f1 (ppm)

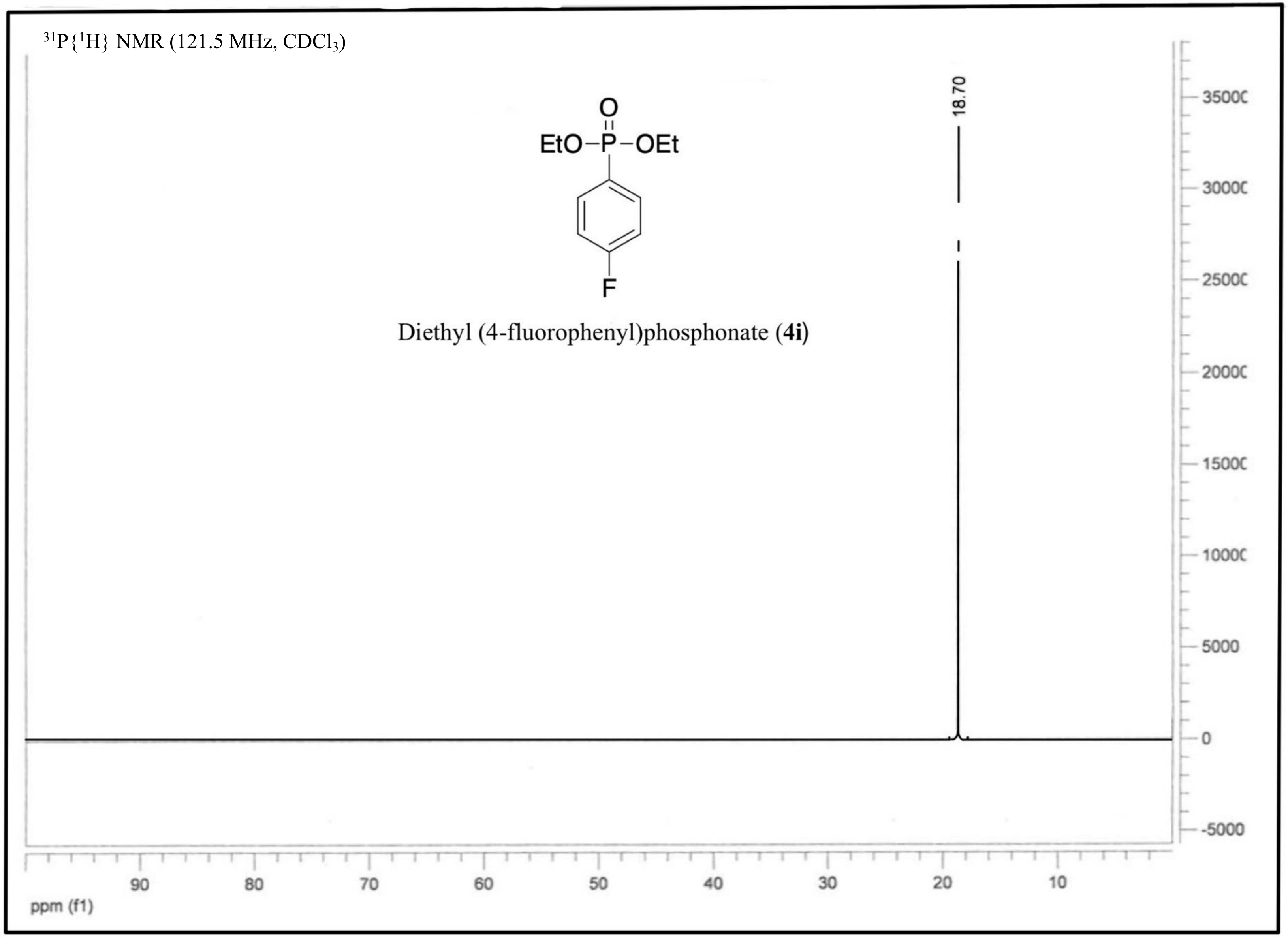
$^{31}P\{^{1}H\}$ NMR (121.5 MHz, $CDCl_3$)
EtO–P–OEt
O
F
Diethyl (4-fluorophenyl)phosphonate (**4i**)
18.70
35000
30000
25000
20000
15000
10000
5000
0
-5000
90
80
70
60
50
40
30
20
10
ppm (f1)

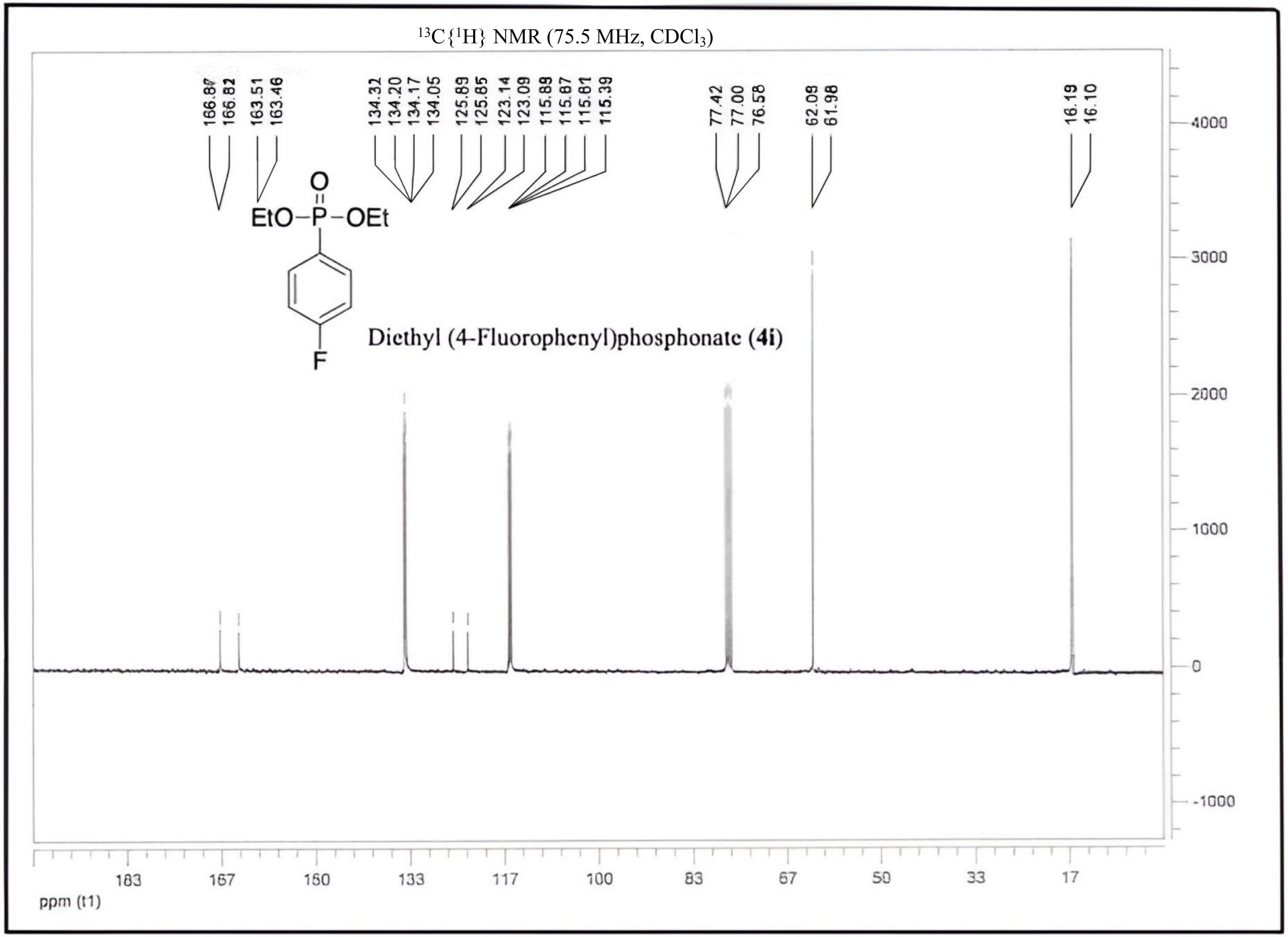
13C{1H} NMR (75.5 MHz, CDCl3)
166.87
166.82
163.51
163.46
134.32
134.20
134.17
134.05
125.89
125.85
123.14
123.09
115.89
115.87
115.81
115.39
77.42
77.00
76.58
62.08
61.98
16.19
16.10
EtO
P
OEt
O
F
Diethyl (4-Fluorophenyl)phosphonate (4i)
4000
3000
2000
1000
0
-1000
183
167
150
133
117
100
83
67
50
33
17
ppm (t1)

^1H NMR (300 MHz, $CDCl_3$)

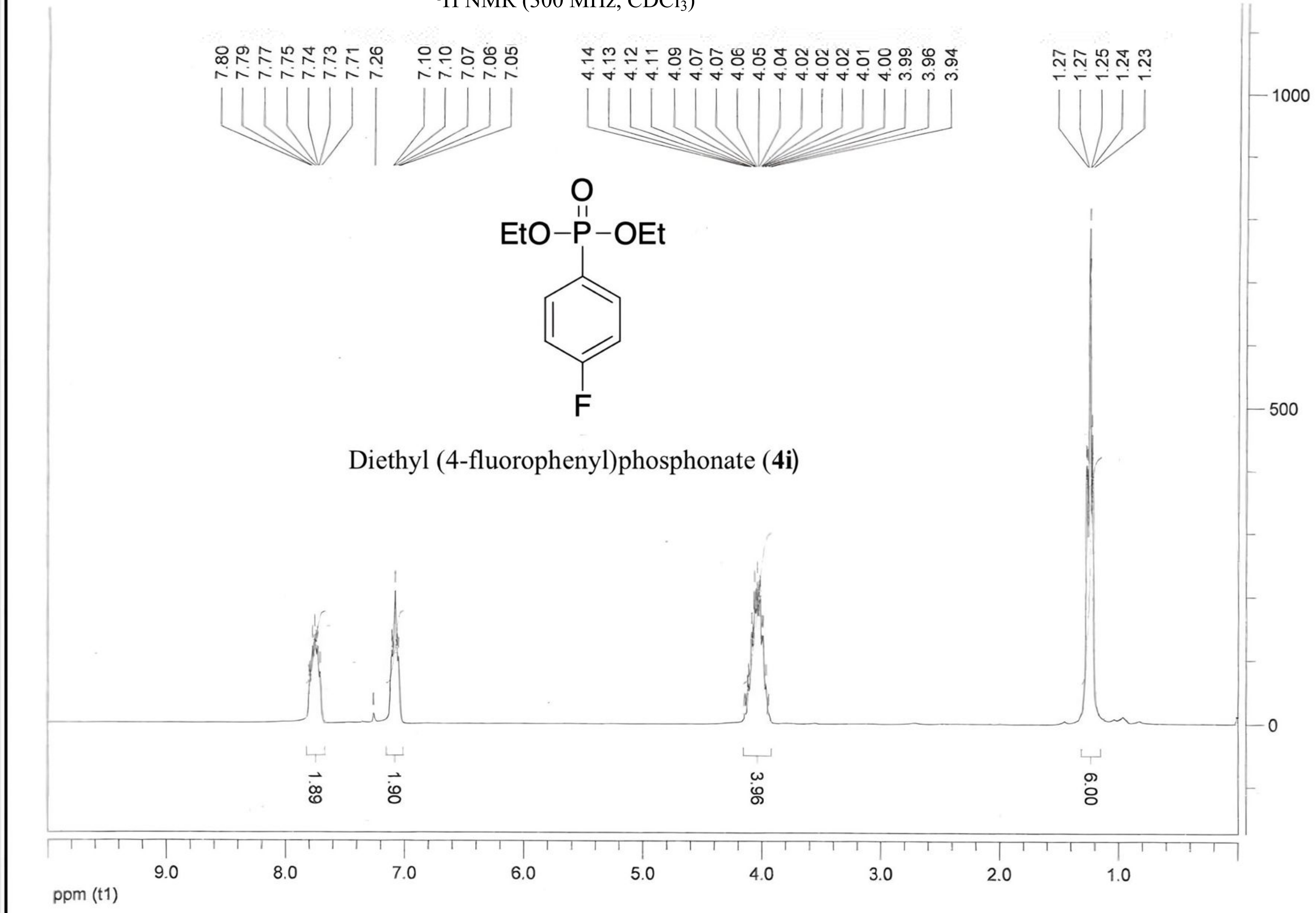

Diethyl (4-fluorophenyl)phosphonate (**4i**)

$^{31}P\{^1H\}$ NMR (121.5 MHz, $CDCl_3$)

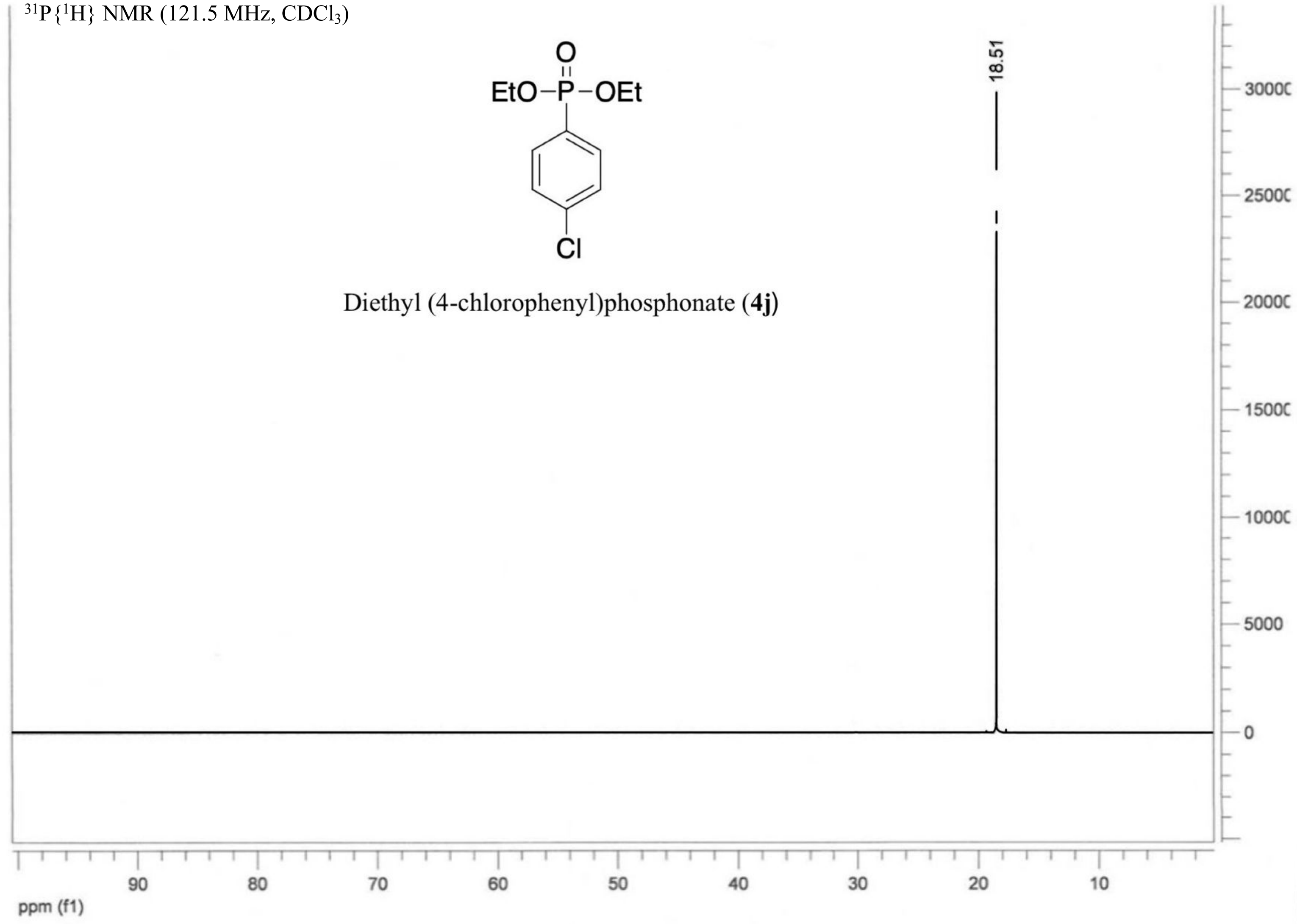

Diethyl (4-chlorophenyl)phosphonate (**4j**)

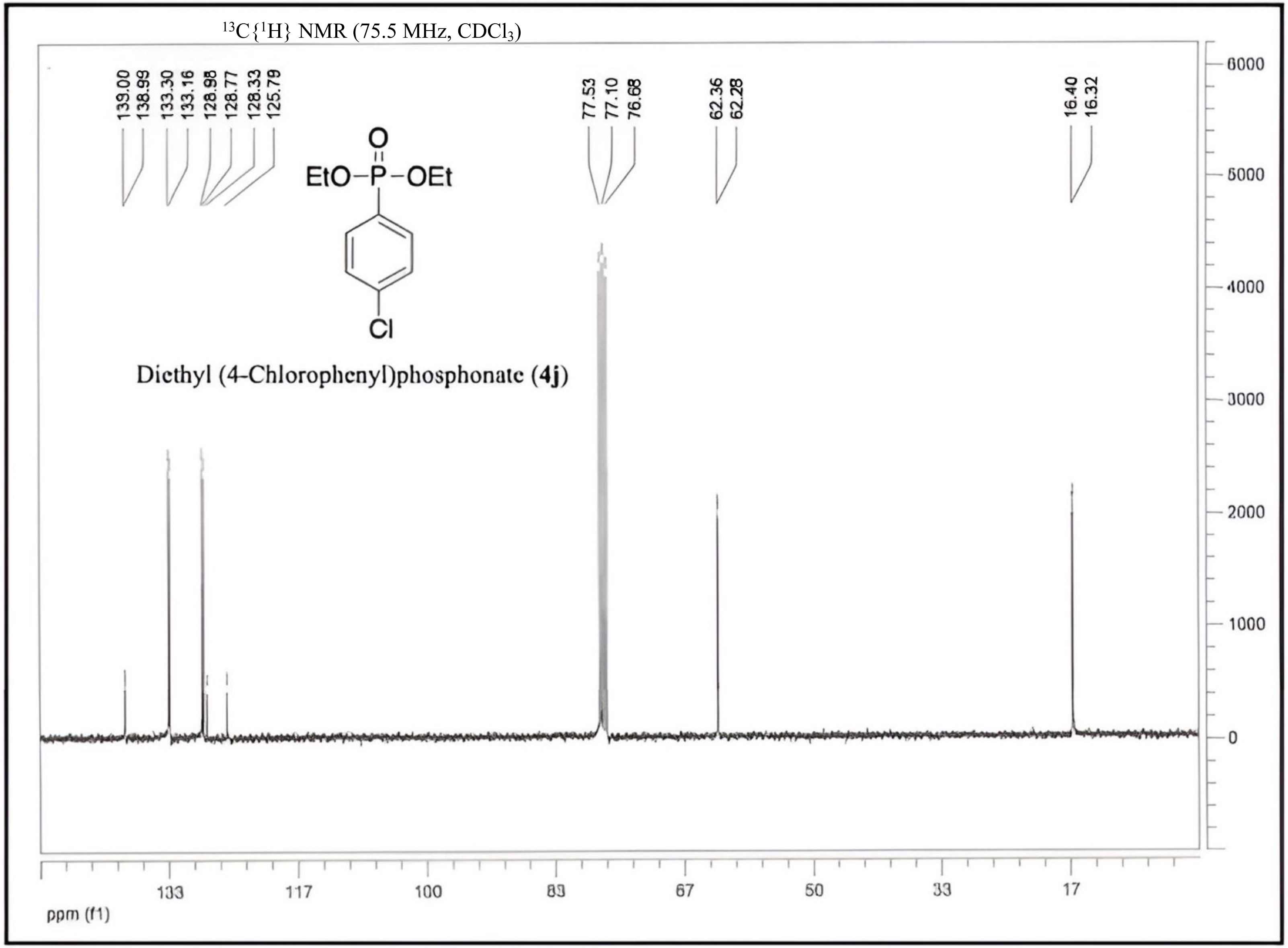

$^{13}C\{^{1}H\}$ NMR (75.5 MHz, $CDCl_3$)
139.00
138.99
133.30
133.16
128.98
128.77
128.33
125.79
77.53
77.10
76.68
62.36
62.28
16.40
16.32
O
EtO–P–OEt
Cl
Diethyl (4-Chlorophenyl)phosphonate (**4j**)
6000
5000
4000
3000
2000
1000
0
133
117
100
83
67
50
33
17
ppm (f1)

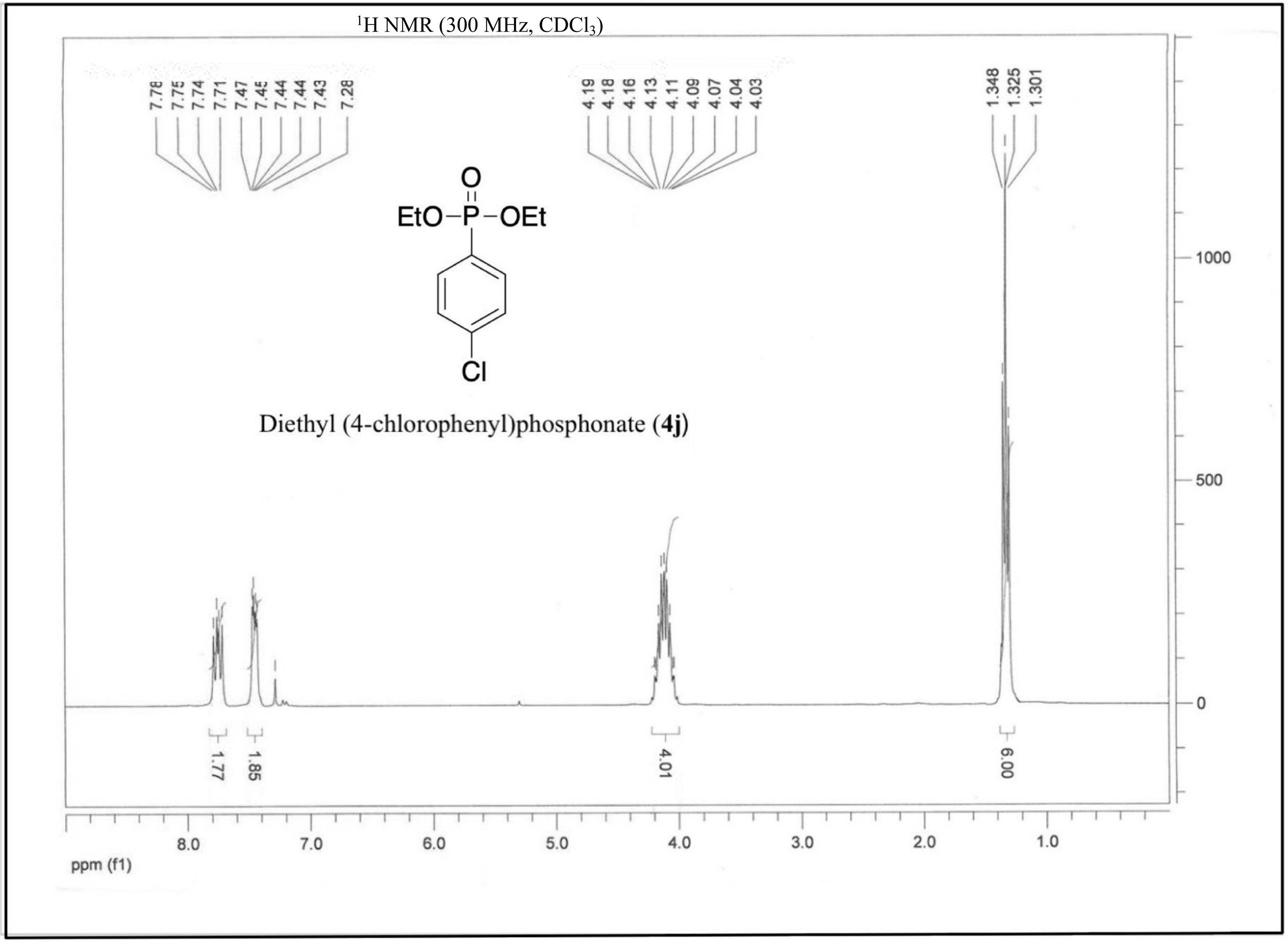
1H NMR (300 MHz, CDCl3)
7.78
7.75
7.74
7.71
7.47
7.45
7.44
7.44
7.43
7.28
4.19
4.18
4.16
4.13
4.11
4.09
4.07
4.04
4.03
1.348
1.325
1.301
O
EtO
P
OEt
Cl
Diethyl (4-chlorophenyl)phosphonate (4j)
1000
500
0
1.77
1.85
4.01
6.00
8.0
7.0
6.0
5.0
4.0
3.0
2.0
1.0
ppm (f1)

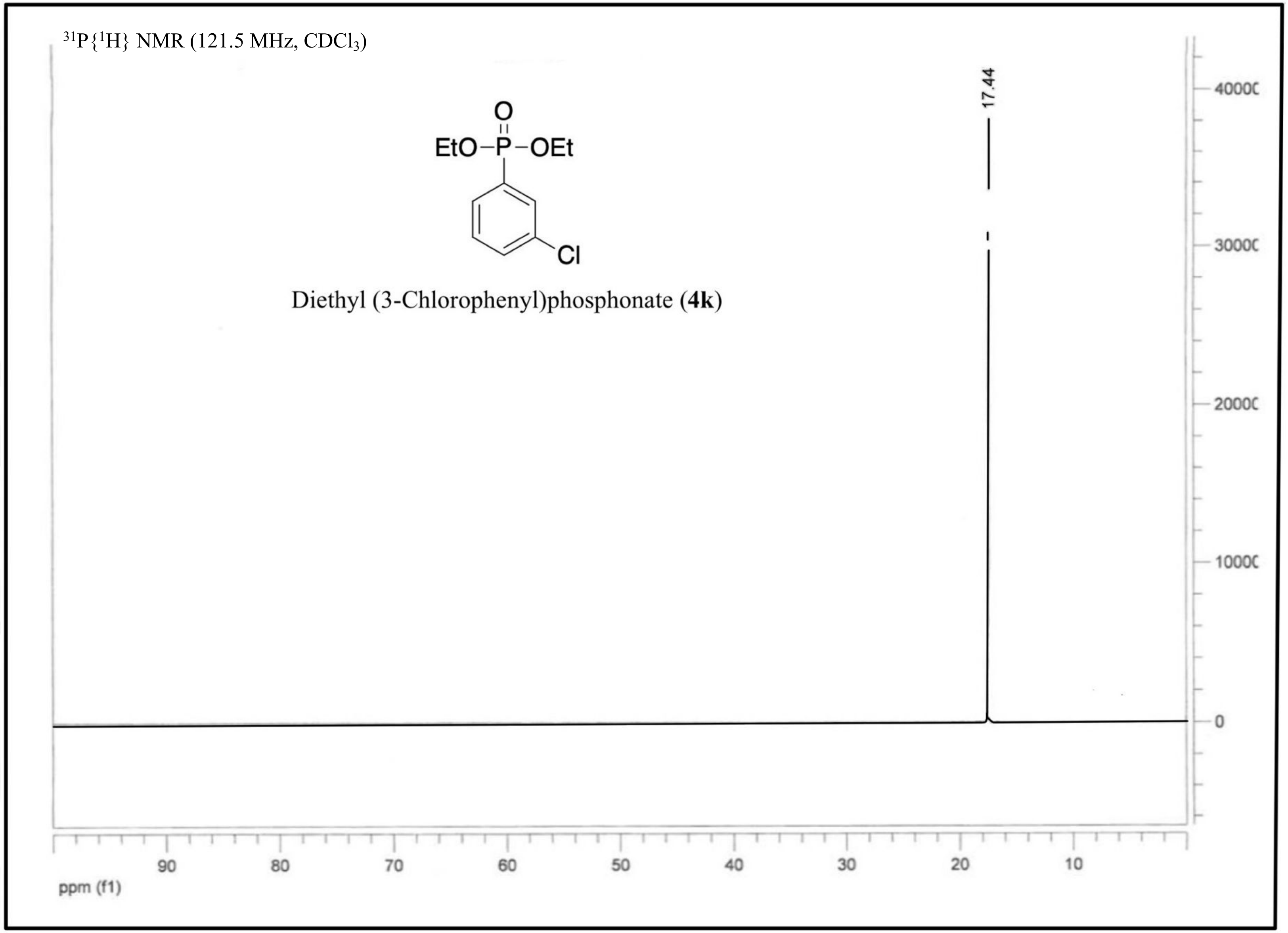

³¹P{¹H} NMR (121.5 MHz, CDCl3)
17.44
Diethyl (3-Chlorophenyl)phosphonate (4k)
ppm (f1)

$^{13}C\{^1H\}$ NMR (125.7 MHz, $CDCl_3$)

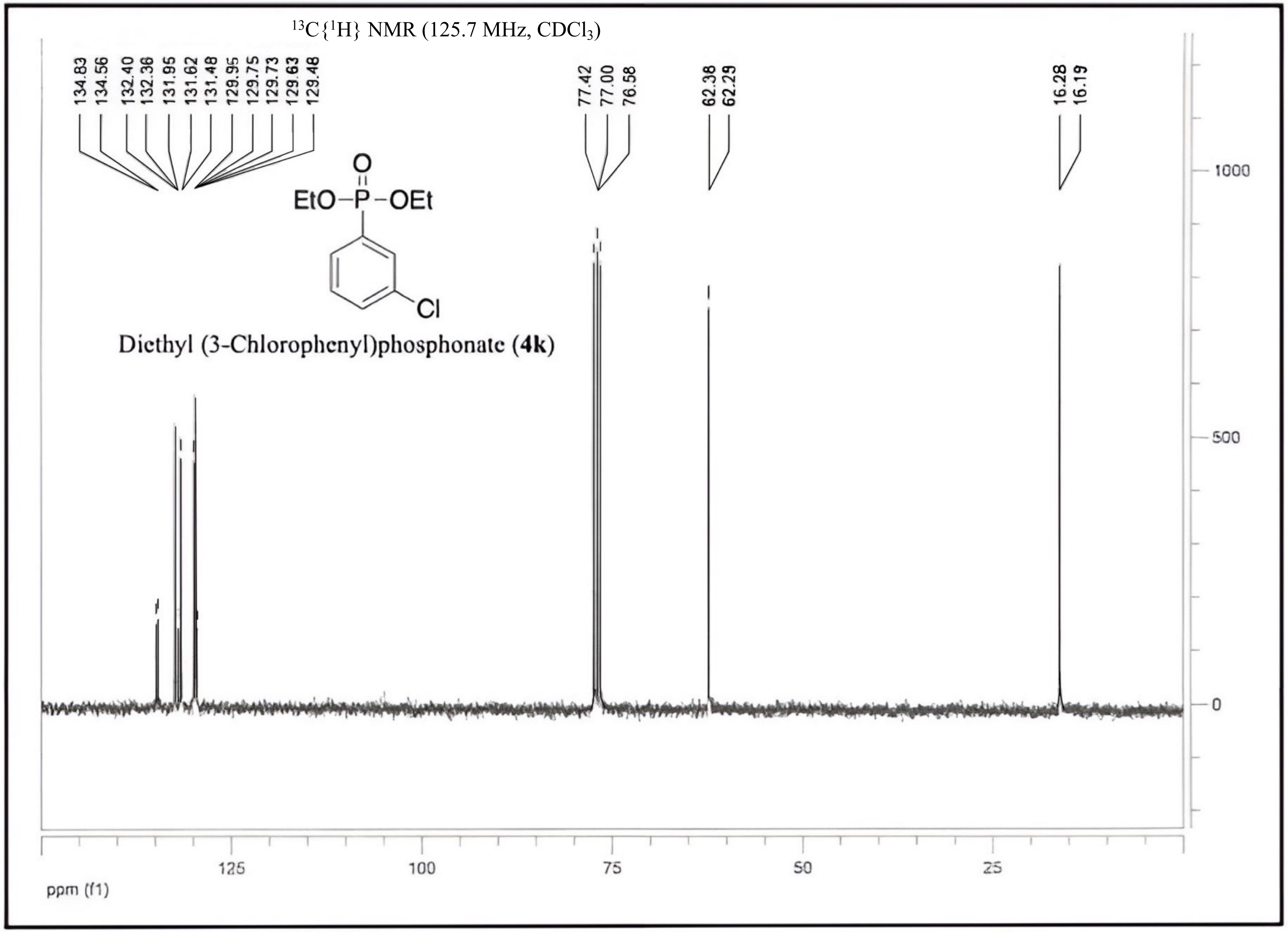

Diethyl (3-Chlorophenyl)phosphonate (**4k**)

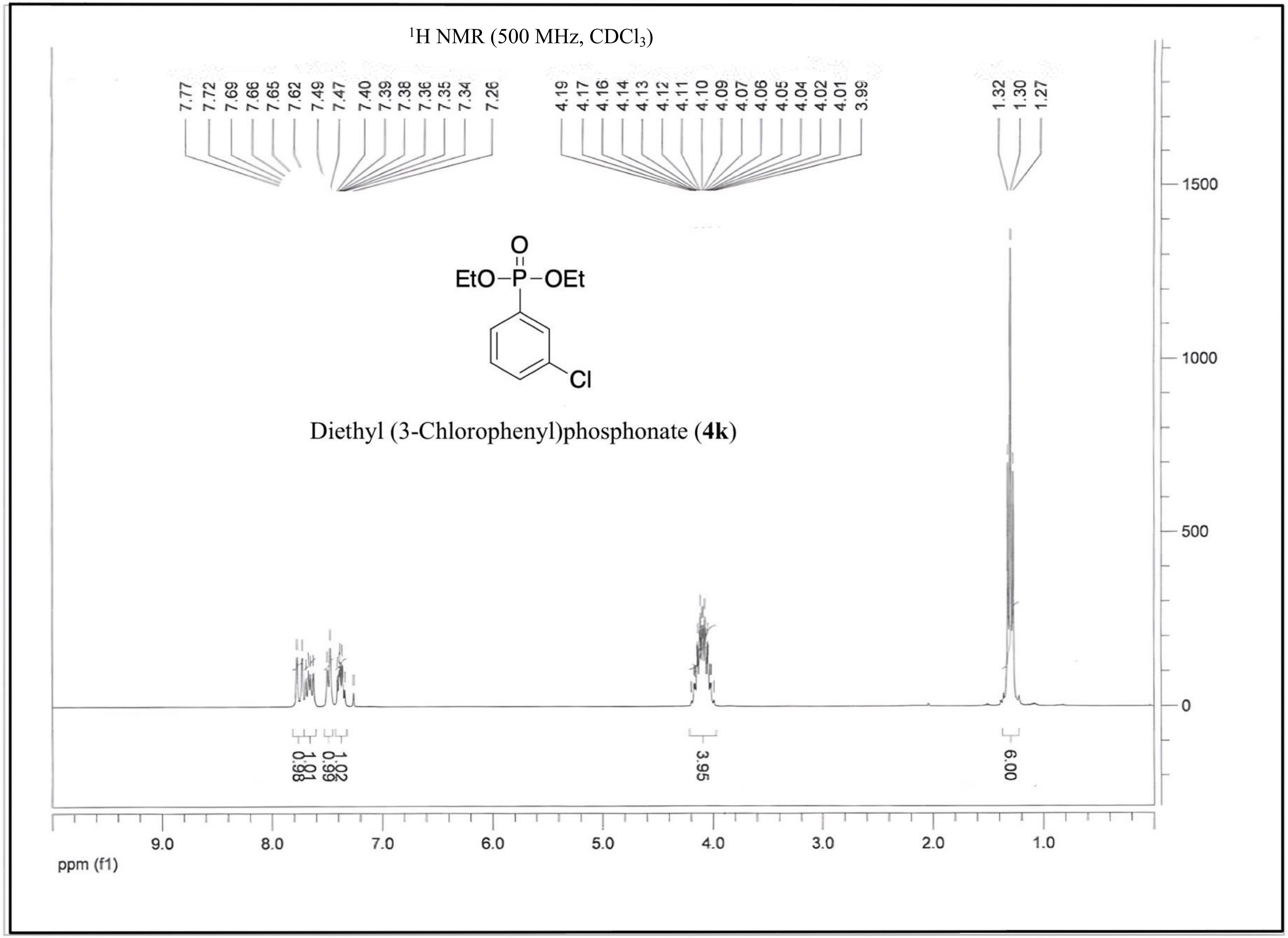

Diethyl (3-Chlorophenyl)phosphonate (**4k**)

Table S1–S3 Containing the Computed Row Data

Table S1. Computed energies (*E*), zero point energies, internal energies (*U*), enthalpies (*H*) and Gibbs free energies (*G*) given in Hartree as well as entropies (*S*) given in J mol^{-1} K^{-1} at M06-2X/6-31G(d,p) basis set with the consideration of PCM solvent method using the parameter set of EtOH for **small molecules and ions**.

Compound	Number	E	ZPE	U	H	G	S
$PhB(OH)_2$		-408.11774237	-407.991673	-407.984636	-407.983692	-408.023130	83.005
$PhB(OH)(O^-)$		-407.61143116	-407.498195	-407.490588	-407.489644	-407.530916	86.866
EtOH dimer		-309.94978630	-309.785488	-309.775914	-309.774970	-309.820640	96.122
H_2O		-76.39100903	-76.369386	-76.366551	-76.365607	-76.387037	45.104
$O{=}B{-}O^-$		-175.46608837	-175.456341	-175.453574	-175.452630	-175.477220	51.754
O=B-OH		-175.92773828	-175.907372	-175.903965	-175.903021	-175.930620	58.087
$B(OH)_2(O^-)$		-251.89209748	-251.855581	-251.851413	-251.850469	-251.881877	66.104
$B(OH_{)3}$		-252.40593421	-252.356721	-252.352280	-252.351336	-252.383183	67.028
HCO_3^-		-729.40162094	-729.125144	-729.105768	-729.104824	-729.176135	150.088
CO_3^{2-}		-728.88486694	-728.624652	-728.607295	-728.606351	-728.674444	143.315
AcOH+2EtOH		-538.97381352	-538.745097	-538.730086	-538.729142	-538.789680	127.414
AcO^-+2EtOH		-538.48575818	-538.271219	-538.255991	-538.255047	-538.316606	129.562
$Pd(OAc)_2$		-584.63236442	-584.528750	-584.519524	-584.518580	-584.564883	97.453
Ph_2PHO		-880.24105842	-880.042526	-880.030705	-880.029760	-880.082735	111.495
Ph_2POH		-880.23823639	-880.039449	-880.027104	-880.026160	-880.080233	113.805
Ph_2POH		-880.23885591	-880.039879	-880.027696	-880.026752	-880.079921	111.903
PPh_2O^-		-1760.01598506	-1759.631825	-1759.606372	-1759.605428	-1759.694760	188.016
$Ph_3P{=}O$	**1a**	-1111.21736487	-1110.936096	-1110.919465	-1110.918521	-1110.983040	135.791
Ph-P=O		-648.05565886	-647.960286	-647.953460	-647.952516	-647.992109	83.330
$Ph{-}P{=}OH^+$		-648.46915594	-648.361576	-648.354544	-648.353600	-648.393411	83.790

Table S2. Computed energies (*E*), zero point energies, internal energies (*U*), enthalpies (*H*) and Gibbs free energies (*G*) given in Hartree as well as entropies (*S*) given in J mol^{-1} K^{-1} at M06-2X/6-31G(d,p) basis set with the consideration of PCM solvent method using the parameter set of EtOH for **Pd complexes**.

Compound	Short	Number	E	ZPE	U	H	G	S
$Pd^{2+}(Ph_2POH)$	$Pd^{2+}[L]$		-1007.70802230	-1007.506290	-1007.492521	-1007.491576	-1007.548762	120.357
$Pd^{2+}(Ph_2POH)_2$	$Pd^{2+}[L_2]$	**18+H$^+$**	-1888.03027080	-1887.626849	-1887.599790	-1887.598846	-1887.685921	183.266
$Pd^{2+}(Ph_2POH)_3$	$Pd^{2+}[L_3]$	**6**	-2768.28121956	-2767.676224	-2767.635729	-2767.634785	-2767.751593	245.844
$Pd^{2+}(Ph_2POH)_4$	$Pd^{2+}[L_4]$	**7**	-3648.60422754	-3647.796707	-3647.743574	-3647.742630	-3647.884022	297.584
$Pd^{2+}(Ph_2POH)(Ph_2PO^-)$	$Pd^{2+}[Q]$	**18**	-1887.61771746	-1887.227014	-1887.200780	-1887.199836	-1887.285826	180.981
$Pd^{2+}(Ph_2POH)_2(Ph_2PO^-)$	$Pd^{2+}[LQ]$	**9**	-2767.86645530	-2767.274564	-2767.234616	-2767.233672	-2767.349855	244.528
$Pd^{2+}(Ph_2POH)_2(Ph_2PO^-)_2$	$Pd^{2+}[Q_2]$	**10**	-3647.74383768	-3646.963519	-3646.911270	-3646.910325	-3647.052038	298.260
$Pd^{2+}(Ph_2POH)_2AcO^-$	$Pd^{2+}[L_2(AcO^-)]$	**5**	-2116.61742344	-2116.162407	-2116.130753	-2116.129809	-2116.228011	206.684
$Pd^{2+}(Ph_2POH)(Ph_2PO^-)AcO^-$	$Pd^{2+}[Q(AcO^-)]$	**8**	-2116.18913352	-2115.747660	-2115.715828	-2115.714883	-2115.817375	215.711

Table S3. Computed energies (*E*), zero point energies, internal energies (*U*), enthalpies (*H*) and Gibbs free energies (*G*) given in Hartree as well as entropies (*S*) given in J mol^{-1} K^{-1} at M06-2X/6-31G(d,p) basis set with the consideration of PCM solvent method using the parameter set of EtOH for **intermediates**.

Number	E	ZPE	U	H	G	S
**11+H$^+$**	-2295.71667401	-2295.197379	-2295.161449	-2295.160504	-2295.269377	229.141
**11**	-2295.29227070	-2294.786277	-2294.751208	-2294.750264	-2294.857292	225.260
**12**	-2295.24345200	-2294.741700	-2294.706200	-2294.705300	-2294.813000	226.758
**13**	-2295.25005729	-2294.747039	-2294.710708	-2294.709764	-2294.821055	234.232
**14**	-2119.29162526	-2118.810757	-2118.778581	-2118.777637	-2118.879178	213.712
**15**	-2999.57484775	-2998.894074	-2998.848471	-2998.847527	-2998.977054	272.613
**16**	-2999.54192300	-2998.861331	-2998.816023	-2998.815079	-2998.944626	272.655
**17**	-2999.58712725	-2998.904671	-2998.858326	-2998.857382	-2998.992671	284.740
**18**	-1887.61771746	-1887.227014	-1887.200780	-1887.199836	-1887.285826	180.981
**9**	-2767.86645530	-2767.274564	-2767.234616	-2767.233672	-2767.349855	244.528
**19**	-2767.77548700	-2767.184927	-2767.145630	-2767.144686	-2767.258613	239.780
**20**	-2767.80712548	-2767.216892	-2767.176195	-2767.175251	-2767.292726	247.249

Additional Tables Containing XYZ Coordinates of Computed Species

EtOH dimer

00000aaa_2EtOH_M062X_631dp_PCMetoh.log

Standard orientation:

Center Number	Atomic Number	Atomic Type	Coordinates (Angstroms) X	Y	Z
1	8	0	1.017326	-0.366348	-0.938096
2	1	0	0.119497	-0.724994	-0.824604
3	6	0	1.490240	-0.058306	0.359096
4	1	0	1.372680	-0.918848	1.034500
5	1	0	0.918090	0.775411	0.797259
6	6	0	2.953698	0.326629	0.259688
7	1	0	3.071298	1.190311	-0.400131
8	1	0	3.356573	0.582710	1.242537
9	1	0	3.535698	-0.501805	-0.152313
10	8	0	-1.540714	-1.132314	-0.116151
11	1	0	-1.537604	-1.894000	0.476039
12	6	0	-1.979317	0.015033	0.613143
13	1	0	-3.006144	-0.136636	0.966533
14	1	0	-1.340020	0.174438	1.490840
15	6	0	-1.905405	1.206211	-0.320175
16	1	0	-2.566288	1.056798	-1.177601
17	1	0	-2.208489	2.117530	0.199782
18	1	0	-0.883479	1.330983	-0.689374

$Pd^{2+}(Ph_2POH)_3$ (6)

00000ebc_Pd2+_Ph2POH3_M062X_631dp_PCMetoh_3.log

Standard orientation:

Center Number	Atomic Number	Atomic Type	Coordinates (Angstroms) X	Y	Z
1	15	0	0.180229	-1.077400	-1.340846
2	1	0	2.061384	3.435033	1.516455
3	15	0	1.763400	1.347704	0.804912
4	8	0	1.464539	2.953756	0.921169
5	15	0	-2.540092	0.651972	-0.999010
6	8	0	-2.651754	0.448972	-2.617683
7	6	0	3.436621	1.234177	0.133344
8	6	0	3.915642	2.273150	-0.672346
9	6	0	4.231046	0.108825	0.381642
10	6	0	5.198679	2.194973	-1.206347
11	1	0	3.294937	3.142419	-0.867018
12	6	0	5.511983	0.041005	-0.154859
13	1	0	3.857475	-0.703809	0.999006
14	6	0	5.996322	1.083046	-0.944946
15	1	0	5.575295	3.004396	-1.822348
16	1	0	6.130054	-0.829089	0.039479
17	1	0	6.996873	1.025372	-1.360543
18	6	0	1.912085	-1.308561	-1.751122
19	6	0	2.599562	-2.466048	-1.374784
20	6	0	2.519916	-0.349604	-2.573835
21	6	0	3.897230	-2.667283	-1.839728
22	1	0	2.126061	-3.213149	-0.746712
23	6	0	3.810580	-0.564166	-3.035564
24	1	0	1.982547	0.552895	-2.855073
25	6	0	4.495863	-1.724410	-2.670250

26	1	0	4.434406	-3.565689	-1.556691
27	1	0	4.287151	0.175386	-3.669413
28	1	0	5.506987	-1.886404	-3.029164
29	6	0	1.862258	0.703820	2.494992
30	6	0	3.007715	0.883168	3.283054
31	6	0	0.733565	0.081478	3.041590
32	6	0	3.017054	0.436548	4.599903
33	1	0	3.890250	1.361845	2.869088
34	6	0	0.749844	-0.370121	4.357913
35	1	0	-0.154875	-0.060305	2.431358
36	6	0	1.892289	-0.191709	5.134719
37	1	0	3.904082	0.574792	5.208200
38	1	0	-0.124366	-0.860149	4.772525
39	1	0	1.908447	-0.544464	6.160446
40	6	0	-3.255796	2.253248	-0.598699
41	6	0	-2.616215	3.395224	-1.108158
42	6	0	-4.381547	2.372647	0.223730
43	6	0	-3.120270	4.654112	-0.804864
44	1	0	-1.739613	3.295184	-1.743937
45	6	0	-4.872132	3.639347	0.525405
46	1	0	-4.872149	1.487865	0.616958
47	6	0	-4.244463	4.773506	0.012822
48	1	0	-2.635927	5.539515	-1.200978
49	1	0	-5.747624	3.739853	1.157048
50	1	0	-4.632861	5.757630	0.252148
51	6	0	-3.499822	-0.656837	-0.228300
52	6	0	-4.429497	-1.410397	-0.949181
53	6	0	-3.241887	-0.941682	1.118400
54	6	0	-5.113433	-2.441516	-0.310559
55	1	0	-4.615347	-1.208843	-1.999187
56	6	0	-3.931095	-1.970261	1.748115
57	1	0	-2.503040	-0.361399	1.666253
58	6	0	-4.865784	-2.718899	1.031940
59	1	0	-5.835280	-3.030707	-0.865134
60	1	0	-3.729802	-2.197290	2.789226
61	1	0	-5.395724	-3.529071	1.521479
62	6	0	-0.415042	-2.439651	-0.332989
63	6	0	0.105071	-2.628843	0.954208
64	6	0	-1.449218	-3.249368	-0.811382
65	6	0	-0.413879	-3.633567	1.761717
66	1	0	0.915133	-2.003539	1.322401
67	6	0	-1.963623	-4.250742	0.007734
68	1	0	-1.846336	-3.095773	-1.809595
69	6	0	-1.450740	-4.439224	1.289287
70	1	0	-0.010914	-3.785067	2.757187
71	1	0	-2.770948	-4.877723	-0.354720
72	1	0	-1.860467	-5.217031	1.924959
73	8	0	-0.508565	-1.201963	-2.786035
74	1	0	-1.310165	-0.664782	-2.956625
75	46	0	-0.322600	0.830871	-0.296600
76	1	0	-3.506154	0.649461	-3.034561

$Pd^{2+}(Ph_2POH)_2(Ph_2PO^-)$ (9)

00000ebb_Pd2+_Ph2POH3_-1H+_M062X_631dp_PCMetoh_3.log

Standard orientation:

Center Number	Atomic Number	Atomic Type	Coordinates (Angstroms) X	Y	Z
1	15	0	0.003079	-0.652421	1.736825
2	1	0	-2.169741	2.721347	-2.551029
3	15	0	-1.747482	1.017528	-1.184531
4	8	0	-1.517945	2.497850	-1.868341
5	15	0	2.544047	0.846491	0.842314
6	8	0	2.779738	0.957238	2.415179
7	6	0	-3.443323	1.105989	-0.540073
8	6	0	-3.976973	2.351144	-0.192954
9	6	0	-4.192422	-0.057277	-0.329636
10	6	0	-5.261597	2.432527	0.339309
11	1	0	-3.393739	3.253921	-0.347083
12	6	0	-5.474147	0.031474	0.202227
13	1	0	-3.785610	-1.030544	-0.591890
14	6	0	-6.010489	1.274923	0.534722
15	1	0	-5.675856	3.401051	0.598853
16	1	0	-6.052387	-0.872399	0.363270
17	1	0	-7.010999	1.339357	0.949661
18	6	0	-1.747983	-0.570674	2.184100
19	6	0	-2.500409	-1.736093	2.360267
20	6	0	-2.295562	0.676733	2.509365
21	6	0	-3.793690	-1.648802	2.870886
22	1	0	-2.077845	-2.705873	2.116363
23	6	0	-3.585234	0.755849	3.019200
24	1	0	-1.712076	1.582907	2.356992
25	6	0	-4.331625	-0.408222	3.202914
26	1	0	-4.378265	-2.551916	3.010155
27	1	0	-4.013833	1.722915	3.259329
28	1	0	-5.341923	-0.344368	3.594102
29	6	0	-1.853840	-0.154424	-2.572610
30	6	0	-3.011596	-0.283322	-3.351948
31	6	0	-0.713989	-0.897908	-2.903109
32	6	0	-3.024904	-1.148291	-4.441194
33	1	0	-3.902988	0.284630	-3.102182
34	6	0	-0.731980	-1.767454	-3.990282
35	1	0	0.187322	-0.806922	-2.300707
36	6	0	-1.888026	-1.891993	-4.757480
37	1	0	-3.923527	-1.246208	-5.040611
38	1	0	0.152088	-2.347508	-4.232983
39	1	0	-1.905340	-2.571330	-5.603287
40	6	0	3.242349	2.336092	0.099079
41	6	0	2.629666	3.561650	0.399061
42	6	0	4.327894	2.287203	-0.780515
43	6	0	3.118012	4.734154	-0.166213
44	1	0	1.778240	3.594643	1.075026
45	6	0	4.806236	3.466095	-1.347178
46	1	0	4.800681	1.338518	-1.014918
47	6	0	4.204242	4.684631	-1.040378
48	1	0	2.649622	5.683490	0.069507
49	1	0	5.652125	3.432089	-2.025118
50	1	0	4.580945	5.599775	-1.484827
51	6	0	3.469010	-0.587825	0.253146
52	6	0	4.408989	-1.199588	1.085523
53	6	0	3.176543	-1.130547	-1.003386
54	6	0	5.061138	-2.352455	0.653509
55	1	0	4.612093	-0.782020	2.066731
56	6	0	3.833781	-2.278309	-1.430363
57	1	0	2.433135	-0.657360	-1.640949
58	6	0	4.773518	-2.889780	-0.599280
59	1	0	5.789215	-2.833283	1.298024

60	1	0	3.605314	-2.703287	-2.401972
61	1	0	5.277583	-3.792519	-0.928451
62	6	0	0.355023	-2.258223	0.988633
63	6	0	-0.383883	-2.725703	-0.103668
64	6	0	1.462576	-2.975840	1.448745
65	6	0	-0.011977	-3.906010	-0.737538
66	1	0	-1.245446	-2.168026	-0.463072
67	6	0	1.830973	-4.157033	0.809652
68	1	0	2.030890	-2.600984	2.294315
69	6	0	1.098490	-4.618111	-0.282539
70	1	0	-0.584673	-4.265497	-1.586147
71	1	0	2.695962	-4.710591	1.159451
72	1	0	1.392711	-5.535164	-0.782369
73	8	0	0.851712	-0.456802	3.002753
74	1	0	2.010549	0.390059	2.840656
75	46	0	0.374660	0.862900	0.117159

$Pd^{2+}(Ph_2POH)_4$ (7)

00000ebc_Pd2+_Ph2POH4_tetra_M062X_631dp_PCMetoh_3.log

Standard orientation:

Center Number	Atomic Number	Atomic Type	Coordinates (Angstroms) X	Y	Z
1	15	0	-2.004702	1.541110	-0.471969
2	15	0	1.231591	-1.058628	1.499896
3	8	0	-2.321362	1.716029	-2.063773
4	1	0	-0.661929	1.509849	-2.901776
5	15	0	1.106179	1.142541	-1.616593
6	8	0	0.296963	1.377897	-3.013798
7	15	0	-1.862050	-1.705335	0.511408
8	8	0	-2.326799	-1.905509	2.041951
9	6	0	2.410153	0.032822	-2.209658
10	6	0	2.067660	-0.983931	-3.109263
11	6	0	3.715953	0.131620	-1.720403
12	6	0	3.033624	-1.901252	-3.513805
13	1	0	1.054194	-1.050030	-3.494332
14	6	0	4.673745	-0.797746	-2.120312
15	1	0	3.985711	0.933230	-1.037778
16	6	0	4.333636	-1.811115	-3.014836
17	1	0	2.772067	-2.685789	-4.215972
18	1	0	5.687124	-0.723074	-1.740635
19	1	0	5.083424	-2.530460	-3.327300
20	6	0	-3.598433	1.350786	0.360726
21	6	0	-4.797021	1.465833	-0.346448
22	6	0	-3.611224	1.061496	1.730499
23	6	0	-6.006872	1.317070	0.325856
24	1	0	-4.801180	1.638783	-1.417510
25	6	0	-4.824582	0.917553	2.393813
26	1	0	-2.684421	0.923555	2.281132
27	6	0	-6.021874	1.049271	1.692407
28	1	0	-6.937179	1.396375	-0.226052
29	1	0	-4.832986	0.691549	3.454339
30	1	0	-6.967607	0.928381	2.209593
31	6	0	2.248741	-2.522813	1.208518
32	6	0	2.675146	-3.315104	2.283653
33	6	0	2.634027	-2.840036	-0.093604
34	6	0	3.477056	-4.423167	2.045354
35	1	0	2.385370	-3.080831	3.303890
36	6	0	3.453004	-3.944586	-0.323329
37	1	0	2.286660	-2.240083	-0.928736
38	6	0	3.867730	-4.736542	0.741574
39	1	0	3.800830	-5.041577	2.875347
40	1	0	3.746213	-4.189367	-1.339190

41	1	0	4.495095	-5.602953	0.560445
42	6	0	1.926526	2.713259	-1.264959
43	6	0	2.260944	3.565778	-2.324389
44	6	0	2.240248	3.058855	0.049845
45	6	0	2.913569	4.763523	-2.057465
46	1	0	2.000662	3.294012	-3.342891
47	6	0	2.907936	4.254189	0.307119
48	1	0	1.948154	2.411244	0.871568
49	6	0	3.240000	5.105021	-0.743535
50	1	0	3.169185	5.431842	-2.872773
51	1	0	3.145901	4.525054	1.330953
52	1	0	3.748758	6.041661	-0.540581
53	6	0	-1.305067	3.126811	0.065035
54	6	0	-0.999624	4.121133	-0.868446
55	6	0	-0.988935	3.313089	1.417627
56	6	0	-0.409685	5.309479	-0.443378
57	1	0	-1.198836	3.976551	-1.925876
58	6	0	-0.397217	4.499808	1.832387
59	1	0	-1.197666	2.538474	2.149477
60	6	0	-0.113257	5.500334	0.902674
61	1	0	-0.170437	6.077491	-1.170741
62	1	0	-0.152015	4.639692	2.879720
63	1	0	0.355839	6.423041	1.227378
64	6	0	-0.993738	-3.220601	0.021901
65	6	0	-0.710173	-4.207077	0.968312
66	6	0	-0.540419	-3.353205	-1.297794
67	6	0	0.023577	-5.330448	0.589376
68	1	0	-1.053565	-4.098780	1.992372
69	6	0	0.177966	-4.482538	-1.669414
70	1	0	-0.739948	-2.578274	-2.033619
71	6	0	0.462320	-5.469413	-0.724225
72	1	0	0.252836	-6.094195	1.324633
73	1	0	0.531015	-4.584518	-2.690311
74	1	0	1.038490	-6.342466	-1.012084
75	6	0	-3.427472	-1.684826	-0.386196
76	6	0	-4.618841	-1.997409	0.271634
77	6	0	-3.428134	-1.363443	-1.748360
78	6	0	-5.812583	-1.995528	-0.445652
79	1	0	-4.612500	-2.225374	1.331967
80	6	0	-4.625242	-1.369874	-2.455892
81	1	0	-2.507092	-1.085035	-2.255416
82	6	0	-5.816479	-1.686812	-1.804060
83	1	0	-6.742210	-2.227372	0.063092
84	1	0	-4.628307	-1.118074	-3.510789
85	1	0	-6.750926	-1.683358	-2.355016
86	6	0	2.348028	0.177068	2.214093
87	6	0	3.688246	0.243393	1.817830
88	6	0	1.819228	1.143106	3.080391
89	6	0	4.489538	1.287025	2.273959
90	1	0	4.102737	-0.515513	1.159522
91	6	0	2.631151	2.174716	3.542995
92	1	0	0.779392	1.090224	3.391004
93	6	0	3.962265	2.250807	3.132224
94	1	0	5.527733	1.342088	1.965102
95	1	0	2.223530	2.920243	4.217365
96	1	0	4.591221	3.060785	3.486360
97	8	0	0.259632	-1.432193	2.781996
98	1	0	-1.576074	-1.739885	2.646131
99	46	0	-0.357340	-0.022903	-0.043912
100	1	0	-2.920300	2.442408	-2.307619
101	1	0	0.717039	-1.569353	3.627150

$Pd^{2+}(Ph_2POH)_2(Ph_2PO^-)$ (10)

00000ebb_Pd2+_Ph2POH4_-2H+_tetra_M062X_631dp_PCMetoh_3.log

Standard orientation:

Center Number	Atomic Number	Atomic Type	Coordinates (Angstroms) X	Y	Z
1	15	0	1.909666	1.592593	0.625984
2	15	0	-1.117001	-1.131543	-1.609910
3	8	0	2.083365	1.825877	2.144593
4	1	0	0.720801	1.599545	2.726385
5	15	0	-1.117778	1.127994	1.605284
6	8	0	-0.272982	1.426305	2.953896
7	15	0	1.908161	-1.591742	-0.625481
8	8	0	2.080304	-1.830975	-2.144027
9	6	0	-2.375371	-0.039633	2.218862
10	6	0	-1.981111	-1.046703	3.107403
11	6	0	-3.686957	-0.016376	1.734698
12	6	0	-2.891679	-2.023719	3.503226
13	1	0	-0.964514	-1.058708	3.490141
14	6	0	-4.591551	-1.001659	2.124937
15	1	0	-4.001471	0.769587	1.053025
16	6	0	-4.195577	-2.005030	3.007800
17	1	0	-2.583774	-2.799397	4.197049
18	1	0	-5.607600	-0.980931	1.744078
19	1	0	-4.902761	-2.769662	3.312797
20	6	0	3.546092	1.592839	-0.177008
21	6	0	4.660821	1.985039	0.564781
22	6	0	3.684299	1.270411	-1.530535
23	6	0	5.909092	2.066593	-0.050218
24	1	0	4.541790	2.217758	1.618612
25	6	0	4.930726	1.357791	-2.141971
26	1	0	2.826421	0.922804	-2.102436
27	6	0	6.043364	1.758774	-1.402370
28	1	0	6.776666	2.368466	0.527765
29	1	0	5.036330	1.102967	-3.191501
30	1	0	7.016218	1.822323	-1.879049
31	6	0	-2.058994	-2.653434	-1.290307
32	6	0	-2.335757	-3.531985	-2.344162
33	6	0	-2.504783	-2.945058	-0.000782
34	6	0	-3.062732	-4.692867	-2.104245
35	1	0	-1.967944	-3.305975	-3.340753
36	6	0	-3.244618	-4.103403	0.231680
37	1	0	-2.263924	-2.280984	0.825189
38	6	0	-3.520359	-4.977035	-0.816373
39	1	0	-3.273513	-5.377353	-2.919648
40	1	0	-3.585647	-4.326429	1.238152
41	1	0	-4.086952	-5.884179	-0.631602
42	6	0	-2.060215	2.650972	1.287468
43	6	0	-2.332978	3.530603	2.341193
44	6	0	-2.511052	2.940511	-0.000756
45	6	0	-3.061926	4.690487	2.102587
46	1	0	-1.960411	3.306086	3.336244
47	6	0	-3.252906	4.097875	-0.232035
48	1	0	-2.272368	2.275905	-0.826950
49	6	0	-3.525278	4.972407	0.816157
50	1	0	-3.269842	5.376138	2.917796
51	1	0	-3.598345	4.319339	-1.237356
52	1	0	-4.093515	5.878791	0.632657
53	6	0	1.116462	3.081304	-0.096470
54	6	0	0.843394	4.165554	0.739173
55	6	0	0.713301	3.120404	-1.437002
56	6	0	0.185483	5.287038	0.235361
57	1	0	1.134224	4.114972	1.784766
58	6	0	0.059532	4.240914	-1.937794
59	1	0	0.899145	2.269127	-2.088472

60	6	0	-0.203200	5.326340	-1.100698
61	1	0	-0.033364	6.124191	0.890691
62	1	0	-0.255822	4.263193	-2.976382
63	1	0	-0.725228	6.195337	-1.488509
64	6	0	1.117615	-3.078334	0.104322
65	6	0	0.864646	-4.175930	-0.720268
66	6	0	0.699811	-3.104260	1.440572
67	6	0	0.209630	-5.296247	-0.210030
68	1	0	1.167913	-4.136053	-1.762828
69	6	0	0.051252	-4.224600	1.948527
70	1	0	0.868690	-2.242305	2.082663
71	6	0	-0.193162	-5.322445	1.122262
72	1	0	0.005771	-6.143669	-0.856925
73	1	0	-0.275184	-4.236879	2.983869
74	1	0	-0.711823	-6.191113	1.515286
75	6	0	3.545917	-1.591448	0.174334
76	6	0	4.661578	-1.977607	-0.568965
77	6	0	3.682917	-1.273185	1.528916
78	6	0	5.910193	-2.056888	0.045765
79	1	0	4.542943	-2.207639	-1.623444
80	6	0	4.929627	-1.358121	2.140000
81	1	0	2.823105	-0.931661	2.101564
82	6	0	6.043560	-1.752862	1.398867
83	1	0	6.778742	-2.354172	-0.533137
84	1	0	5.034485	-1.106447	3.190368
85	1	0	7.016694	-1.814676	1.875201
86	6	0	-2.373843	0.034173	-2.226790
87	6	0	-3.686294	0.012243	-1.743754
88	6	0	-1.978308	1.042932	-3.113548
89	6	0	-4.589655	0.998612	-2.133765
90	1	0	-4.002358	-0.774363	-1.063498
91	6	0	-2.887588	2.021471	-3.508568
92	1	0	-0.961545	1.054826	-3.496068
93	6	0	-4.191910	2.003275	-3.014389
94	1	0	-5.606103	0.978118	-1.754042
95	1	0	-2.578093	2.798015	-4.200652
96	1	0	-4.898260	2.768812	-3.319030
97	8	0	-0.269267	-1.428823	-2.954380
98	1	0	0.726564	-1.604882	-2.725621
99	46	0	0.373429	0.002265	-0.001342

$Pd^{2+}(Ph_2POH)(Ph_2PO^-)$

00000ecb_Pd2+_2Ph2POH2_-1H+_M062X_631dp_PCMetoh_3.log

Standard orientation:

Center Number	Atomic Number	Atomic Type	Coordinates (Angstroms) X	Y	Z
1	15	0	1.533143	-0.191425	0.653745
2	15	0	-1.483846	-0.380371	0.544313
3	8	0	-1.273654	-0.642016	2.093653
4	6	0	3.052400	-1.101671	0.276409
5	6	0	4.217459	-0.459093	-0.156938
6	6	0	3.044581	-2.492261	0.467517
7	6	0	5.366811	-1.207606	-0.396317
8	1	0	4.234473	0.617113	-0.293327
9	6	0	4.196939	-3.231199	0.225443
10	1	0	2.139808	-2.989330	0.809769
11	6	0	5.355983	-2.588301	-0.208843
12	1	0	6.272561	-0.709806	-0.724984
13	1	0	4.191127	-4.305351	0.374400
14	1	0	6.254514	-3.165823	-0.398593
15	6	0	-2.954408	-1.312047	0.082725
16	6	0	-3.181766	-2.555465	0.685730

17	6	0	-3.813961	-0.837118	-0.913862
18	6	0	-4.282090	-3.312598	0.298486
19	1	0	-2.510497	-2.914203	1.459981
20	6	0	-4.908574	-1.606135	-1.298045
21	1	0	-3.639792	0.131349	-1.373958
22	6	0	-5.141634	-2.840035	-0.693336
23	1	0	-4.469807	-4.270388	0.771349
24	1	0	-5.582402	-1.238135	-2.063882
25	1	0	-5.998211	-3.434521	-0.992777
26	6	0	-1.782668	1.380592	0.305463
27	6	0	-2.243163	2.144756	1.382137
28	6	0	-1.497009	1.984294	-0.923875
29	6	0	-2.431444	3.514339	1.218116
30	1	0	-2.437472	1.670643	2.339214
31	6	0	-1.694906	3.351352	-1.081136
32	1	0	-1.098290	1.390720	-1.743728
33	6	0	-2.159719	4.114290	-0.009881
34	1	0	-2.784300	4.113080	2.050844
35	1	0	-1.465539	3.824521	-2.029840
36	1	0	-2.299788	5.183449	-0.130893
37	6	0	1.695962	1.525266	0.127207
38	6	0	2.017711	1.848999	-1.197118
39	6	0	1.347636	2.528354	1.035511
40	6	0	2.013523	3.179358	-1.600665
41	1	0	2.268191	1.065938	-1.909274
42	6	0	1.335224	3.857623	0.620596
43	1	0	1.077172	2.260071	2.052231
44	6	0	1.670134	4.181498	-0.692247
45	1	0	2.270390	3.434556	-2.623119
46	1	0	1.056815	4.638054	1.320717
47	1	0	1.656357	5.217887	-1.013350
48	8	0	1.163765	-0.282565	2.140508
49	1	0	-0.248218	-0.519164	2.291307
50	46	0	0.128575	-1.183266	-0.768146

$Pd^{2+}(Ph_2POH)_2$

00000ecc_Pd2+_2Ph2POH2_M062X_631dp_PCMetoh_3.log

Standard orientation:

Center Number	Atomic Number	Atomic Type	Coordinates (Angstroms) X	Y	Z
1	15	0	1.513166	-0.253640	0.463311
2	15	0	-1.575807	-0.308402	0.634318
3	8	0	-1.506289	-0.675647	2.188106
4	6	0	3.012786	-1.210113	0.205631
5	6	0	4.200811	-0.591259	-0.204800
6	6	0	2.972546	-2.595004	0.440312
7	6	0	5.345948	-1.362002	-0.376459
8	1	0	4.235316	0.478672	-0.380164
9	6	0	4.124951	-3.351176	0.268784
10	1	0	2.050979	-3.076078	0.759839
11	6	0	5.307300	-2.735063	-0.142047
12	1	0	6.268903	-0.887569	-0.690079
13	1	0	4.100226	-4.419473	0.451178
14	1	0	6.203861	-3.329896	-0.279465
15	6	0	-3.067181	-1.106010	0.056890
16	6	0	-3.319885	-2.419887	0.481743
17	6	0	-3.921559	-0.465307	-0.849487
18	6	0	-4.446171	-3.081076	0.008293
19	1	0	-2.651341	-2.906189	1.185653
20	6	0	-5.042354	-1.142135	-1.317642
21	1	0	-3.724728	0.553051	-1.169781
22	6	0	-5.302210	-2.443719	-0.890523

23	1	0	-4.656730	-4.091392	0.340150
24	1	0	-5.716412	-0.650509	-2.009770
25	1	0	-6.179900	-2.964476	-1.257829
26	6	0	-1.640213	1.466838	0.451401
27	6	0	-1.935645	2.262082	1.563785
28	6	0	-1.377914	2.042093	-0.798840
29	6	0	-1.979847	3.645007	1.412450
30	1	0	-2.124889	1.805471	2.529853
31	6	0	-1.438567	3.422156	-0.939029
32	1	0	-1.111389	1.415726	-1.646116
33	6	0	-1.736348	4.220065	0.166987
34	1	0	-2.204149	4.271715	2.268232
35	1	0	-1.232484	3.876127	-1.901895
36	1	0	-1.768678	5.298832	0.057111
37	6	0	1.748142	1.441026	-0.069019
38	6	0	2.004698	1.691372	-1.424279
39	6	0	1.584662	2.494034	0.835700
40	6	0	2.123479	3.004161	-1.863565
41	1	0	2.112441	0.870746	-2.129570
42	6	0	1.702507	3.804586	0.381882
43	1	0	1.354742	2.297183	1.877567
44	6	0	1.972985	4.057735	-0.960904
45	1	0	2.328964	3.205477	-2.908895
46	1	0	1.572630	4.626076	1.077762
47	1	0	2.059129	5.081245	-1.309774
48	8	0	1.196726	-0.212779	2.059789
49	1	0	-0.606610	-0.584427	2.560554
50	46	0	-0.091587	-1.338613	-0.633331
51	1	0	1.961664	-0.046482	2.637494

$Pd^{2+}(Ph_2POH)$

00000edb_Pd2+_Ph2POH1_M062X_631dp_PCMetoh_3.log

Standard orientation:

Center Number	Atomic Number	Atomic Type	Coordinates (Angstroms) X	Y	Z
1	15	0	0.000833	-0.226090	0.637150
2	8	0	-0.113089	-0.600531	2.176936
3	6	0	-1.420545	0.750486	0.251523
4	6	0	-2.680111	0.244814	0.621969
5	6	0	-1.291845	1.964885	-0.439663
6	6	0	-3.815056	0.978166	0.308097
7	1	0	-2.758358	-0.692542	1.163730
8	6	0	-2.439908	2.686195	-0.738278
9	1	0	-0.314827	2.348674	-0.713921
10	6	0	-3.692034	2.193279	-0.368140
11	1	0	-4.792619	0.607651	0.593459
12	1	0	-2.358432	3.636622	-1.252498
13	1	0	-4.582478	2.765477	-0.604862
14	6	0	1.582302	0.465619	0.238857
15	6	0	2.468171	0.791465	1.277205
16	6	0	1.930039	0.664622	-1.109547
17	6	0	3.705591	1.332584	0.956243
18	1	0	2.187810	0.642108	2.314162
19	6	0	3.172064	1.208001	-1.408751
20	1	0	1.242876	0.402233	-1.909113
21	6	0	4.053752	1.538925	-0.379082
22	1	0	4.396571	1.597241	1.748033
23	1	0	3.452219	1.372905	-2.442442
24	1	0	5.022175	1.963790	-0.619649
25	1	0	0.488480	-1.286806	2.516950
26	46	0	-0.228820	-2.045131	-0.500635

$Pd^{2+}(Ph_2POH)_2(Ph)(PhPO^-)$

00000edb_Pd2+_Ph2POH3_-1H+_defenil1_M062X_631dp_PCMetoh_3.log

Standard orientation:

Center Number	Atomic Number	Atomic Type	Coordinates (Angstroms) X	Y	Z
1	15	0	0.578831	-0.708431	-2.272058
2	1	0	3.210282	1.243241	2.661176
3	15	0	2.304027	0.171370	0.951700
4	8	0	2.323743	1.114038	2.290164
5	15	0	-2.269454	0.393128	-0.350861
6	8	0	-2.588738	0.700797	-1.894910
7	6	0	3.452034	0.977331	-0.192191
8	6	0	3.555875	2.372942	-0.161165
9	6	0	4.120647	0.247230	-1.182412
10	6	0	4.347259	3.028924	-1.100052
11	1	0	3.011763	2.942227	0.588500
12	6	0	4.907809	0.910760	-2.118885
13	1	0	4.034950	-0.835636	-1.218000
14	6	0	5.023379	2.299516	-2.076006
15	1	0	4.430470	4.110170	-1.071018
16	1	0	5.432542	0.342926	-2.879606
17	1	0	5.639108	2.813357	-2.806692
18	6	0	0.125328	2.050858	-0.004674
19	6	0	0.271918	2.653267	-1.248685
20	6	0	0.065704	2.800188	1.167882
21	6	0	0.377538	4.046178	-1.310949
22	1	0	0.290392	2.069655	-2.163716
23	6	0	0.171792	4.188449	1.086947
24	1	0	-0.052657	2.319882	2.133912
25	6	0	0.330285	4.811976	-0.150084
26	1	0	0.493950	4.524503	-2.278333
27	1	0	0.127161	4.778979	1.996587
28	1	0	0.411490	5.892249	-0.207516
29	6	0	3.069023	-1.401603	1.436804
30	6	0	4.458138	-1.562839	1.521521
31	6	0	2.224632	-2.454045	1.814033
32	6	0	4.991256	-2.766769	1.970972
33	1	0	5.120957	-0.752492	1.232450
34	6	0	2.762362	-3.657064	2.262857
35	1	0	1.144454	-2.340821	1.747037
36	6	0	4.144961	-3.812134	2.339039
37	1	0	6.067019	-2.889704	2.033036
38	1	0	2.104060	-4.469742	2.550798
39	1	0	4.565110	-4.750221	2.686194
40	6	0	-3.037249	1.781174	0.518105
41	6	0	-3.100083	3.028499	-0.109213
42	6	0	-3.446303	1.636972	1.846884
43	6	0	-3.577713	4.129205	0.595648
44	1	0	-2.767689	3.134112	-1.137133
45	6	0	-3.918701	2.743964	2.546617
46	1	0	-3.402619	0.665706	2.332921
47	6	0	-3.982435	3.988704	1.922256
48	1	0	-3.627973	5.098063	0.109971
49	1	0	-4.240663	2.633293	3.576547
50	1	0	-4.349947	4.849988	2.470261
51	6	0	-3.221625	-1.088375	0.083607
52	6	0	-4.340782	-1.430554	-0.682651
53	6	0	-2.819019	-1.906927	1.144611
54	6	0	-5.055032	-2.586267	-0.383662
55	1	0	-4.635362	-0.800617	-1.516580
56	6	0	-3.538055	-3.063760	1.437137
57	1	0	-1.947564	-1.654757	1.746361
58	6	0	-4.651792	-3.403694	0.672385
59	1	0	-5.922029	-2.853674	-0.978502

60	1	0	-3.223581	-3.701214	2.256583
61	1	0	-5.206335	-4.308619	0.897758
62	6	0	-0.181947	-2.291789	-1.808974
63	6	0	0.588969	-3.177885	-1.037545
64	6	0	-1.491006	-2.642527	-2.178231
65	6	0	0.045137	-4.384386	-0.607250
66	1	0	1.615131	-2.919743	-0.780824
67	6	0	-2.025286	-3.852232	-1.758363
68	1	0	-2.077344	-1.956735	-2.784650
69	6	0	-1.261720	-4.714237	-0.965091
70	1	0	0.637177	-5.064991	-0.004339
71	1	0	-3.039537	-4.123252	-2.031503
72	1	0	-1.689987	-5.653774	-0.630680
73	8	0	-0.456390	0.027351	-3.114539
74	1	0	-1.812316	0.446006	-2.491435
75	46	0	-0.001786	0.079105	0.196427

TS $Pd^{2}(Ph_2POH)_2(Ph)(PhPO^-)$

00000edc_Pd2+_Ph2POH3_-1H+_defenil1_M062X_631dp_PCMetoh_SCANvissza_3_step7_FRQ.log

Standard orientation:

Center Number	Atomic Number	Atomic Type	Coordinates (Angstroms) X	Y	Z
1	15	0	-0.201947	-0.090947	-2.275941
2	1	0	-3.088226	-0.505584	3.132884
3	15	0	-2.310471	0.116396	1.150940
4	8	0	-2.340633	-0.727330	2.555916
5	15	0	2.275489	-0.760561	-0.150613
6	8	0	2.711847	-1.482554	-1.489569
7	6	0	-3.840725	-0.337553	0.296641
8	6	0	-4.521302	-1.502517	0.657548
9	6	0	-4.253974	0.399157	-0.821753
10	6	0	-5.621678	-1.920806	-0.087865
11	1	0	-4.186581	-2.085890	1.509453
12	6	0	-5.347852	-0.029102	-1.566337
13	1	0	-3.725394	1.304050	-1.112233
14	6	0	-6.031489	-1.189074	-1.199483
15	1	0	-6.152825	-2.822556	0.197264
16	1	0	-5.667935	0.542432	-2.430730
17	1	0	-6.883855	-1.520992	-1.782568
18	6	0	-0.585545	-2.090164	-0.694646
19	6	0	-1.837650	-2.263716	-1.291761
20	6	0	0.208628	-3.209765	-0.445475
21	6	0	-2.256887	-3.535942	-1.677267
22	1	0	-2.486178	-1.424552	-1.503001
23	6	0	-0.219347	-4.479787	-0.839884
24	1	0	1.155893	-3.141527	0.072676
25	6	0	-1.450518	-4.649191	-1.460670
26	1	0	-3.228409	-3.639954	-2.149801
27	1	0	0.420879	-5.332848	-0.639714
28	1	0	-1.782546	-5.635772	-1.765118
29	6	0	-2.534071	1.870932	1.583732
30	6	0	-3.796703	2.472234	1.670273
31	6	0	-1.389970	2.634750	1.850045
32	6	0	-3.905109	3.818610	2.005878
33	1	0	-4.692576	1.893500	1.469412
34	6	0	-1.500881	3.980618	2.183949
35	1	0	-0.405192	2.178854	1.803914
36	6	0	-2.760095	4.573115	2.256295
37	1	0	-4.884775	4.279871	2.067577
38	1	0	-0.607963	4.563987	2.381470
39	1	0	-2.849364	5.624491	2.508619
40	6	0	2.895117	-1.800303	1.194610

41	6	0	3.597056	-2.976966	0.922558
42	6	0	2.662168	-1.400362	2.515460
43	6	0	4.063804	-3.754249	1.980816
44	1	0	3.771082	-3.275752	-0.106362
45	6	0	3.131126	-2.182167	3.565001
46	1	0	2.121762	-0.479411	2.720660
47	6	0	3.830136	-3.359519	3.296220
48	1	0	4.610789	-4.668096	1.775682
49	1	0	2.952071	-1.874935	4.589547
50	1	0	4.194122	-3.969377	4.116215
51	6	0	3.221866	0.780932	-0.040401
52	6	0	4.406955	0.873235	-0.778865
53	6	0	2.808319	1.845550	0.764306
54	6	0	5.172550	2.032050	-0.710602
55	1	0	4.713275	0.044931	-1.410123
56	6	0	3.574666	3.006734	0.818522
57	1	0	1.896148	1.786811	1.352430
58	6	0	4.753397	3.100171	0.081472
59	1	0	6.091292	2.104826	-1.282885
60	1	0	3.247200	3.838951	1.432523
61	1	0	5.346535	4.007653	0.123840
62	6	0	0.595913	1.562943	-2.155110
63	6	0	-0.105313	2.607252	-1.530475
64	6	0	1.859420	1.814942	-2.704559
65	6	0	0.465524	3.870709	-1.419146
66	1	0	-1.102024	2.430377	-1.128189
67	6	0	2.420420	3.084088	-2.612618
68	1	0	2.395813	1.005875	-3.194050
69	6	0	1.729857	4.106072	-1.960133
70	1	0	-0.074493	4.668806	-0.919876
71	1	0	3.401813	3.275550	-3.034265
72	1	0	2.177613	5.091354	-1.877977
73	8	0	0.855150	-0.968649	-2.976602
74	1	0	1.973936	-1.310472	-2.243622
75	46	0	-0.033385	-0.369937	0.170977

H_2O

0000aaa_H2O_M062X_631dp_PCMetoh.log

Standard orientation:

Center Number	Atomic Number	Atomic Type	Coordinates (Angstroms) X	Y	Z
1	8	0	0.000000	0.000000	0.118971
2	1	0	0.000000	0.757514	-0.475886
3	1	0	0.000000	-0.757514	-0.475886

Ph_2PHO
0001aaa_Ph2PHO_M062X_631dp_PCMetoh.log

Standard orientation:

Center Number	Atomic Number	Atomic Type	Coordinates (Angstroms) X	Y	Z
1	15	0	-0.003020	1.386670	-0.315254
2	8	0	-0.129450	2.579715	0.588689
3	1	0	0.118340	1.714361	-1.681683
4	6	0	1.443118	0.332403	-0.069113
5	6	0	2.532770	0.425359	-0.938494
6	6	0	1.491364	-0.544995	1.020715
7	6	0	3.666442	-0.354119	-0.719704
8	1	0	2.496663	1.102642	-1.787616
9	6	0	2.625587	-1.318967	1.238564
10	1	0	0.638838	-0.628025	1.689927
11	6	0	3.711712	-1.222919	0.367936
12	1	0	4.510553	-0.284452	-1.397401
13	1	0	2.662740	-2.000609	2.081604
14	1	0	4.594439	-1.830822	0.537405
15	6	0	-1.431674	0.280464	-0.211726
16	6	0	-1.445343	-0.961672	-0.854545
17	6	0	-2.541400	0.704513	0.520479
18	6	0	-2.571377	-1.773135	-0.765377
19	1	0	-0.577044	-1.297008	-1.416279
20	6	0	-3.665607	-0.113712	0.612028
21	1	0	-2.510426	1.669912	1.016433
22	6	0	-3.679828	-1.348710	-0.031496
23	1	0	-2.584522	-2.737333	-1.262366
24	1	0	-4.527747	0.212179	1.184310
25	1	0	-4.555523	-1.985679	0.039368

Ph_2POH conf. 1
0001aba_Ph2POH_A_M062X_631dp_PCMetoh.log

Standard orientation:

Center Number	Atomic Number	Atomic Type	Coordinates (Angstroms) X	Y	Z
1	15	0	-0.023718	1.321821	-0.914844
2	8	0	-0.171271	2.503720	0.251042
3	1	0	-0.015109	3.372805	-0.138382
4	6	0	1.403874	0.368319	-0.258907
5	6	0	2.190398	-0.366798	-1.148545
6	6	0	1.712511	0.355884	1.105852
7	6	0	3.262852	-1.125677	-0.678992
8	1	0	1.968999	-0.343710	-2.213047
9	6	0	2.791802	-0.387293	1.571686
10	1	0	1.105496	0.938320	1.793357
11	6	0	3.564597	-1.133453	0.679745
12	1	0	3.867076	-1.698543	-1.375015
13	1	0	3.030930	-0.390966	2.630493
14	1	0	4.404233	-1.715475	1.045950
15	6	0	-1.382814	0.228067	-0.332683
16	6	0	-1.413497	-1.110447	-0.741467
17	6	0	-2.428239	0.728207	0.446830
18	6	0	-2.467986	-1.937179	-0.368101
19	1	0	-0.602361	-1.512956	-1.344580
20	6	0	-3.482873	-0.103209	0.822989
21	1	0	-2.405246	1.764620	0.766882
22	6	0	-3.506222	-1.434542	0.416540

23	1	0	-2.478992	-2.974936	-0.685807
24	1	0	-4.287137	0.292069	1.435704
25	1	0	-4.328378	-2.079599	0.709083

Ph_2POH conf. 2

0001aca_Ph2POH_B_M062X_631dp_PCMetoh.log

Standard orientation:

Center Number	Atomic Number	Atomic Type	Coordinates (Angstroms) X	Y	Z
1	15	0	0.026732	1.453617	-0.589344
2	8	0	-0.064738	2.435817	0.751184
3	1	0	-0.077783	1.939350	1.584007
4	6	0	1.412528	0.319674	-0.164862
5	6	0	2.601751	0.444518	-0.889543
6	6	0	1.344290	-0.616462	0.876044
7	6	0	3.705925	-0.349661	-0.583130
8	1	0	2.662219	1.166153	-1.700029
9	6	0	2.447137	-1.404385	1.188091
10	1	0	0.419533	-0.738282	1.435204
11	6	0	3.628448	-1.271753	0.456820
12	1	0	4.622574	-0.248120	-1.154814
13	1	0	2.386331	-2.126303	1.996068
14	1	0	4.486317	-1.891419	0.697450
15	6	0	-1.397017	0.327358	-0.291295
16	6	0	-1.464389	-0.906661	-0.951220
17	6	0	-2.465601	0.724743	0.516768
18	6	0	-2.573742	-1.730609	-0.794471
19	1	0	-0.638745	-1.230881	-1.580544
20	6	0	-3.575565	-0.104392	0.676902
21	1	0	-2.429262	1.684566	1.023468
22	6	0	-3.632088	-1.331426	0.022520
23	1	0	-2.611915	-2.687262	-1.305599
24	1	0	-4.396035	0.211479	1.313382
25	1	0	-4.496366	-1.975745	0.146352

Ph-P=O

0001agb_Ph1PO_M062X_631dp_PCMetoh.log

Standard orientation:

Center Number	Atomic Number	Atomic Type	Coordinates (Angstroms) X	Y	Z
1	15	0	1.974085	-0.535070	0.000205
2	6	0	0.191284	-0.185346	-0.000536
3	6	0	-0.308656	1.124474	-0.000501
4	6	0	-0.689946	-1.274094	-0.000292
5	6	0	-1.680012	1.340071	0.000100
6	1	0	0.392999	1.953894	-0.000965
7	6	0	-2.064841	-1.056250	0.000097
8	1	0	-0.299052	-2.289594	-0.000179
9	6	0	-2.554220	0.249053	0.000345
10	1	0	-2.075742	2.350298	0.000613
11	1	0	-2.752299	-1.895264	-0.000032
12	1	0	-3.625940	0.420626	0.000709
13	8	0	2.673388	0.787329	0.000186

Ph-P=OH+ conf 1 (21)

0001aha_Ph1POH+_M062X_631dp_PCMetoh.log

Standard orientation:

Center Number	Atomic Number	Atomic Type	Coordinates (Angstroms) X	Y	Z
1	15	0	1.852875	-0.632843	-0.000419
2	6	0	0.170706	-0.192539	0.000609
3	6	0	-0.305242	1.140509	0.000404
4	6	0	-0.733004	-1.282534	0.000574
5	6	0	-1.666455	1.367053	-0.000026
6	1	0	0.400858	1.963403	0.000462
7	6	0	-2.096243	-1.036762	-0.000096
8	1	0	-0.362817	-2.304501	0.001042
9	6	0	-2.554687	0.281473	-0.000473
10	1	0	-2.050946	2.380111	-0.000066
11	1	0	-2.800210	-1.860057	-0.000365
12	1	0	-3.622811	0.472349	-0.001110
13	8	0	2.531077	0.798303	0.000088
14	1	0	3.503732	0.791715	-0.000348

Ph-P=OH+ conf 2

0001ahb_Ph1PHO+_M062X_631dp_PCMetoh.log

Standard orientation:

Center Number	Atomic Number	Atomic Type	Coordinates (Angstroms) X	Y	Z
1	15	0	1.839332	-0.376261	0.000005
2	6	0	0.140060	-0.136213	-0.000227
3	6	0	-0.379965	1.177477	-0.000158
4	6	0	-0.695644	-1.274583	-0.000120
5	6	0	-1.751818	1.338697	0.000037
6	1	0	0.288017	2.032059	-0.000447
7	6	0	-2.066052	-1.083734	0.000030
8	1	0	-0.282014	-2.277702	-0.000054
9	6	0	-2.585474	0.213575	0.000168
10	1	0	-2.180660	2.333281	0.000153
11	1	0	-2.730341	-1.939066	-0.000007
12	1	0	-3.661249	0.352438	0.000332
13	8	0	2.846422	0.681146	0.000141
14	1	0	2.238244	-1.717573	0.000436

(Ph)(OEt)PHO

0001aka_Ph1PHOOEt_M062X_631dp_PCMetoh.log

Standard orientation:

Center Number	Atomic Number	Atomic Type	Coordinates (Angstroms) X	Y	Z
1	15	0	-0.735171	0.863029	0.417295
2	8	0	-1.660017	-0.313493	-0.179249
3	6	0	0.901861	0.171045	0.166163
4	6	0	1.917061	1.021915	-0.275207
5	6	0	1.170735	-1.177223	0.422565
6	6	0	3.203938	0.523624	-0.463016
7	1	0	1.688658	2.064252	-0.475228
8	6	0	2.458015	-1.669399	0.235279
9	1	0	0.375957	-1.837391	0.756792
10	6	0	3.472189	-0.819013	-0.206406
11	1	0	3.994483	1.180670	-0.809455
12	1	0	2.671365	-2.715075	0.429683
13	1	0	4.475184	-1.206822	-0.352128
14	8	0	-0.921903	2.226751	-0.151048
15	6	0	-3.087452	-0.198647	0.002377
16	1	0	-3.445487	0.667551	-0.561782
17	1	0	-3.299379	-0.033657	1.065878
18	6	0	-3.721235	-1.480657	-0.487672
19	1	0	-3.349420	-2.332918	0.085090
20	1	0	-4.805699	-1.426343	-0.370428
21	1	0	-3.491756	-1.638424	-1.543558
22	1	0	-1.021657	0.796790	1.793590

(Ph)(OEt)POH (22)

0001akb_Ph1POHOEt_M062X_631dp_PCMetoh.log

Standard orientation:

Center Number	Atomic Number	Atomic Type	Coordinates (Angstroms) X	Y	Z
1	15	0	0.765141	0.934031	-0.581004
2	8	0	1.657780	-0.216298	0.199901
3	6	0	-0.859186	0.186318	-0.202381
4	6	0	-1.961169	1.002200	0.070096
5	6	0	-1.034600	-1.198635	-0.288175
6	6	0	-3.219020	0.438747	0.272165
7	1	0	-1.827164	2.077253	0.141319
8	6	0	-2.292482	-1.760369	-0.086006
9	1	0	-0.179995	-1.835395	-0.496019
10	6	0	-3.386135	-0.942439	0.193915
11	1	0	-4.068099	1.077691	0.493161
12	1	0	-2.419382	-2.836703	-0.144173
13	1	0	-4.366421	-1.380969	0.350754
14	8	0	0.667798	2.176296	0.512090
15	1	0	1.190400	2.928039	0.204494
16	6	0	3.066902	-0.232566	-0.060224
17	1	0	3.546204	0.580263	0.497833
18	1	0	3.249024	-0.061912	-1.128701
19	6	0	3.610834	-1.576590	0.375206
20	1	0	3.135666	-2.380406	-0.192161
21	1	0	4.689452	-1.619724	0.206941
22	1	0	3.417704	-1.738579	1.438119

$Ph_2(OEt)P{=}O$

0001akd_Ph2POOEt_M062X_631dp_PCMetoh.log

Standard orientation:

Center Number	Atomic Number	Atomic Type	Coordinates (Angstroms) X	Y	Z
1	15	0	0.039879	0.489151	0.843505
2	8	0	-0.165451	1.876985	0.045265
3	6	0	1.622626	-0.036162	0.163650
4	6	0	2.485942	-0.751225	0.997163
5	6	0	1.982075	0.223597	-1.163081
6	6	0	3.706382	-1.207395	0.505402
7	1	0	2.200572	-0.935539	2.028454
8	6	0	3.202977	-0.232647	-1.649271
9	1	0	1.317751	0.793506	-1.805442
10	6	0	4.062454	-0.949247	-0.816414
11	1	0	4.378883	-1.759597	1.153187
12	1	0	3.486554	-0.027164	-2.676054
13	1	0	5.013942	-1.303738	-1.199359
14	8	0	0.020820	0.552581	2.333827
15	6	0	-1.358372	2.648316	0.301493
16	1	0	-1.281784	3.082996	1.302114
17	1	0	-2.230919	1.985429	0.275462
18	6	0	-1.454436	3.714803	-0.765816
19	1	0	-1.534859	3.257652	-1.754928
20	1	0	-2.338021	4.333413	-0.594037
21	1	0	-0.570297	4.355587	-0.745590
22	6	0	-1.256203	-0.587787	0.190150
23	6	0	-1.817929	-1.552087	1.029949
24	6	0	-1.677198	-0.486831	-1.140755
25	6	0	-2.793598	-2.416865	0.540115
26	1	0	-1.492343	-1.610914	2.063999
27	6	0	-2.654249	-1.350007	-1.625699
28	1	0	-1.251243	0.274175	-1.789505
29	6	0	-3.209064	-2.315972	-0.785903
30	1	0	-3.231276	-3.165001	1.192517
31	1	0	-2.985848	-1.268844	-2.655412
32	1	0	-3.970679	-2.988692	-1.166604

CO_3^{2-}

0002aaa_CO32-_M062X_631dp_PCMetoh_f3FRQ.log

Standard orientation:

Center Number	Atomic Number	Atomic Type	Coordinates (Angstroms) X	Y	Z
1	6	0	-0.097278	-0.015759	-0.255734
2	8	0	-0.661866	-1.171636	-0.159179
3	8	0	-0.750234	1.050097	0.062637
4	8	0	1.121560	0.073038	-0.670852
5	8	0	2.294327	-2.162966	-0.892048
6	1	0	1.772697	-1.280451	-0.870767
7	8	0	-3.079494	-1.111978	0.596904
8	1	0	-2.084768	-1.110527	0.343655
9	8	0	0.549490	3.205742	-0.234693
10	1	0	0.027754	2.326107	-0.171718
11	6	0	-3.721675	-0.366417	-0.399975
12	6	0	-4.993615	0.255204	0.158948
13	1	0	-3.990954	-0.993549	-1.271853
14	1	0	-3.055843	0.425054	-0.772321
15	1	0	-5.528295	0.830976	-0.602757

16	1	0	-5.664302	-0.524240	0.534205
17	1	0	-4.751380	0.920595	0.993011
18	6	0	2.262846	-2.623694	0.430387
19	6	0	2.533704	-4.120507	0.461030
20	1	0	1.283948	-2.412717	0.884914
21	1	0	3.020288	-2.116860	1.058920
22	1	0	2.545061	-4.506130	1.484964
23	1	0	1.762537	-4.653993	-0.102399
24	1	0	3.501078	-4.341382	-0.000765
25	6	0	1.665687	3.038324	0.596015
26	6	0	2.800083	3.933442	0.119121
27	1	0	1.989172	1.987817	0.588155
28	1	0	1.435284	3.296725	1.647864
29	1	0	3.677732	3.850726	0.767590
30	1	0	3.092361	3.659957	-0.899184
31	1	0	2.478854	4.979964	0.107594

HCO_3^-

0002faa_HCO3_M062X_631dp_PCMetoh.log

Standard orientation:

Center Number	Atomic Number	Atomic Type	Coordinates (Angstroms) X	Y	Z
1	6	0	-0.427986	-1.471173	-0.647068
2	8	0	-0.271499	-0.975393	0.621590
3	8	0	-1.556755	-1.894672	-0.986399
4	8	0	0.609438	-1.429615	-1.353507
5	1	0	-1.141482	-0.988873	1.064241
6	8	0	-3.055302	-1.247662	1.053405
7	1	0	-2.645534	-1.630656	0.233467
8	6	0	-3.735925	-0.070438	0.647108
9	6	0	-2.788491	1.085765	0.355279
10	1	0	-4.417367	0.203835	1.459277
11	1	0	-4.355399	-0.276893	-0.236358
12	1	0	-3.350927	1.970413	0.041213
13	1	0	-2.217123	1.345936	1.252410
14	1	0	-2.076499	0.828785	-0.436385
15	8	0	0.457496	1.416141	-1.314020
16	1	0	0.712751	0.486169	-1.180963
17	8	0	2.930537	-1.581202	0.031298
18	1	0	2.083421	-1.666454	-0.456759
19	6	0	3.003619	-0.221300	0.394454
20	6	0	4.305413	0.023843	1.134448
21	1	0	2.149573	0.059290	1.029630
22	1	0	2.957486	0.429631	-0.495554
23	1	0	4.393401	1.071936	1.433144
24	1	0	4.353184	-0.598707	2.032130
25	1	0	5.157831	-0.229171	0.497960
26	6	0	0.570065	2.119915	-0.092440
27	6	0	-0.143557	3.450282	-0.241696
28	1	0	1.626724	2.297893	0.163820
29	1	0	0.129973	1.538526	0.728032
30	1	0	-0.059680	4.040549	0.674591
31	1	0	0.292890	4.024113	-1.064126
32	1	0	-1.203371	3.291554	-0.459214

O=B-O$^-$

000aaa_BOH-1H+_M062X_631dp_PCMetoh.log

Standard orientation:

Center Number	Atomic Number	Atomic Type	Coordinates (Angstroms) X	Y	Z
1	5	0	0.000000	0.000000	0.000000
2	8	0	0.000000	0.000000	1.258225
3	8	0	0.000000	0.000000	-1.258225

Pd(OAc)$_2$

000ba_PdAc2_M062X_631dp_PCMetoh_1_3_f.log

Standard orientation:

Center Number	Atomic Number	Atomic Type	Coordinates (Angstroms) X	Y	Z
1	46	0	-0.000404	0.007261	-0.010559
2	8	0	1.766600	-1.083189	-0.010976
3	6	0	2.443947	0.000063	-0.003940
4	8	0	1.788042	1.090108	-0.009562
5	8	0	-1.769721	-1.086260	-0.012602
6	6	0	-2.442914	-0.003834	-0.005760
7	8	0	-1.784498	1.086877	-0.011522
8	6	0	3.935131	-0.019666	0.039830
9	1	0	4.312286	-0.841197	-0.569469
10	1	0	4.333515	0.934019	-0.303273
11	1	0	4.247019	-0.187028	1.074324
12	6	0	-3.934099	-0.015959	0.041396
13	1	0	-4.329495	0.900183	-0.395628
14	1	0	-4.315398	-0.893299	-0.480378
15	1	0	-4.245095	-0.070593	1.088251

O=B-OH

000baa_BOH_M062X_631dp_PCMetoh.log

Standard orientation:

Center Number	Atomic Number	Atomic Type	Coordinates (Angstroms) X	Y	Z
1	5	0	-0.117420	-0.001753	-0.000006
2	8	0	-1.333629	0.018574	-0.000072
3	8	0	1.193937	-0.106593	0.000069
4	1	0	1.704635	0.712917	0.000051

$B(OH)_2(O)^-$

000caa_BOH3_-H+_M062X_631dp_PCMetoh.log

Standard orientation:

Center Number	Atomic Number	Atomic Type	Coordinates (Angstroms) X	Y	Z
1	5	0	0.097658	-0.127989	-0.001439
2	8	0	-1.292743	-0.461514	0.000958
3	8	0	0.246413	1.301771	-0.000500
4	8	0	1.062087	-0.988547	0.000007
5	1	0	-1.806952	0.351813	-0.002469
6	1	0	1.192607	1.474453	0.005934

$B(OH)_3$

000caa_BOH3_M062X_631dp_PCMetoh.log

Standard orientation:

Center Number	Atomic Number	Atomic Type	Coordinates (Angstroms) X	Y	Z
1	5	0	0.000029	-0.000049	-0.000009
2	8	0	1.342512	-0.264669	-0.000235
3	8	0	-0.900570	-1.030171	0.000119
4	8	0	-0.441991	1.294837	0.000120
5	1	0	0.288566	1.922717	0.000027
6	1	0	1.521194	-1.211283	-0.000384
7	1	0	-1.809505	-0.711166	0.000371

O_2

000dab_O2_M02_2X_631dp_PCMw_TRIPLET.log

Standard orientation:

Center Number	Atomic Number	Atomic Type	Coordinates (Angstroms) X	Y	Z
1	8	0	0.000000	0.000000	0.598675
2	8	0	0.000000	0.000000	-0.598675

$Pd^{2+}(Ph_2PO^-)(Ph_2POH)(AcO^-)$ (8)

000maa_Pd1_PPh2O_anion_PPh2OH_AcO_M062X_631dp_PCMetoh_3.log

Standard orientation:

Center Number	Atomic Number	Atomic Type	Coordinates (Angstroms) X	Y	Z
1	46	0	0.024031	-0.008509	0.681147
2	6	0	0.071373	-0.073056	3.256972
3	8	0	1.158274	-0.056864	2.602662
4	8	0	-1.043406	-0.048039	2.655455
5	6	0	0.111471	-0.159548	4.758794
6	1	0	-0.803746	0.245715	5.188723
7	1	0	0.194186	-1.212979	5.040891
8	1	0	0.984950	0.366678	5.143486
9	15	0	1.574803	0.011787	-0.926311
10	8	0	1.165908	0.015374	-2.419712

11	6	0	2.616312	-1.451957	-0.641182
12	6	0	3.144341	-1.739569	0.623520
13	6	0	2.883166	-2.295277	-1.723008
14	6	0	3.943636	-2.865568	0.795579
15	1	0	2.918459	-1.099722	1.472207
16	6	0	3.682752	-3.421779	-1.541224
17	1	0	2.465390	-2.059983	-2.696437
18	6	0	4.212976	-3.705942	-0.284876
19	1	0	4.353754	-3.090006	1.774694
20	1	0	3.891813	-4.075646	-2.381484
21	1	0	4.835137	-4.583944	-0.145281
22	6	0	2.619880	1.471073	-0.629551
23	6	0	3.036186	2.215062	-1.737357
24	6	0	3.014141	1.847965	0.660272
25	6	0	3.852688	3.328953	-1.556053
26	1	0	2.715308	1.916205	-2.729981
27	6	0	3.832724	2.960843	0.831549
28	1	0	2.672662	1.286992	1.526366
29	6	0	4.252133	3.700162	-0.274027
30	1	0	4.176014	3.906100	-2.416051
31	1	0	4.138752	3.254885	1.830143
32	1	0	4.887392	4.568770	-0.134647
33	15	0	-1.586976	0.023498	-0.864744
34	8	0	-1.231903	0.048937	-2.400051
35	1	0	-0.138275	0.035624	-2.526377
36	6	0	-2.642427	-1.433352	-0.616753
37	6	0	-3.037737	-1.816611	0.670805
38	6	0	-3.067056	-2.161441	-1.731295
39	6	0	-3.865695	-2.923253	0.834116
40	1	0	-2.688749	-1.264809	1.540323
41	6	0	-3.892629	-3.269956	-1.557081
42	1	0	-2.748110	-1.857397	-2.722747
43	6	0	-4.292778	-3.649009	-0.277665
44	1	0	-4.172810	-3.223248	1.830506
45	1	0	-4.222788	-3.836478	-2.421455
46	1	0	-4.935265	-4.513288	-0.145076
47	6	0	-2.631772	1.478314	-0.566107
48	6	0	-3.027681	1.815076	0.734264
49	6	0	-3.045905	2.252944	-1.652747
50	6	0	-3.845454	2.922561	0.938158
51	1	0	-2.686618	1.226559	1.582655
52	6	0	-3.861363	3.361916	-1.437902
53	1	0	-2.726704	1.984539	-2.654342
54	6	0	-4.261931	3.694981	-0.145907
55	1	0	-4.152851	3.186635	1.944576
56	1	0	-4.183385	3.964721	-2.280530
57	1	0	-4.896432	4.559742	0.018294

AcOH+2EtOH

000mba_AcOH_2EtOH_M062X_631dp_PCMetoh.log

Standard orientation:

Center Number	Atomic Number	Atomic Type	Coordinates (Angstroms) X	Y	Z
1	6	0	-1.303751	-1.404487	0.023750
2	8	0	-0.658004	-1.265324	1.054124
3	8	0	-1.239036	-0.575443	-0.999722
4	1	0	-0.640571	0.219204	-0.784948
5	6	0	-2.258143	-2.543635	-0.201055
6	1	0	-3.262606	-2.146539	-0.363045
7	1	0	-1.968467	-3.086191	-1.103424
8	1	0	-2.251718	-3.210282	0.658355
9	8	0	0.145586	1.499535	-0.363489
10	1	0	0.899079	1.121403	0.150464
11	6	0	-0.724078	2.162811	0.561603
12	1	0	-0.174430	2.968873	1.059245
13	1	0	-1.055615	1.451332	1.329611
14	6	0	-1.907349	2.713750	-0.206897
15	1	0	-2.589687	3.234459	0.468516
16	1	0	-1.568848	3.416182	-0.972185
17	1	0	-2.455258	1.903559	-0.695606
18	6	0	3.483113	-0.051410	-0.705574
19	1	0	3.942321	-0.629278	-1.510582
20	1	0	4.251418	0.190600	0.032655
21	1	0	3.099331	0.882716	-1.125614
22	6	0	2.363396	-0.840052	-0.059024
23	1	0	1.581481	-1.069272	-0.797189
24	1	0	2.736749	-1.791593	0.337329
25	8	0	1.818135	-0.054872	1.002323
26	1	0	1.004239	-0.508198	1.293715

AcO^{-}+2EtOH

000mbb_AcO-_2EtOH_M062X_631dp_PCMetoh_f.log

Standard orientation:

Center Number	Atomic Number	Atomic Type	Coordinates (Angstroms) X	Y	Z
1	6	0	-2.091079	-0.656783	0.008121
2	8	0	-1.211805	-1.159344	-0.740635
3	8	0	-1.891860	-0.093816	1.116100
4	6	0	-3.538558	-0.702617	-0.484896
5	1	0	-4.210008	-0.956624	0.337801
6	1	0	-3.820902	0.292830	-0.841603
7	1	0	-3.658031	-1.414664	-1.301776
8	8	0	0.299491	1.420906	1.050086
9	1	0	-0.389603	0.724156	1.174587
10	6	0	0.038862	1.969022	-0.221890
11	1	0	-0.954481	2.447399	-0.248540
12	1	0	0.028037	1.179822	-0.989277
13	6	0	1.107103	2.997958	-0.543943
14	1	0	0.924691	3.463186	-1.516405
15	1	0	1.120750	3.781979	0.218599
16	1	0	2.095394	2.528635	-0.564733
17	8	0	1.138571	-1.677774	0.388806
18	1	0	0.246823	-1.418697	0.040327
19	6	0	2.092514	-0.937346	-0.334454
20	1	0	1.915056	-1.013374	-1.420653
21	1	0	2.036577	0.128992	-0.072084

22	6	0	3.477063	-1.474310	-0.014224
23	1	0	3.552545	-2.528787	-0.294573
24	1	0	4.250943	-0.915798	-0.547987
25	1	0	3.671608	-1.394374	1.059186

$Pd^{2+}(Ph_2PO^-)(Ph_2POH)(AcO^-)$

000naa_Pd1_PPh2O_PPh2OH_AcO-_M062X_631dp_PCMetoh_3.log

Standard orientation:

Center Number	Atomic Number	Atomic Type	Coordinates (Angstroms) X	Y	Z
1	46	0	-0.083710	-1.119371	0.184474
2	6	0	-0.162084	-3.044997	1.812208
3	8	0	0.955199	-2.556680	1.453860
4	8	0	-1.239016	-2.555198	1.349810
5	6	0	-0.205940	-4.218479	2.745859
6	1	0	-1.141108	-4.222394	3.304711
7	1	0	-0.152370	-5.133834	2.149760
8	1	0	0.649507	-4.195445	3.420363
9	15	0	1.545936	0.137507	-0.675958
10	8	0	1.198479	0.610524	-2.204394
11	6	0	3.087067	-0.789637	-0.740623
12	6	0	4.315586	-0.159079	-0.508451
13	6	0	3.037232	-2.151210	-1.068566
14	6	0	5.492008	-0.894051	-0.615839
15	1	0	4.352823	0.893751	-0.246531
16	6	0	4.218700	-2.875405	-1.176394
17	1	0	2.080096	-2.640316	-1.227528
18	6	0	5.442510	-2.246525	-0.949163
19	1	0	6.446146	-0.410791	-0.437594
20	1	0	4.184517	-3.929348	-1.428706
21	1	0	6.362818	-2.815504	-1.028207
22	6	0	1.834281	1.647305	0.259753
23	6	0	1.712692	2.902234	-0.339809
24	6	0	2.111142	1.532246	1.628265
25	6	0	1.886295	4.047457	0.433191
26	1	0	1.468313	2.987682	-1.393630
27	6	0	2.281922	2.681796	2.390461
28	1	0	2.192208	0.552318	2.093678
29	6	0	2.170161	3.937440	1.792079
30	1	0	1.787366	5.024738	-0.026898
31	1	0	2.499787	2.598938	3.449523
32	1	0	2.299244	4.832880	2.390788
33	15	0	-1.595463	0.140686	-0.872080
34	8	0	-1.501191	0.127379	-2.484778
35	1	0	-0.582063	0.258436	-2.787772
36	6	0	-3.224630	-0.555665	-0.564465
37	6	0	-4.231462	0.191399	0.052665
38	6	0	-3.455487	-1.882724	-0.950006
39	6	0	-5.477057	-0.391694	0.273383
40	1	0	-4.050453	1.218933	0.351472
41	6	0	-4.703856	-2.452074	-0.733209
42	1	0	-2.661851	-2.464251	-1.411449
43	6	0	-5.711940	-1.706723	-0.119972
44	1	0	-6.263250	0.184568	0.748442
45	1	0	-4.889519	-3.477018	-1.034600
46	1	0	-6.684078	-2.155946	0.053500
47	6	0	-1.577456	1.860805	-0.342977
48	6	0	-1.391174	2.154926	1.014306
49	6	0	-1.720295	2.885529	-1.281471
50	6	0	-1.364528	3.480188	1.430206
51	1	0	-1.258897	1.352905	1.737584
52	6	0	-1.683425	4.211477	-0.855279

53	1	0	-1.855524	2.647011	-2.331659
54	6	0	-1.509135	4.506008	0.494622
55	1	0	-1.215027	3.714053	2.478613
56	1	0	-1.790036	5.012241	-1.578890
57	1	0	-1.475695	5.540254	0.821025
58	1	0	1.956048	0.912861	-2.731857

PhB(OH)$_2$

001aab_PhBOH2_M062X_631dp_PCMetoh.log

Standard orientation:

Center Number	Atomic Number	Atomic Type	Coordinates (Angstroms) X	Y	Z
1	6	0	-0.176192	0.017178	0.000001
2	6	0	0.563818	1.207099	-0.000085
3	6	0	1.956235	1.194379	-0.000085
4	6	0	2.637160	-0.021447	0.000004
5	6	0	1.922366	-1.218411	0.000088
6	6	0	0.530683	-1.194066	0.000082
7	1	0	0.058684	2.170889	-0.000169
8	1	0	2.509363	2.128169	-0.000158
9	1	0	3.722678	-0.035862	0.000006
10	1	0	2.450971	-2.166533	0.000156
11	1	0	-0.025675	-2.127110	0.000143
12	5	0	-1.748339	0.001449	0.000005
13	8	0	-2.528858	1.123556	0.000159
14	1	0	-2.038656	1.951562	0.000349
15	8	0	-2.372077	-1.213217	-0.000187
16	1	0	-3.332616	-1.119471	-0.000158

PhB(OH)(O)$^-$

001baa_PhBOH2-H+_M062X_631dp_PCMetoh.log

Standard orientation:

Center Number	Atomic Number	Atomic Type	Coordinates (Angstroms) X	Y	Z
1	6	0	-0.221298	0.030799	-0.000049
2	6	0	0.541880	1.206512	-0.000019
3	6	0	1.935334	1.184578	0.000008
4	6	0	2.609016	-0.037139	0.000006
5	6	0	1.877470	-1.224143	-0.000024
6	6	0	0.483016	-1.181937	-0.000051
7	1	0	0.009087	2.154908	-0.000019
8	1	0	2.499632	2.113521	0.000029
9	1	0	3.695003	-0.063687	0.000026
10	1	0	2.396259	-2.179282	-0.000025
11	1	0	-0.080621	-2.111906	-0.000076
12	5	0	-1.834269	0.133114	-0.000092
13	8	0	-2.493425	1.244844	0.000073
14	8	0	-2.422200	-1.172571	0.000068
15	1	0	-3.375528	-1.029338	0.000167

$(Ph_2PO^-)(Ph_2POH)$

001maa_PPh2O_anion_PPh2OH_M062X_631dp_PCMetoh_3.log

Standard orientation:

Center Number	Atomic Number	Atomic Type	Coordinates (Angstroms) X	Y	Z
1	15	0	-1.889191	-0.159777	0.485633
2	8	0	-1.170480	-1.467537	-0.069440
3	6	0	-2.311512	0.778180	-1.064023
4	6	0	-2.899810	2.045919	-1.007093
5	6	0	-1.998648	0.235794	-2.312621
6	6	0	-3.191579	2.747393	-2.174523
7	1	0	-3.128773	2.493785	-0.041639
8	6	0	-2.281701	0.938141	-3.483258
9	1	0	-1.531017	-0.744620	-2.343216
10	6	0	-2.882050	2.194356	-3.417267
11	1	0	-3.651902	3.729340	-2.116635
12	1	0	-2.034745	0.506058	-4.448859
13	1	0	-3.101176	2.743687	-4.327593
14	6	0	-3.611854	-0.764961	0.827546
15	6	0	-3.977476	-2.070731	0.490507
16	6	0	-4.553559	0.059841	1.451787
17	6	0	-5.263270	-2.538776	0.757054
18	1	0	-3.234709	-2.707917	0.018860
19	6	0	-5.841743	-0.402012	1.712442
20	1	0	-4.277702	1.071523	1.743707
21	6	0	-6.199767	-1.704601	1.365284
22	1	0	-5.536790	-3.555547	0.489805
23	1	0	-6.564302	0.249732	2.194573
24	1	0	-7.200753	-2.068664	1.574892
25	15	0	1.875409	-0.245169	-0.400719
26	8	0	1.175479	-1.401696	0.478360
27	1	0	0.086513	-1.466934	0.229800
28	6	0	3.565796	-0.956680	-0.644690
29	6	0	4.461859	-0.354166	-1.533300
30	6	0	3.960129	-2.110330	0.038340
31	6	0	5.738665	-0.880407	-1.718641
32	1	0	4.159025	0.528740	-2.092796
33	6	0	5.232992	-2.643985	-0.153150
34	1	0	3.252437	-2.580486	0.714872
35	6	0	6.126412	-2.028032	-1.028602
36	1	0	6.426763	-0.402554	-2.409321
37	1	0	5.530114	-3.541353	0.381624
38	1	0	7.117495	-2.444551	-1.178627
39	6	0	2.335515	0.998979	0.895027
40	6	0	2.992383	2.182673	0.542103
41	6	0	1.992578	0.788305	2.232474
42	6	0	3.319617	3.128188	1.510329
43	1	0	3.250310	2.370125	-0.498666
44	6	0	2.312602	1.737277	3.202892
45	1	0	1.475618	-0.129523	2.497053
46	6	0	2.978928	2.907370	2.845107
47	1	0	3.833541	4.040984	1.224465
48	1	0	2.042580	1.562906	4.240360
49	1	0	3.227168	3.646908	3.599918

Pd^{2+}(Ph$_2$POH)$_2$---PhB(OH)(O) (11)

205aab_Hirao_Pd2+_2Ph2POH_+PhBOH-_M062X_631dp_PCMetoh_uj.log

Standard orientation:

Center Number	Atomic Number	Atomic Type	Coordinates (Angstroms) X	Y	Z
1	6	0	3.444976	-1.808257	2.067410
2	6	0	2.766247	-3.025759	1.923545
3	6	0	1.383024	-3.078241	2.035427
4	6	0	0.629488	-1.917126	2.292409
5	6	0	1.330962	-0.698552	2.433620
6	6	0	2.731627	-0.649166	2.332500
7	1	0	4.526104	-1.777226	1.980624
8	1	0	3.328343	-3.932206	1.721407
9	1	0	0.869506	-4.029539	1.929967
10	1	0	0.795007	0.202609	2.736328
11	1	0	3.248134	0.292863	2.483940
12	46	0	-0.167074	-0.252049	0.386667
13	15	0	1.463152	0.625467	-0.954410
14	8	0	0.848448	1.289293	-2.331949
15	1	0	1.483910	1.751290	-2.902133
16	15	0	-1.912891	0.261895	-0.915248
17	8	0	-1.658112	0.494052	-2.484154
18	1	0	-0.776108	0.884339	-2.671935
19	6	0	-3.109120	-1.084041	-0.946989
20	6	0	-2.922138	-2.114749	-1.874246
21	6	0	-4.177545	-1.120999	-0.047476
22	6	0	-3.815037	-3.181299	-1.902764
23	1	0	-2.093499	-2.075518	-2.574510
24	6	0	-5.065548	-2.191702	-0.084523
25	1	0	-4.316001	-0.320906	0.672192
26	6	0	-4.883799	-3.219734	-1.008285
27	1	0	-5.579143	-4.052178	-1.033482
28	6	0	2.746291	-0.527618	-1.475034
29	6	0	3.904056	-0.067626	-2.120463
30	6	0	2.555102	-1.898652	-1.272215
31	6	0	4.862404	-0.977104	-2.550174
32	1	0	4.063268	0.995742	-2.278683
33	6	0	3.524165	-2.803533	-1.699084
34	1	0	1.660185	-2.266364	-0.775864
35	6	0	4.673371	-2.343275	-2.335643
36	1	0	5.426619	-3.049663	-2.668059
37	6	0	2.250355	2.035854	-0.138548
38	6	0	1.464910	3.184417	0.037132
39	6	0	3.551405	1.991422	0.371994
40	6	0	1.987835	4.284303	0.707210
41	1	0	0.450952	3.219798	-0.354413
42	6	0	4.065584	3.096214	1.046757
43	1	0	4.160824	1.101469	0.249981
44	6	0	3.287045	4.239059	1.212973
45	1	0	3.691989	5.096923	1.739090
46	6	0	-2.694965	1.758741	-0.277339
47	6	0	-2.795894	1.972800	1.103576
48	6	0	-3.188770	2.701656	-1.184649
49	6	0	-3.408447	3.131728	1.569832
50	1	0	-2.403573	1.230773	1.794249
51	6	0	-3.791576	3.861184	-0.704317
52	1	0	-3.098179	2.529077	-2.252020
53	6	0	-3.902495	4.074210	0.668247
54	1	0	-4.372985	4.979404	1.037429
55	1	0	3.378276	-3.865245	-1.532094
56	1	0	5.757256	-0.621546	-3.048700
57	1	0	5.075789	3.062004	1.439688
58	1	0	1.381794	5.174146	0.837183
59	1	0	-3.678053	-3.979006	-2.624669

60	1	0	-5.900668	-2.221699	0.606782
61	1	0	-4.173803	4.597355	-1.403055
62	1	0	-3.495362	3.301735	2.637530
63	5	0	-0.966951	-1.928241	2.333347
64	8	0	-1.574862	-2.977053	2.981043
65	1	0	-2.533499	-2.917599	2.880137
66	8	0	-1.619467	-0.947631	1.694961

Pd^{2+}(Ph$_2$POH)(Ph$_2$PO)$^-$---PhB(OH)(O)$^-$ (11)

227aab_Hirao_Pd2+_2Ph2POH_-H+_+PhBOH-_M062X_631dp_PCMetoh_uj.log

Standard orientation:

Center Number	Atomic Number	Atomic Type	Coordinates (Angstroms) X	Y	Z
1	6	0	3.770125	0.182554	2.632889
2	6	0	3.418618	-1.075475	3.137885
3	6	0	2.080176	-1.440424	3.238485
4	6	0	1.050268	-0.563730	2.848697
5	6	0	1.429871	0.698134	2.352035
6	6	0	2.775885	1.069553	2.238622
7	1	0	4.815533	0.461908	2.549279
8	1	0	4.195384	-1.768548	3.446471
9	1	0	1.818089	-2.416650	3.636592
10	1	0	0.663327	1.433094	2.101195
11	1	0	3.036383	2.048914	1.845857
12	46	0	-0.189804	-0.226715	0.313075
13	15	0	1.195762	0.137417	-1.437960
14	8	0	0.590870	0.320740	-2.894855
15	15	0	-1.989102	0.071380	-0.968175
16	8	0	-1.791654	0.128966	-2.496855
17	1	0	-0.476866	0.245652	-2.834133
18	6	0	-3.143158	-1.282428	-0.614819
19	6	0	-3.152332	-2.367262	-1.497651
20	6	0	-3.995366	-1.271255	0.493255
21	6	0	-4.011608	-3.438871	-1.270477
22	1	0	-2.498688	-2.358839	-2.364601
23	6	0	-4.855024	-2.343894	0.711855
24	1	0	-3.984693	-0.432223	1.180281
25	6	0	-4.861629	-3.427337	-0.165898
26	1	0	-5.533732	-4.261146	0.009009
27	6	0	2.344620	-1.256648	-1.547446
28	6	0	2.516843	-1.942217	-2.751134
29	6	0	3.026369	-1.669544	-0.396201
30	6	0	3.377190	-3.038129	-2.801620
31	1	0	1.979363	-1.615494	-3.635415
32	6	0	3.884640	-2.761880	-0.453779
33	1	0	2.894050	-1.132615	0.540715
34	6	0	4.058756	-3.446900	-1.657350
35	1	0	4.726440	-4.301027	-1.701779
36	6	0	2.200051	1.606014	-1.067102
37	6	0	1.532162	2.759434	-0.634281
38	6	0	3.593954	1.604529	-1.162542
39	6	0	2.254202	3.901331	-0.303705
40	1	0	0.447202	2.758910	-0.545780
41	6	0	4.314266	2.748112	-0.822588
42	1	0	4.118708	0.712780	-1.491156
43	6	0	3.647014	3.892965	-0.392367
44	1	0	4.211293	4.779828	-0.123357
45	6	0	-2.747874	1.628187	-0.413843
46	6	0	-2.805074	1.995838	0.936860
47	6	0	-3.265583	2.476428	-1.397812
48	6	0	-3.399639	3.203109	1.293938
49	1	0	-2.381506	1.336301	1.691142

50	6	0	-3.852987	3.684784	-1.030282
51	1	0	-3.198649	2.186353	-2.441453
52	6	0	-3.922075	4.046415	0.313502
53	1	0	-4.378814	4.988886	0.597654
54	1	0	4.414554	-3.078414	0.439049
55	1	0	3.514687	-3.571982	-3.736032
56	1	0	5.396829	2.742970	-0.892461
57	1	0	1.734119	4.792069	0.032260
58	1	0	-4.021833	-4.277971	-1.958135
59	1	0	-5.521797	-2.333378	1.567684
60	1	0	-4.253960	4.343615	-1.793397
61	1	0	-3.449589	3.489578	2.339377
62	5	0	-0.492364	-0.985073	2.957562
63	8	0	-0.804066	-1.767382	4.058027
64	1	0	-1.734372	-2.020654	4.012624
65	8	0	-1.371968	-0.627067	2.024745

TS $Pd^{2+}(Ph_2POH)(Ph_2PO)^{-}$---$PhB(OH)(O)^{-}$ (12)

228cab_Hirao_Pd2+_2Ph2POH_-H+_+PhBOH-_M062X_631dp_PCMetoh_SCAN_CB_FRQ_step.log

Standard orientation:

Center Number	Atomic Number	Atomic Type	Coordinates (Angstroms) X	Y	Z
1	6	0	4.222698	-1.731288	2.265454
2	6	0	3.567238	-2.652766	1.447770
3	6	0	2.258303	-2.407376	1.037565
4	6	0	1.576543	-1.241480	1.429120
5	6	0	2.255749	-0.334710	2.260737
6	6	0	3.566277	-0.571280	2.673743
7	1	0	5.243911	-1.918798	2.582548
8	1	0	4.078648	-3.556310	1.129363
9	1	0	1.760472	-3.134546	0.398784
10	1	0	1.753651	0.572192	2.592856
11	1	0	4.075211	0.144914	3.311922
12	46	0	-0.177234	-0.644146	0.292717
13	15	0	1.127029	0.495868	-1.135722
14	8	0	0.527892	0.655804	-2.599081
15	15	0	-2.060306	0.040672	-0.874339
16	8	0	-1.867975	0.473254	-2.360463
17	1	0	-0.569905	0.589927	-2.577776
18	6	0	-3.339873	-1.258800	-0.869115
19	6	0	-3.503678	-2.018014	-2.031948
20	6	0	-4.114232	-1.545369	0.260047
21	6	0	-4.434556	-3.053767	-2.065519
22	1	0	-2.909723	-1.780863	-2.909292
23	6	0	-5.043272	-2.581484	0.222390
24	1	0	-3.996929	-0.959273	1.166069
25	6	0	-5.203270	-3.337272	-0.938429
26	1	0	-5.929234	-4.143458	-0.964796
27	6	0	2.758379	-0.234741	-1.456283
28	6	0	2.852878	-1.218335	-2.446341
29	6	0	3.887233	0.112751	-0.710945
30	6	0	4.070575	-1.847650	-2.688253
31	1	0	1.976039	-1.478949	-3.031580
32	6	0	5.103707	-0.518086	-0.957943
33	1	0	3.818426	0.868645	0.065514
34	6	0	5.196902	-1.497345	-1.944631
35	1	0	6.146440	-1.986971	-2.135181
36	6	0	1.451654	2.170291	-0.498158
37	6	0	0.737822	2.662808	0.598698
38	6	0	2.359449	3.004650	-1.163657
39	6	0	0.931531	3.974070	1.030101
40	1	0	0.022348	2.025057	1.113118

41	6	0	2.552445	4.311719	-0.730014
42	1	0	2.916973	2.628160	-2.017210
43	6	0	1.838670	4.796178	0.367160
44	1	0	1.989935	5.817173	0.702083
45	6	0	-2.774720	1.441531	0.049394
46	6	0	-2.774695	1.450382	1.450270
47	6	0	-3.243398	2.553349	-0.653573
48	6	0	-3.263871	2.557491	2.138418
49	1	0	-2.376951	0.597020	1.997109
50	6	0	-3.725515	3.662148	0.039512
51	1	0	-3.213791	2.545733	-1.739010
52	6	0	-3.738046	3.663673	1.432990
53	1	0	-4.109849	4.529783	1.970780
54	1	0	5.977739	-0.245725	-0.375206
55	1	0	4.141541	-2.606666	-3.460458
56	1	0	3.258059	4.953840	-1.246508
57	1	0	0.369759	4.350355	1.878822
58	1	0	-4.562575	-3.635551	-2.972622
59	1	0	-5.645764	-2.796816	1.098873
60	1	0	-4.086894	4.526541	-0.508151
61	1	0	-3.265398	2.562658	3.223629
62	5	0	-0.285971	-1.941872	2.485387
63	8	0	0.281924	-2.428552	3.590322
64	1	0	-0.363413	-2.822777	4.195077
65	8	0	-1.328153	-1.728128	1.804082

$Pd^{2+}(Ph_2POH)(Ph_2PO^-)(Ph)$---$B(OH)(O)^-$ (13)

229aab_Hirao_Pd2+_2Ph2POH_-H+_+PhBOH-_M062X_631dp_PCMetoh_vege_uj1.log

Standard orientation:

Center Number	Atomic Number	Atomic Type	Coordinates (Angstroms) X	Y	Z
1	6	0	3.560720	2.641841	-2.717862
2	6	0	3.388041	2.925252	-1.359396
3	6	0	2.484142	2.186320	-0.595014
4	6	0	1.709694	1.161236	-1.163630
5	6	0	1.902445	0.889904	-2.530286
6	6	0	2.822488	1.611060	-3.300498
7	1	0	4.263889	3.216389	-3.311425
8	1	0	3.959183	3.725168	-0.897706
9	1	0	2.381717	2.420298	0.462426
10	1	0	1.310169	0.117591	-3.018863
11	1	0	2.948027	1.378728	-4.353617
12	46	0	0.015755	0.461007	-0.247486
13	15	0	1.210352	-0.847418	1.112567
14	8	0	0.477775	-1.183293	2.437197
15	15	0	-2.059617	-0.238029	0.739540
16	8	0	-1.951672	-1.015363	2.137976
17	1	0	-0.921844	-1.133914	2.349904
18	6	0	-3.213429	1.130032	1.097881
19	6	0	-3.383822	1.554900	2.418093
20	6	0	-3.881613	1.794462	0.063231
21	6	0	-4.220931	2.632845	2.701437
22	1	0	-2.870708	1.029891	3.217597
23	6	0	-4.715176	2.871068	0.350709
24	1	0	-3.750903	1.469801	-0.965313
25	6	0	-4.885303	3.291908	1.669711
26	1	0	-5.537103	4.130688	1.891512
27	6	0	2.812649	-0.128215	1.600910
28	6	0	2.822993	0.728261	2.705786
29	6	0	3.989684	-0.341822	0.879689
30	6	0	3.998668	1.376313	3.077899
31	1	0	1.908749	0.881965	3.271853

32	6	0	5.165550	0.300941	1.258039
33	1	0	3.990758	-1.004907	0.019129
34	6	0	5.169669	1.165804	2.351902
35	1	0	6.086023	1.669892	2.641470
36	6	0	1.608787	-2.421921	0.280151
37	6	0	2.016264	-2.482537	-1.056754
38	6	0	1.484692	-3.601023	1.021134
39	6	0	2.318178	-3.710920	-1.639190
40	1	0	2.098471	-1.568884	-1.638218
41	6	0	1.779139	-4.828862	0.432572
42	1	0	1.150740	-3.544483	2.052449
43	6	0	2.198664	-4.884101	-0.895141
44	1	0	2.427233	-5.841277	-1.352521
45	6	0	-3.021117	-1.327263	-0.358474
46	6	0	-2.948236	-1.147834	-1.744156
47	6	0	-3.825947	-2.338710	0.173479
48	6	0	-3.693601	-1.965891	-2.591152
49	1	0	-2.310170	-0.368375	-2.156673
50	6	0	-4.560438	-3.160111	-0.677366
51	1	0	-3.863094	-2.481327	1.249354
52	6	0	-4.497476	-2.971538	-2.057931
53	1	0	-5.071388	-3.613316	-2.718490
54	1	0	6.078455	0.128708	0.697195
55	1	0	4.000927	2.042327	3.934679
56	1	0	1.679404	-5.742338	1.009847
57	1	0	2.639433	-3.753176	-2.674796
58	1	0	-4.355809	2.954871	3.729007
59	1	0	-5.235073	3.380499	-0.454164
60	1	0	-5.182154	-3.948067	-0.264720
61	1	0	-3.639571	-1.824097	-3.665628
62	5	0	-0.346843	2.694598	-2.151958
63	8	0	0.357421	3.665491	-2.663894
64	1	0	1.291790	3.468909	-2.862212
65	8	0	-1.138646	1.870972	-1.686796

$Pd^{2+}(Ph_2POH)(Ph_2PO^-)(Ph)$ (14)

231aaa_Hirao_Pd2+_2Ph2POH_-H+_+Ph_M062X_631dp_PCMetoh.log

Standard orientation:

Center Number	Atomic Number	Atomic Type	Coordinates (Angstroms) X	Y	Z
1	6	0	4.180643	-2.116936	2.808609
2	6	0	3.919338	-2.655427	1.546905
3	6	0	2.816821	-2.219236	0.812030
4	6	0	1.943939	-1.249889	1.325945
5	6	0	2.215500	-0.725626	2.597678
6	6	0	3.324797	-1.152491	3.333408
7	1	0	5.046102	-2.447238	3.374076
8	1	0	4.580432	-3.410925	1.131714
9	1	0	2.653417	-2.626458	-0.183222
10	1	0	1.556975	0.024875	3.031965
11	1	0	3.518707	-0.728004	4.314215
12	46	0	0.167219	-0.819827	0.485271
13	15	0	1.210792	0.420800	-1.041592
14	8	0	0.524866	0.288762	-2.417325
15	15	0	-2.018751	-0.371582	-0.523520
16	8	0	-1.928621	-0.125050	-2.119389
17	1	0	-0.934231	0.042460	-2.346360
18	6	0	-3.183591	-1.764483	-0.387806
19	6	0	-3.580215	-2.481113	-1.518175
20	6	0	-3.645496	-2.149581	0.877064
21	6	0	-4.439233	-3.571799	-1.384447
22	1	0	-3.219807	-2.175647	-2.495069

23	6	0	-4.500460	-3.238889	1.006488
24	1	0	-3.343477	-1.590875	1.760696
25	6	0	-4.898063	-3.951390	-0.125772
26	1	0	-5.565147	-4.801145	-0.024317
27	6	0	2.988333	0.140666	-1.314940
28	6	0	3.348597	-0.739066	-2.338461
29	6	0	3.977584	0.712041	-0.509394
30	6	0	4.689364	-1.056403	-2.546837
31	1	0	2.576007	-1.169083	-2.968528
32	6	0	5.315873	0.398756	-0.724428
33	1	0	3.709890	1.398364	0.288021
34	6	0	5.672619	-0.491684	-1.737805
35	1	0	6.716918	-0.738445	-1.899519
36	6	0	1.013728	2.134509	-0.446605
37	6	0	1.352730	2.515266	0.857867
38	6	0	0.368354	3.042129	-1.289514
39	6	0	1.062439	3.800474	1.305491
40	1	0	1.815951	1.797873	1.530767
41	6	0	0.070669	4.325633	-0.834272
42	1	0	0.084095	2.726788	-2.288752
43	6	0	0.416145	4.704589	0.460612
44	1	0	0.176681	5.701336	0.816712
45	6	0	-2.986463	1.026763	0.139859
46	6	0	-2.325342	2.030737	0.855083
47	6	0	-4.360608	1.145572	-0.105038
48	6	0	-3.027571	3.142772	1.317529
49	1	0	-1.257977	1.950460	1.047671
50	6	0	-5.059549	2.255607	0.356893
51	1	0	-4.881733	0.365163	-0.653498
52	6	0	-4.392766	3.254420	1.068689
53	1	0	-4.941233	4.118669	1.429330
54	1	0	6.081407	0.847955	-0.100238
55	1	0	4.964813	-1.741941	-3.341616
56	1	0	-0.439121	5.025615	-1.488189
57	1	0	1.326295	4.093022	2.316539
58	1	0	-4.750441	-4.123330	-2.265728
59	1	0	-4.857852	-3.532266	1.988089
60	1	0	-6.124028	2.343136	0.165509
61	1	0	-2.503986	3.918222	1.867733

$Pd^{2+}(Ph_2POH)_2(Ph_2PO^-)(Ph)$ (15)

233aaa_Hirao_Pd2+_3Ph2POH_-H+_+Ph_M062X_631dp_PCMetoh.log

Standard orientation:

Center Number	Atomic Number	Atomic Type	Coordinates (Angstroms) X	Y	Z
1	6	0	2.687096	-4.264113	0.537095
2	6	0	2.196957	-3.947150	-0.729666
3	6	0	1.511780	-2.748541	-0.939266
4	6	0	1.286626	-1.845871	0.108021
5	6	0	1.769275	-2.191768	1.376637
6	6	0	2.470567	-3.379282	1.591222
7	1	0	3.228212	-5.191189	0.698786
8	1	0	2.357730	-4.628655	-1.560970
9	1	0	1.173450	-2.510089	-1.946354
10	1	0	1.582852	-1.536249	2.226513
11	1	0	2.837574	-3.617525	2.585969
12	46	0	0.232330	-0.116806	-0.205516
13	15	0	2.140484	0.820646	-1.140034
14	8	0	1.852212	1.712925	-2.385619
15	15	0	-0.928735	1.928511	-0.721702
16	8	0	-0.442584	2.564396	-2.119227
17	1	0	0.546757	2.231261	-2.312523

18	6	0	-2.731682	1.834264	-0.968299
19	6	0	-3.203164	1.313064	-2.179253
20	6	0	-3.641666	2.192444	0.028753
21	6	0	-4.569368	1.169284	-2.394939
22	1	0	-2.496215	1.025801	-2.952867
23	6	0	-5.010436	2.044902	-0.189202
24	1	0	-3.283085	2.590977	0.973104
25	6	0	-5.474653	1.537137	-1.399751
26	1	0	-6.540947	1.421567	-1.567076
27	6	0	3.511641	-0.284355	-1.624918
28	6	0	3.670856	-0.562274	-2.984371
29	6	0	4.337550	-0.917755	-0.690719
30	6	0	4.642935	-1.468366	-3.406764
31	1	0	3.033073	-0.060078	-3.705209
32	6	0	5.308293	-1.819331	-1.113804
33	1	0	4.218411	-0.721215	0.370084
34	6	0	5.459919	-2.099781	-2.472414
35	1	0	6.215651	-2.806630	-2.799918
36	6	0	2.810068	1.900603	0.179353
37	6	0	3.001211	1.435044	1.486120
38	6	0	2.996994	3.255667	-0.102304
39	6	0	3.406491	2.310283	2.489459
40	1	0	2.807154	0.391530	1.723733
41	6	0	3.389366	4.133411	0.906920
42	1	0	2.815143	3.613887	-1.111373
43	6	0	3.599014	3.661427	2.200562
44	1	0	3.900115	4.346364	2.986883
45	6	0	-0.712299	3.235261	0.528839
46	6	0	-0.144176	2.928454	1.767498
47	6	0	-1.054306	4.559889	0.230955
48	6	0	0.076902	3.935915	2.705526
49	1	0	0.139861	1.902871	1.992213
50	6	0	-0.831210	5.564247	1.166594
51	1	0	-1.487186	4.798980	-0.736796
52	6	0	-0.265592	5.251428	2.404197
53	1	0	-0.087509	6.037756	3.130784
54	1	0	5.944042	-2.308644	-0.382653
55	1	0	4.762263	-1.678202	-4.465014
56	1	0	3.526013	5.186972	0.683995
57	1	0	3.556557	1.942767	3.499787
58	1	0	-4.927973	0.761979	-3.334808
59	1	0	-5.713324	2.332707	0.586111
60	1	0	-1.094576	6.590856	0.933467
61	1	0	0.530825	3.694075	3.661107
62	15	0	-1.646659	-1.108060	0.841128
63	8	0	-2.381538	-0.062651	1.870132
64	1	0	-3.114803	-0.449105	2.373359
65	6	0	-1.410645	-2.593771	1.867421
66	6	0	-1.251449	-3.845404	1.263196
67	6	0	-1.258330	-2.466006	3.250270
68	6	0	-0.947792	-4.958262	2.039172
69	1	0	-1.355352	-3.949519	0.186347
70	6	0	-0.964147	-3.586618	4.025633
71	1	0	-1.362316	-1.493898	3.723497
72	6	0	-0.805512	-4.830694	3.421371
73	1	0	-0.822630	-5.926604	1.565752
74	1	0	-0.854746	-3.483763	5.100245
75	1	0	-0.570167	-5.701197	4.024885
76	6	0	-2.908065	-1.602012	-0.373820
77	6	0	-2.473979	-2.072475	-1.618645
78	6	0	-4.274506	-1.512971	-0.103154
79	6	0	-3.402367	-2.479091	-2.573212
80	1	0	-1.409008	-2.112367	-1.840286
81	6	0	-5.199924	-1.912124	-1.063462
82	1	0	-4.627365	-1.115157	0.843965
83	6	0	-4.765413	-2.400348	-2.294224
84	1	0	-3.062173	-2.847026	-3.535584

85	1	0	-6.261570	-1.831404	-0.853631
86	1	0	-5.490139	-2.708708	-3.040713

TS $Pd^{2+}(Ph_2POH)_2(Ph_2PO^-)(Ph)$ (16)

234aaa_Hirao_Pd2+_3Ph2POH_-H+_+Ph_M062X_631dp_PCMetoh_SCAN_CP_f_step5_FRQ.log

Standard orientation:

Center Number	Atomic Number	Atomic Type	Coordinates (Angstroms) X	Y	Z
1	6	0	1.718541	-4.528336	-0.912387
2	6	0	1.181592	-3.721354	-1.912976
3	6	0	1.013028	-2.351983	-1.694793
4	6	0	1.361789	-1.774226	-0.465851
5	6	0	1.907052	-2.600094	0.531713
6	6	0	2.081327	-3.961562	0.311637
7	1	0	1.859624	-5.590118	-1.085651
8	1	0	0.900242	-4.152096	-2.869165
9	1	0	0.629251	-1.722090	-2.494928
10	1	0	2.205613	-2.173639	1.486432
11	1	0	2.492610	-4.586495	1.098468
12	46	0	0.147187	-0.111317	0.042495
13	15	0	2.258539	0.159882	-0.845696
14	8	0	2.019880	0.717874	-2.263225
15	15	0	-0.626482	2.061059	-0.680226
16	8	0	0.048111	2.378944	-2.131300
17	1	0	0.830704	1.744356	-2.265117
18	6	0	-2.410106	2.175888	-1.063881
19	6	0	-2.892567	1.446758	-2.158095
20	6	0	-3.312874	2.860376	-0.245774
21	6	0	-4.253822	1.418526	-2.439630
22	1	0	-2.196835	0.896987	-2.787404
23	6	0	-4.678897	2.823239	-0.524611
24	1	0	-2.951112	3.430362	0.605485
25	6	0	-5.150034	2.105626	-1.620727
26	1	0	-6.213646	2.076118	-1.835338
27	6	0	3.876430	-0.720100	-0.950949
28	6	0	4.170343	-1.382229	-2.147950
29	6	0	4.797492	-0.761398	0.097779
30	6	0	5.364092	-2.083077	-2.289842
31	1	0	3.458155	-1.341061	-2.967012
32	6	0	5.990327	-1.469710	-0.043709
33	1	0	4.596978	-0.238870	1.027742
34	6	0	6.274482	-2.132881	-1.234375
35	1	0	7.204162	-2.682032	-1.343074
36	6	0	2.681022	1.506925	0.337686
37	6	0	2.660263	1.326624	1.727622
38	6	0	2.939857	2.773944	-0.188955
39	6	0	2.920760	2.399063	2.574716
40	1	0	2.432228	0.350799	2.152025
41	6	0	3.185171	3.849396	0.664357
42	1	0	2.923500	2.917929	-1.265728
43	6	0	3.180940	3.662866	2.043311
44	1	0	3.363788	4.502628	2.705986
45	6	0	-0.317611	3.565404	0.300870
46	6	0	-0.308317	3.477392	1.694370
47	6	0	-0.076662	4.795358	-0.319723
48	6	0	-0.079415	4.615877	2.465752
49	1	0	-0.466907	2.513478	2.174590
50	6	0	0.166803	5.927310	0.451235
51	1	0	-0.065695	4.852286	-1.404424
52	6	0	0.162399	5.838207	1.844194
53	1	0	0.353574	6.722516	2.443885
54	1	0	6.699821	-1.495964	0.777148

55	1	0	5.585358	-2.589272	-3.224068
56	1	0	3.368138	4.835421	0.249071
57	1	0	2.906285	2.252705	3.650154
58	1	0	-4.617722	0.846606	-3.287117
59	1	0	-5.372780	3.361628	0.113181
60	1	0	0.360866	6.880150	-0.031171
61	1	0	-0.069979	4.543436	3.548722
62	15	0	-1.840697	-1.106324	1.079868
63	8	0	-2.582066	-0.178094	2.223046
64	1	0	-3.362050	-0.588550	2.626635
65	6	0	-1.691266	-2.734277	1.904656
66	6	0	-1.211890	-3.815508	1.155055
67	6	0	-1.954882	-2.906469	3.265929
68	6	0	-1.028872	-5.057322	1.753325
69	1	0	-0.961719	-3.685709	0.103126
70	6	0	-1.772505	-4.153653	3.862627
71	1	0	-2.291160	-2.069777	3.870898
72	6	0	-1.314357	-5.230277	3.107804
73	1	0	-0.650818	-5.886073	1.162775
74	1	0	-1.985321	-4.279901	4.919207
75	1	0	-1.169482	-6.198895	3.574842
76	6	0	-3.153114	-1.426664	-0.156726
77	6	0	-2.770858	-1.939004	-1.402782
78	6	0	-4.500966	-1.157206	0.088182
79	6	0	-3.729153	-2.212856	-2.374463
80	1	0	-1.718254	-2.113016	-1.616881
81	6	0	-5.457314	-1.421258	-0.889646
82	1	0	-4.817668	-0.723514	1.032155
83	6	0	-5.074933	-1.957354	-2.117068
84	1	0	-3.423757	-2.614098	-3.335514
85	1	0	-6.501932	-1.202357	-0.692636
86	1	0	-5.822239	-2.162220	-2.876860

$Pd^0(Ph_2POH)_2$----(Ph_3PO) (17)

234aba_Hirao_Pd2+_3Ph2POH_-H+_+Ph_M062X_631dp_PCMetoh_SCAN_CP_vege.log

Standard orientation:

Center Number	Atomic Number	Atomic Type	Coordinates (Angstroms) X	Y	Z
1	6	0	-0.959321	2.924515	-2.424001
2	6	0	-0.450382	1.698512	-2.847377
3	6	0	-1.012343	0.509528	-2.379503
4	6	0	-2.087786	0.553850	-1.485099
5	6	0	-2.615634	1.788811	-1.087361
6	6	0	-2.046466	2.969764	-1.550845
7	1	0	-0.511472	3.848345	-2.776432
8	1	0	0.385328	1.661708	-3.538763
9	1	0	-0.634030	-0.451288	-2.716122
10	1	0	-3.478494	1.833357	-0.427808
11	1	0	-2.439730	3.925296	-1.219397
12	46	0	0.604747	0.060314	0.036916
13	15	0	-2.727359	-1.022626	-0.864230
14	8	0	-2.180467	-2.188902	-1.668913
15	15	0	1.233508	-2.120530	-0.595109
16	8	0	0.328973	-3.040877	-1.615567
17	1	0	-0.610735	-2.716582	-1.644076
18	6	0	2.858644	-2.243611	-1.436506
19	6	0	3.730280	-1.152368	-1.412879
20	6	0	3.244043	-3.419326	-2.091199
21	6	0	4.977608	-1.233752	-2.032799
22	1	0	3.423749	-0.235390	-0.913044
23	6	0	4.483945	-3.496873	-2.716613
24	1	0	2.565355	-4.267815	-2.113291

25	6	0	5.353244	-2.404261	-2.685865
26	1	0	6.320455	-2.468080	-3.174132
27	6	0	-4.530432	-0.884957	-0.925565
28	6	0	-5.136663	-0.279970	-2.033555
29	6	0	-5.322294	-1.443864	0.082640
30	6	0	-6.523541	-0.235833	-2.129355
31	1	0	-4.525157	0.161117	-2.815990
32	6	0	-6.710324	-1.396584	-0.017242
33	1	0	-4.856910	-1.911657	0.945310
34	6	0	-7.309146	-0.792849	-1.120801
35	1	0	-8.390949	-0.753662	-1.194917
36	6	0	-2.278769	-1.189151	0.884322
37	6	0	-2.463418	-0.158924	1.814508
38	6	0	-1.707240	-2.398063	1.287871
39	6	0	-2.057066	-0.337757	3.133038
40	1	0	-2.913041	0.783292	1.513593
41	6	0	-1.293957	-2.571067	2.607265
42	1	0	-1.555436	-3.188062	0.557351
43	6	0	-1.464905	-1.539526	3.526539
44	1	0	-1.138608	-1.668898	4.553458
45	6	0	1.487598	-3.283467	0.806796
46	6	0	2.113217	-2.822211	1.969686
47	6	0	1.015021	-4.596580	0.756532
48	6	0	2.272731	-3.665841	3.065196
49	1	0	2.454502	-1.789661	2.022248
50	6	0	1.164858	-5.438643	1.858644
51	1	0	0.516868	-4.946954	-0.142676
52	6	0	1.793870	-4.975545	3.012871
53	1	0	1.906462	-5.630264	3.871086
54	1	0	-7.322740	-1.827563	0.767429
55	1	0	-6.991200	0.235734	-2.986936
56	1	0	-0.817626	-3.501859	2.902699
57	1	0	-2.197772	0.460776	3.854657
58	1	0	5.647601	-0.379738	-2.011107
59	1	0	4.777034	-4.408810	-3.227163
60	1	0	0.788110	-6.455906	1.816120
61	1	0	2.757056	-3.299375	3.964831
62	15	0	1.163337	2.024674	1.119425
63	8	0	1.409063	1.873594	2.755205
64	1	0	1.450202	2.727039	3.212262
65	6	0	0.055514	3.492602	1.092638
66	6	0	0.304279	4.633855	0.325046
67	6	0	-1.136360	3.411018	1.825610
68	6	0	-0.621382	5.676351	0.294440
69	1	0	1.221619	4.713630	-0.251122
70	6	0	-2.056965	4.453826	1.800019
71	1	0	-1.339390	2.523769	2.421433
72	6	0	-1.800283	5.590023	1.031754
73	1	0	-0.417493	6.559071	-0.303308
74	1	0	-2.973411	4.380929	2.376949
75	1	0	-2.517644	6.403982	1.008351
76	6	0	2.730438	2.751136	0.507907
77	6	0	3.043798	2.596197	-0.847291
78	6	0	3.609818	3.451767	1.337556
79	6	0	4.212426	3.146922	-1.368857
80	1	0	2.369195	2.031187	-1.489302
81	6	0	4.785179	3.990498	0.819467
82	1	0	3.387707	3.571608	2.394158
83	6	0	5.085561	3.842022	-0.533821
84	1	0	4.446274	3.023429	-2.421447
85	1	0	5.466730	4.526374	1.472108
86	1	0	6.002037	4.262126	-0.935110

$Ph_3P{=}O$ (1a)

250aaa_Hirao_Ph3PO_M062X_631dp_PCMetoh.log

Standard orientation:

Center Number	Atomic Number	Atomic Type	Coordinates (Angstroms) X	Y	Z
1	6	0	-3.218735	2.784508	-0.855787
2	6	0	-2.740280	2.981676	0.437613
3	6	0	-1.778517	2.121469	0.961327
4	6	0	-1.293856	1.060991	0.191117
5	6	0	-1.782641	0.861056	-1.104633
6	6	0	-2.742088	1.723976	-1.625693
7	1	0	-3.969065	3.453859	-1.263795
8	1	0	-3.117519	3.802193	1.038877
9	1	0	-1.402117	2.257046	1.970757
10	1	0	-1.433096	0.022245	-1.700743
11	1	0	-3.122734	1.565317	-2.629102
12	15	0	0.008852	0.031751	0.926798
13	8	0	0.046317	0.161865	2.425566
14	6	0	1.571694	0.568265	0.173341
15	6	0	2.568107	1.049384	1.023873
16	6	0	1.790129	0.530388	-1.208475
17	6	0	3.780192	1.491814	0.496360
18	1	0	2.382298	1.072090	2.093203
19	6	0	3.000665	0.971380	-1.731567
20	1	0	1.021401	0.151369	-1.876945
21	6	0	3.995141	1.452612	-0.878504
22	1	0	4.938787	1.796312	-1.289589
23	6	0	-0.291602	-1.648730	0.310666
24	6	0	-1.600338	-2.147478	0.295423
25	6	0	0.770625	-2.485917	-0.045365
26	6	0	-1.842345	-3.464657	-0.080097
27	1	0	-2.430831	-1.502554	0.569868
28	6	0	0.525122	-3.804175	-0.421180
29	1	0	1.788853	-2.108535	-0.033429
30	6	0	-0.779377	-4.292171	-0.440070
31	1	0	-0.969208	-5.318627	-0.736370
32	1	0	3.170464	0.939800	-2.802559
33	1	0	4.554307	1.865219	1.158481
34	1	0	1.351971	-4.448385	-0.700651
35	1	0	-2.858208	-3.844887	-0.094599
